# Actionable perturbations of damage responses by TCL1/ATM and epigenetic lesions form the basis of T-PLL

**DOI:** 10.1038/s41467-017-02688-6

**Published:** 2018-02-15

**Authors:** A. Schrader, G. Crispatzu, S. Oberbeck, P. Mayer, S. Pützer, J. von Jan, E. Vasyutina, K. Warner, N. Weit, N. Pflug, T. Braun, E. I. Andersson, B. Yadav, A. Riabinska, B. Maurer, M. S. Ventura Ferreira, F. Beier, J. Altmüller, M. Lanasa, C. D. Herling, T. Haferlach, S. Stilgenbauer, G. Hopfinger, M. Peifer, T. H. Brümmendorf, P. Nürnberg, K. S. J. Elenitoba-Johnson, S. Zha, M. Hallek, R. Moriggl, H. C. Reinhardt, M.-H. Stern, S. Mustjoki, S. Newrzela, P. Frommolt, M. Herling

**Affiliations:** 1 Department of Internal Medicine, Center for Integrated Oncology (CIO) Köln-Bonn, University of Cologne (UoC), Köln, 50937 Germany; 2 Excellence Cluster for Cellular Stress Response and Aging-Associated Diseases (CECAD), UoC, Köln, 50937 Germany; 3 CMMC, Center for Molecular Medicine University of Cologne (UoC), Köln, 50937 Germany; 4 Senckenberg Institute of Pathology, Goethe-University, Frankfurt/M., 60590 Germany; 5 Hematology Research Unit Helsinki, Department of Medicine and Clinical Chemistry, University of Helsinki and Helsinki University Central Hospital (HUCH), Helsinki, 00260 Finland; 6 Institute of Animal Breeding and Genetics, University of Veterinary Medicine; Ludwig Boltzmann Institute for Cancer Research, Medical University of Vienna, Vienna, 1210 Austria; 7 Department of Hematology, Oncology, and Stem Cell Transplantation, RWTH Aachen University Medical School, Aachen, 52074 Germany; 8 Cologne Center for Genomics, UoC, Germany, Institute of Human Genetics, University of Cologne (UoC), Köln, 50937 Germany; 9 Duke University Medical Center, Durham, 27708 NC USA; 10 MLL Munich Leukemia Laboratory, Munich, 81377 Germany; 11 Department III of Internal Medicine, University Hospital Ulm, Ulm, 89081 Germany; 12 Department of Internal Medicine, Bone Marrow Transplantation Unit, Medical University of Vienna, Vienna, 1090 Austria; 13 Department of Translational Genomics, UoC, Köln, 50937 Germany; 14 Department of Pathology and Laboratory Medicine, University of Pennsylvania, Perelman School of Medicine, Philadelphia, 19104 PA USA; 15 Institute for Cancer Genetics, Department of Pathology and Cell Biology, Herbert Irving Comprehensive Cancer Center, Division of Pediatric Oncology, Department of Pediatrics, Columbia University Medical Center, Columbia University, New York, 10032 USA; 16 INSERM U830, Institut Curie, PSL Research University, Paris, 75013 France; 17 Bioinformatics Core Facility, CECAD, UoC, Köln, 50937 Germany

## Abstract

T-cell prolymphocytic leukemia (T-PLL) is a rare and poor-prognostic mature T-cell malignancy. Here we integrated large-scale profiling data of alterations in gene expression, allelic copy number (CN), and nucleotide sequences in 111 well-characterized patients. Besides prominent signatures of T-cell activation and prevalent clonal variants, we also identify novel hot-spots for CN variability, fusion molecules, alternative transcripts, and progression-associated dynamics. The overall lesional spectrum of T-PLL is mainly annotated to axes of DNA damage responses, T-cell receptor/cytokine signaling, and histone modulation. We formulate a multi-dimensional model of T-PLL pathogenesis centered around a unique combination of *TCL1* overexpression with damaging *ATM* aberrations as initiating core lesions. The effects imposed by TCL1 cooperate with compromised ATM toward a leukemogenic phenotype of impaired DNA damage processing. Dysfunctional ATM appears inefficient in alleviating elevated redox burdens and telomere attrition and in evoking a p53-dependent apoptotic response to genotoxic insults. As non-genotoxic strategies, synergistic combinations of p53 reactivators and deacetylase inhibitors reinstate such cell death execution.

## Abstract (web summary)

T-cell prolymphocytic leukemia (T-PLL) is a rare malignancy with a poor prognosis. Here, the authors investigate the genomic landscape, gene expression profiles and functional mechanisms in 111 patients, highlighting TCL1 overexpression and ATM aberrations as core lesions which co-operate to impair DNA damage processing.

## Introduction

T-cell prolymphocytic leukemia (T-PLL) is the most frequent mature T-cell leukemia^1^, yet with an incidence of ≈0.6/million in Western countries, it is still an orphan disease. It typically presents in the 6–7th decade of life at stages of exponentially rising lymphocyte counts in peripheral blood (PB) accompanied by hepato-splenomegaly, lymphadenopathy, and bone marrow involvement^1,2^. Its chemotherapy-refractory behavior adds to an inherent very poor prognosis (survival usually <2–3 years)^1,3,4^. Even after common responses to the monoclonal antibody alemtuzumab, eventually all patients relapse^3^. A major reason for the limited therapeutic options to accomplish sustained clonal eradication in T-PLL is our rudimentary understanding of its key disease mechanisms and molecular vulnerabilities.

Karyotypes of T-PLL are often complex^2,5–7^ and include recurrent rearrangements at chromosome (chr.)14, resulting in juxtaposition of *TCL1A (T-cell leukemia/lymphoma 1A)* at 14q32.1 to T-cell receptor (TCR) gene enhancers^8^. This prevents physiological post-thymic silencing of *TCL1A*. *TCL1A* is the namesake of a 3-paralogue family^9^, further including *TCL1B* and *MTCP1*. The X-chromosomal *MTCP1* is involved in rare T-PLL carrying the *t*(X;14) translocation. Transgenic (tg) mouse models emulating human T-PLL illustrate the T-cell oncogenic potential of *TCL1A*^10^ and *MTCP1*^11^. Currently, the best established function of the 14 kDa TCL1A protein is an adapter-like engagement in kinase complexes, formed upon antigen-receptor input, resulting in enhanced pro-survival signaling^5,12^.

Deletions of chr.11q leading to losses of the tumor suppressor *ataxia telangiectasia mutated (ATM)*, as well as amplifications at chr.8q represent additional highly prevalent abnormalities in T-PLL^2,5–7^. While the sporadic form of T-PLL had been associated with somatic *ATM* mutations^13,14^, it can also arise in cancer-predisposed adolescents with *ataxia telangiectasia (A-T)* that carry germline *ATM* inactivations^15^. ATM governs the maintenance of genomic integrity by orchestrating a proper DNA damage response (DDR), including double-strand break (DSB) repair, cell cycle control, and apoptosis regulation^16,17^. An ATM-dependent response to DSBs activates p53 to enforce the G1 checkpoint for repair. Metabolic or redox-homeostatic roles (e.g., regulation of levels of reactive oxygen species (ROS)) are newly recognized functions of ATM^18^. There are also non-canonical DDRs in the absence of DNA damage, i.e., triggered by telomere, mitotic, replicative, or oxidative stressors^19^.

Several series of genomic and transcriptomic profiling already provided important insights into the genetic landscape of T-PLL (data summarized in Supplementary Table 1). However, beyond the implicated involvements of *TCL1A*, *ATM*, and *JAK/STAT* genes^20–24^, there is still an incomplete understanding of their phenotypic impacts and their molecular interplay towards T-PLL. Here we report an integrated genetic and functional study on a large T-PLL patient cohort to delineate the spectrum of alterations and their mechanisms in T-cell transformation. For relevant associations, we selected treatment-naive samples from patients that were included in prospective trials or that were documented in a nationwide registry, providing thorough clinical, immunophenotypic, and cytogenetic data (in part provided in Supplementary Data 1, Supplementary Fig. 1, Methods section).

As the dominant alterations of T-PLL’s molecular make-up, we describe here a unique combination of TCL1-overexpression and damaging *ATM* lesions. We characterize this functionally synergistic interaction to significantly contribute to T-PLL’s specific phenotype of impaired proximal DNA damage processing and abrogated p53-mediated cell death execution. We extract from that targetable vulnerabilities and finally present a model of T-PLL evolution resolved for pivotal genetic alterations integrated with its landmarks of cellular dysfunctions.

## Results

### The hallmarks of dysregulated TCL1A and T-cell activation

**Fig. 1:**
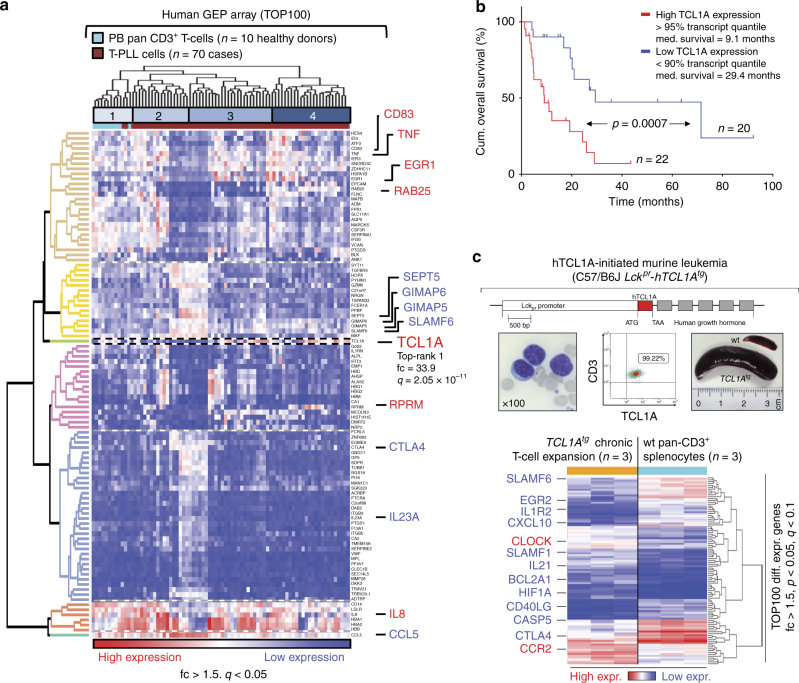
Altered expression of *TCL1A* and T-cell signaling modulators. **a** Heat map: differentially expressed genes and unsupervised sample clustering (#1–#4) in primary human T-PLL vs. normal peripheral blood (PB) T-cells with the top-scoring *TCL1A* and other genes regulating T-cell (receptor) signaling and growth. Confirmatory qRT-PCRs are in Supplementary Fig. 2c. Patient clusters #2 and #3 with median leukocyte counts of 126.5 × 10^3^/μL vs. 58.0 × 10^3^/μL (*p* = 0.068, Student’s *t*-test) significantly differed by expression of *TCL1A* (fc = 3.94, *p* = 0.024) and *RAB25* (fc = −1.7, *p* = 0.023, Student’s *t*-tests; see Supplementary Fig. 2e for *RAB25-*based regression model).** b** Kaplan–Meier plot of disease-specific overall survival (OS; log-rank test, time from diagnosis to event) of uniformly treated T-PLL patients stratified by a 2-tier of the elevated *TCL1A* mRNA expression (*n* = 42, excluding 5% quantile ‘buffer’). Note that ‘low’ levels are still above those of normal T-cells. The cases with high *MTCP1* expression levels (carrying a *t*(X;14)) are exclusively found in the subset of T-PLL with *TCL1A* transcripts below the 90th quantile. **c** TCL1-initiated mouse model of T-PLL. Top: *Lck*^*pr*^*-hTCL1A* allele-targeting construct used^10^; below: leukemic PB (left and mid panel) and splenomegaly (right) at overt disease stage. Heatmap: differential gene expression profiles of murine splenic CD8^+^ T cells at chronic stage (further data in Supplementary Fig. 2f, g and Supplementary Data 3). Comparison: normal splenic CD3^+^ T-cells from C57BL/6 (background- and age-matched wild type) animals (3 arrays from T-cell pools of three mice each (total *n* = 9)). Resemblance to human T-PLL (Supplementary Data 3) is exemplified by downregulation of *CTLA4* (fc = −18.6, *p* = 4.79 × 10^−3^) and *SLAMF6* (fc = −3.96; *p* = 0.03, Student’s *t*-tests)

Array-based gene expression profiles (GEPs) of PB-isolated tumor cells from 70 T-PLL exhibited a differential expression (fold-change (fc) > 1.5, *q* < 0.05) of 2569 probes as compared to CD3^+^ pan T-cells isolated from 10 healthy individuals. Of all genes, *TCL1A* showed the highest dysregulation (fc = 33.9; *q* = 2.05 × 10^−11^, Student's *t*-tests; Fig. 1a). Importantly, as we previously implicated TCL1A as an amplifier of T-cell signaling input^5^, its upregulation was accompanied by deregulations of TCR pathway modulators, suggesting a net enhancement of antigen receptor and cytokine signaling in T-PLL. This included reduced expressions of the negative-costimulatory *CTLA4* (fc = −6.92; *q* = 2.59 × 10^−7^), of the repressive T–T homotypic receptor *SLAMF6* (fc = −3.72; *q* = 2.06 × 10^−9^), or of the T-cell pro-apoptotic *GIMAPs* (*GIMAP5* fc = −3.34; *q* = 1.82 × 10^−10^), as well as overexpression of *TNF* (fc = 9.98; *q* = 1.27 × 10^−8^), also known to shape TCR signals (Fig. 1a, Supplementary Data 2). Upregulation of immunosuppressive *CD83* (fc = 5.69; *q* = 2.70 × 10^−8^) indicates additional immune evasive properties.

Ingenuity pathway analysis (IPA) assigned the differentially expressed genes to significantly enriched clusters of proliferation, cell cycle, survival, chemotaxis, immune responses, and intermediates (i.e., ROS) of signal transduction (Supplementary Fig. 2a, Supplementary Data 2). Gene set enrichment analysis (GSEA)^25^ highlighted target genes of the transcription factor *MYC*, signatures of γ-irradiation responses, or epigenetic remodeling (Supplementary Fig. 2b). We confirmed the deregulated expression of genes associated with T-PLL in meta-comparisons with small published cohorts at the global^20^ (GSEA; Supplementary Fig. 2b) and gene-specific (e.g., *CDKN1B*^26^) level (Supplementary Fig. 2c). qRT-PCRs validated the differential expression for all of 21 selected transcripts (Supplementary Fig. 2c).

The other *TCL1* family members were consistently overexpressed as well: *TCL1B* (fc = 4.53; *q* = 1.49 × 10^−4^) and *MTCP1*^*p13*^ (fc = 2.65; *q* = 2.52 × 10^−3^; Supplementary Fig. 2c). Overall, *TCL1* family expression in 92.9% of T-PLL correlated well with the cytogenetic detection of locus rearrangements in 94.6% of cases (Supplementary Fig. 2d). *TCL1A* levels correlated inversely with patient survival (*p* = 0.7 × 10^−3^, log-rank test, Fig. 1b). Further regression modeling provided a refined score, including *RAB25* and *KIAA1211L* to predict less vs. more aggressive clinical courses (Supplementary Fig. 2e, Methods section).

Postulating an initiating role of dysregulated *TCL1* genes, we evaluated changes in GEPs in mice with early-onset T-lineage specific overexpression of human (h) TCL1A (Fig. 1c). Already sub-clinical ‘chronic’ phase expansions (Supplementary Fig. 2f, g) from spleens of these *Lck*^*pr*^*-hTCL1A*^*tg*^ mice revealed a differential downregulation of *CTLA4* and *SLAMF6* (Fig. 1c) and many other changes observed in human T-PLL (fc > |1.5|; *p* < 0.05; Supplementary Data 3). This signature of T-cell activation in conjunction with TCL1A-drive was preserved at the ‘exponential’ murine disease stage with additional deregulation of prominent markers of transformation (Supplementary Fig. 2f, g, Supplementary Data 3).

Whole-transcriptome sequencing (WTS) of 15 T-PLL vs. 4 normal T-cell isolates confirmed the prominent *TCL1A* overexpression and other top-scorers from the array-based GEP analyses (Supplementary Figs. 3a, b, Supplementary Data 4). Importantly, alternative *TCL1A* transcripts were detected (Supplementary Fig. 3c). Tumor-associated alternative splicing was indicated by 1927 segments in 1091 genes (|log_2_-fc| > 5, *q* < 0.01, *χ*^2^-test) that showed differential exon usage in T-PLL over healthy T cells (Supplementary Data 5). The most significantly affected genes were involved in TCR/cytokine signaling and p53 associated apoptosis regulation (Supplementary Fig. 3d, Supplementary Data 5).

### Copy number losses of *ATM* and novel gains at chromosome 8q

**Fig. 2:**
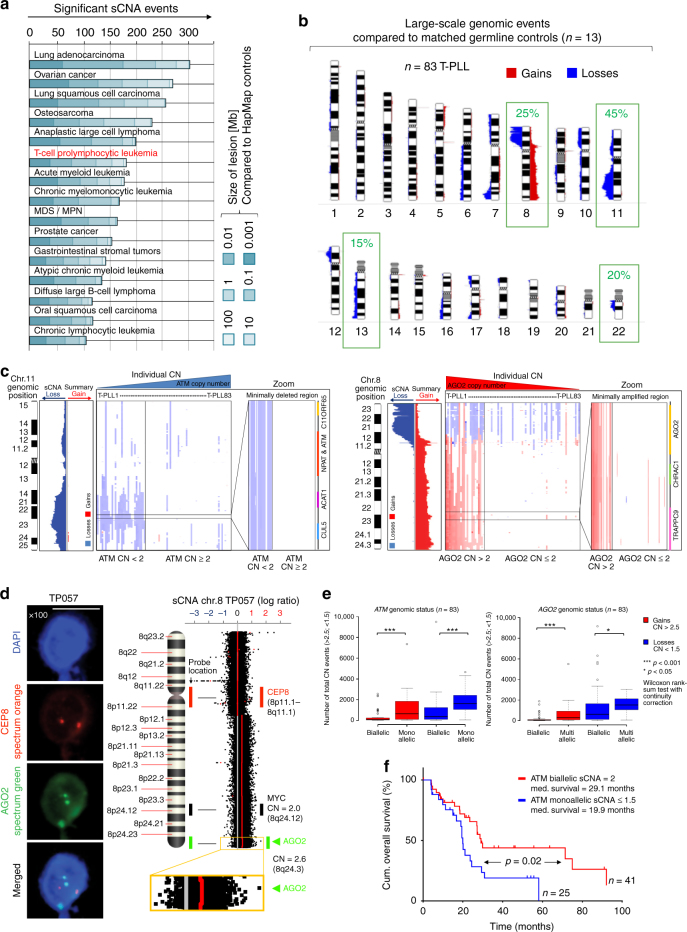
Large-scale genomic aberrations dominantly involve losses of *ATM* on chr.11q and gains of *AGO2* and *MYC* on chr.8q. **a** Number of differentially sized somatic copy-number alterations (sCNA) in this T-PLL cohort (*n* = 83) compared to publically available Affymetrix SNP 6.0 primary array data sets (all HapMap controlled, meta-analysis procedure in Methods section). **b** Ideograms with average abundance of large-scale genomic lesions (Supplementary Fig. 4a, Supplementary Data 6 for GISTIC2.0 analyses). **c** Minimally deleted region (MDR) on chr.11 centering on *ATM* and minimally amplified region (MAR) on chr.8 defined by *AGO2* (for MDRs on chr.13 and chr.22 see Supplementary Fig. 4c). **d** Left: verification of *AGO2* amplification in T-PLL case TP057 with biallelic *MYC* (CN = 2) using FISH (scale bar = 5 µm). Right: circular binary segmentation (CBS) with *p* ≤ 0.01 detects *AGO2*, but not *MYC* as significantly amplified. **e** Total number of significant global gains (red) and losses (blue) in T-PLL ‘monoallelic’ (CN ≤ 1.5), ‘biallelic’ (CN = 2), and ‘multiallelic’ (CN ≥ 2.5) for ATM / AGO2 excluding these affected regions. Representations: boxes as interquartile range (IQR); thick line as the mean, whiskers as lower and upper limits. Lower limit = *x*_0.25_–1.5 × IQR. Upper limit = *x*_0.75_ + 1.5 × IQR. Values above or below are potential outliers and marked as *O* (***p < 0.001, *p < 0.05, Wilcoxon rank-sum test with continuity correction). **f** Different OS across 66 T-PLL subjects stratified by *ATM* CN (log-rank test)

Based on the detected average abundance of large-fragment genomic lesions in our set (*n* = 83), T-PLL is positioned near the ‘complex’ end of the spectrum of somatic copy-number alterations (sCNAs) of hematopoietic and solid cancers (Fig. 2a). The most frequent sCNAs (compared to pooled germlines from 13 cases / HapMap controls) were found at chr.11 (37%/52%), chr.8 (29%/42%), chr.22 (24%/24%), and chr.13 (14%/14%) (Fig. 2b). GISTIC2.0 analyses underlined the significance of those prominent lesions (Supplementary Data 6, Supplementary Fig. 4a). The inv(14) and *t*(14;14), detected in 93% of this set by FISH/karyotyping, were predominantly copy-neutral. We identified recurrent (affected in >20% of cases) gains (CN > 2.5) in 637 genes and losses (CN < 1.5) in 1685 genes (Supplementary Fig. 4b, Supplementary Data 7). The presence of complex karyotypes (>3 large-scale aberrations), a determinant of poor-outcome subsets in a recent series of T-PLL^6^ and in other leukemias, was a rather uniform feature here (89.5%).

Aberrations on chr.11 and chr.8 are described for T-PLL and have been intuitively linked to alterations of *ATM* and *MYC*. We defined here the minimally deleted and amplified regions (MDR/MAR) of these most prominent hot-spots compared to patient-derived germlines (Fig. 2c, Supplementary Fig. 4c). The chr.11 MDR was represented by strictly monoallelic losses of *ATM* found in all MDR-affected cases (31/83, 37.4%, average CN = 1.79; HapMap controlled). Often co-deleted adjacent to this MDR were the P53-suppressor network micro-RNAs miR34b/c (Supplementary Fig. 4b). Genes encoding for downstream effectors of ATM were disrupted in a minority of T-PLL (e.g., *CHEK2* 13.3%; *TP53* 4.8%).

In contrast to the assumption of *MYC* being the primary target of the chr.8 associated gains, we identified *AGO2* (*argonaute RISC catalytic component 2*) to define this MAR, that was present in 28.9% of cases (51.2% when HapMap controlled; average CN = 2.22; Fig. 2c). *MYC* gains were involved in only 70.8% of T-PLL harboring a MAR on chr.8 (average CN = 2.17; Supplementary Fig. 4b, Supplementary Data 7). The *AGO2* gains were validated using a FISH probe and were associated with *AGO2* overexpression (Fig. 2d, Supplementary Fig. 4d, e). *AGO2* is frequently overexpressed in cancer^27^ and has been increasingly considered a pivotal mediator of oncogenic miR/siRNA biogenesis^28^ with emerging functions in chromatin remodeling and alternative splicing^29^. In agreement with current paradigms of cancer gene activity^30^ and further implicating a relevance of altered miR/siRNA processing factors in T-PLL, we identified uniparental disomies (UPDs) of *AGO1/-3/-4* (all on chr.1) in 68.7% of cases (*n* = 57/83; against HapMap; Supplementary Data 7).

In accordance with their molecular functions^31,32^, *ATM* losses and *AGO2* over-representations were each associated with a higher degree of CN-lesional genomic complexity outside their own affected regions (Fig. 2e) and with specific GEPs, e.g., chr.11 MDR with dysregulated *SLAMF6* or chr.8 MAR with reduced *CTLA4* (Supplementary Fig. 5a–c, Supplementary Data 8,9). Among the prominent CN lesions, *ATM* sCNAs were of negative prognostic impact (*p* = 0.02, log-rank test, Fig. 2f; similar to lower ATM transcript levels Supplementary Fig. 5d).

Generally, CN losses/gains were not strictly linked to altered expressions of the affected genes (Supplementary Figs. 4e, 6a–c, 7), likely because of not depicted regulatory aspects (e.g., allele-dominances or LOH scenarios). Conversely, the reductions in *ATM* transcript levels were also found in chr.11 MDR-negative cases and the uniformly increased MYC expression was independent of the presence of a chr.8 gain (Supplementary Figs. 4e, 6a–c, Supplementary Data 2). Both suggest additional modes of dysregulation beyond sCNAs. This was recapitulated in TCL1A-initiated murine T-PLL: although the proliferations of *Lck*^*pr*^*-hTCL1A*^*tg*^ mice lacked *ATM* and *MYC* sCNAs, they harbored reduced and increased expression of these genes, respectively (Supplementary Fig. 6d, e).

### New gene fusions in high-resolution DNA and transcript assessments

**Fig. 3:**
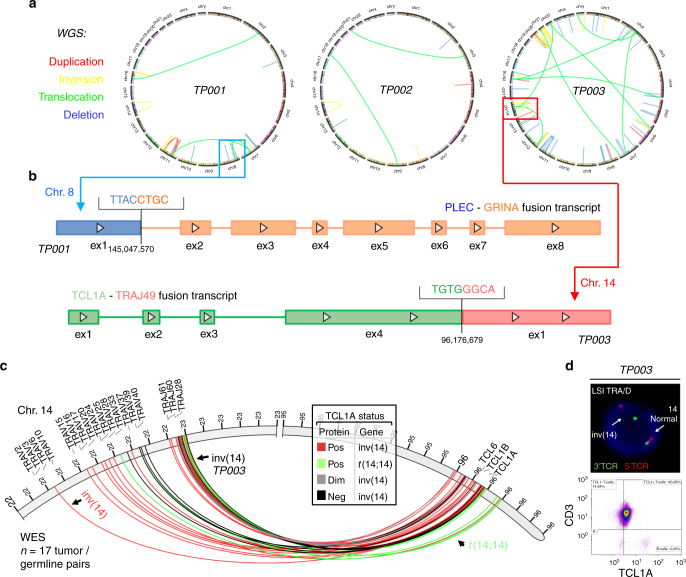
Novel structural variations and fusion transcripts. **a** Whole-genome sequencing (WGS) of 3 T-PLL t/g-pairs to map intra- and inter-chromosomal translocations: 6 lesions affecting 4 distinct chromosomes (TP001), 10 lesions affecting 5 chromosomes (TP002), and 31 lesions affecting 10 chromosomes (TP003); see Supplementary Fig. 8a for whole-exome sequencing (WES) derived data. **b** Fusion transcripts (*n* = 96, TopHat-Fusion and oncofuse algorithms) identified by whole-transcriptome sequencing (WTS) of 15 T-PLL compared to healthy donor T-cells (*n* = 4). Two examples: *PLEC-GRINA* from aberrations on chr.8 and *TCL1A-TRAJ49* (*TCRα joining element 49*) from an inv(14). **c** Mapping of breakpoints involved in the inv(14) or *t*(14;14) derived from WES data on 17 t/g-pairs. Four cases carried each 2 distinct breakpoints. **d** The FISH-confirmed inv(14) of TP003 (see (**b**); *TCL1A-TRAJ49*) was associated with intermediate-level TCL1A protein expression (flow cytometry). Further validation is provided in Supplementary Fig. 8b with proof of a viable transcript and co-expression of neighboring *TCL1B*

Somatic intra- and inter-chromosomal structural variations (SVs) detected by whole-genome sequencing (WGS, *n* = 3) revealed a high heterogeneity among cases. COSMIC listed SVs recurrently affected chr.8, 11, 14, 16, and 21 (Fig. 3a, Supplementary Data 10). Fusion transcripts identified in WTS (15/15 cases; Supplementary Data 11; by TopHat-Fusion) with corresponding SVs in whole-exome sequencing (WES) data (*n* = 17) included the hybrids: *JAK2* (chr.9)* - TCF3* (chr.19), *TRIM22* (chr.11) - *JAK2*, and *KANSL1 *-* ARL17A* (both chr.17), each in 1 case. Three chr.8-intrinsic fusions involving *PLEC* with varying partners (*GRINA*, *CYHR1*, and *SHARPIN*) (Fig. 3b) were likely the result of the complex rearrangements at chr.8. A SEPT-ABL1 hybrid reported in an anecdotal T-PLL^33^ or fusions found in nodal mature T-cell lymphomas^34^ were not identified.

Overall, the inv(14) or *t*(14;14) were the most common structural aberrations (3/3 by WGS, 14/17 by WES; Fig. 3c, Supplementary Fig. 8a, Supplementary Data 10). In TP003 the inv(14) links *TCL1A* to *TRAJ49*. This fusion transcript was validated with multiple callers (Methods section) and by RT-PCR combined with Sanger sequencing. It is the first report of a *TCL1A* fusion instead of the usual in-trans positioning, with validated mRNA and protein expression (Fig. 3b, d, Supplementary Fig. 8b). In conjunction with the data on sCNAs, there were no indications for chromothriptic events^35,36^.

### Prominent patterns and pathways of the mutational landscape

Having identified alterations in *TCL1A*, *ATM*, *AGO2*, or *MYC* as highly prevalent lesions, we next assessed the contribution of mutations (single-nucleotide variants (SNVs) and small indels (insertions and deletions)). Samples from 72 patients were subjected to WGS (three tumor/germline (t/g)-pairs, 1 tumor ‘single’), WES (17 t/g-pairs; 37 singles), targeted-amplicon sequencing (TAS; *n* = 18), and Sanger resequencing (platform overlap in Supplementary Fig. 1a). Purification and separation of t/g-paired material in a 2-step cell-sorting procedure ensured average tumor purities >98% and contamination rates <2% in germline isolates (Supplementary Fig. 1b). This high purity, together with the generally diploid karyotype of T-PLL cells (overall CN 1.97 based on TitanCNA and the 17 t/g WES pairs) facilitated specific somatic calls and reliable calculations of variant allele fractions (VAFs) and cellular prevalences (formerly ‘cancer cell fractions’ (CCFs)) for estimations of (sub)clonal sizes (Supplementary Data 12). To identify mutations likely to be biologically relevant, we applied various stringent thresholds, e.g., 8-oxoguanine (8-Oxo-G) allelic imbalance filter^37,38^, false-discovery-rate (FDR) based MuSiC^39^ analysis, required COSMIC data base hit, minimum population frequency (PopFreq) <0.01, and prediction to be damaging (SIFT, PolyPhen2,PROVEAN).

**Fig. 4:**
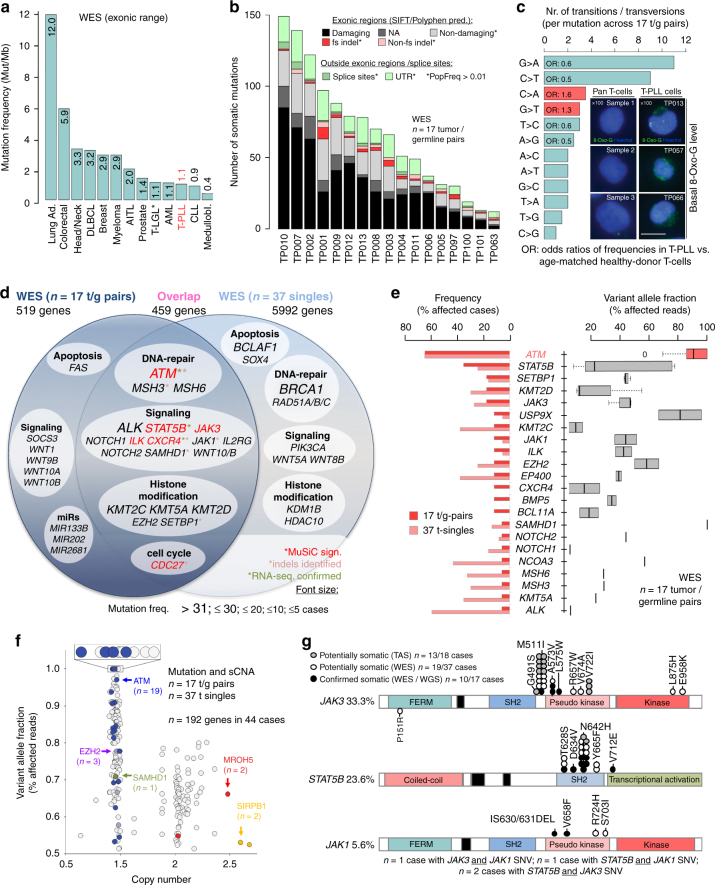
The mutational landscape of T-PLL - recurrent patterns and pathways. **a** WES of 17 T-PLL t/g-pairs: meta-analysis (details in Methods section) comparing the mutation frequency in T-PLL to other malignancies (* 2 cases with T-LGL were sequenced as part of this study). **b** Number of somatic SNVs and small indels per t/g-pair resolved for locations and characteristics (also Supplementary Data 12); overall 1141 distinct SNVs and indels: 9 frameshift insertions, 22 frameshift deletions, 17 non-frameshift deletions, 10 non-frameshift insertions, 745 non-synonymous, 20 splice sites, 79 ncRNA CDS, 38 stop-gains, 1 stop-loss, 178 within UTRs, and 22 alterations of unknown function. **c** Bars: median numbers of base exchanges calculated in the 17 t/g-pair WES data sets revealed a relative enrichment of C:G > A:T transversions (Odds ratios (ORs) 1.3 for G > T and 1.6 for C > A) compared to age-matched normal memory T-cell samples, while C:G > T:A are underrepresented in T-PLL (*p* = 0.008, Fisher’s count test). The ranks of substitutions were significantly different, with C:G > A:T at 4/12 and 3/12 in T-PLL and at 6/12 and 8/12 in donors age-matched memory T-cells (*p* = 0.0002; Wilcoxon test; Supplementary Fig. 9b). Panels: Immunofluorescence microscopy using an 8-OxoG specific antibody in T-PLL cells (right) vs. healthy-donor derived pan T-cells (left) (scale bar = 5 µm). See Supplementary Fig. 9d for quantification and technical controls. **d** Mutated genes (including SNVs and indels; frequencies are font-size coded) identified in *n* = 17 t/g-pairs and *n* = 37 t-singles by WES. **e** Left: frequencies of T-PLL cases affected by mutations in a selection of genes based on frequencies in the cohorts of 17 t/g-pairs (red) and 37 t-singles (rose). Right: mean VAFs of the selected genes over all mutated cases in the 17 t/g-pairs (interquartile range (IQR); mean with upper and lower limits). **f** Integrated WES and sCNA-profiling data to identify genes with gain-of function (GOF, CN > 2.2, VAF > 0.5) and loss-of-function (LOF, CN < 1.7, VAF > 0.5) aberrations. **g** Missense mutations in *JAK3, STAT5B*, and *JAK1* genes identified by WES and targeted-amplicon sequencing (TAS). Confirmed somatic: t/g-pairs (WES: *n* = 17); potentially somatic: tumor singles (WES: *n* = 37; TAS: *n* = 18)

Characteristic global patterns of mutations: The median rate of exonic somatic mutations in T-PLL of ~1.1 Mut/Mb was similar to other hematologic and solid neoplasms (Fig. 4a; Supplementary Data 12). Regional annotations, e.g., exonic or splice-site, are summarized in Fig. 4b. When compared to other catalogued cancer genomes^40^, the mutational profile of T-PLL revealed striking similarities to the signatures of ‘aging’ (*ρ* = 0.65; *p* = 8.9 × 10^−13^) and ‘smoking’ (*ρ* = 0.62; *p* = 2.1 × 10^−11^, Spearman correlations, Supplementary Fig. 9a). This points at a prominent role of non-predispositional cumulative insults in T-PLL. In fact, we noticed enrichments of C:G > A:T transversions implicating the presence of high-level genotoxic, i.e., oxidative, stress^41^ (Fig. 4c, Supplementary Fig. 9b). Fittingly, immunofluorescence stains for 8-Oxo-G marks of oxidative DNA damage, a major source of deleterious G > T exchanges, indicated higher loads in T-PLL cells over normal T cells (Fig. 4c, Supplementary Fig. 9d; *p* = 0.005, Student’s *t*-test). In further support, levels of ROS induced upon TCR stimulation were higher in primary T-PLL cells than in healthy-donor T cells (Supplementary Fig. 9c; *p* = 0.042, Student’s *t*-test). Since T-PLL cells resemble memory T-cells^5^, a long-lived subset predestined to accumulate high amounts of DNA damage, we also compared the WES-derived mutational profiles of T-PLL to those of memory T-cells isolated from 3 age-matched healthy donors (ages 61, 63, and 65 years), with memory T cells from 3 young donors (22, 28, and 31 years) as controls. It revealed significant differences in the relative distribution of base alterations in specific trinucleotide contexts (*p* = 0.2 × 10^−3^, Wilcoxon test), particularly a higher prevalence of C:G > A:T in T-PLL (Fig. 4c, Supplementary Fig. 9b). Together, these data suggest that oxidative damage in T-PLL is enriched by inefficient repair mechanisms that fail to counteract these specific genotoxic hazards of ‘T-cell aging’.

Pathways recurrently affected: A ranking of the genes affected by SNVs and indels identified in WES t/g-pairs and WES t-singles (Fig. 4d, e, Supplementary Fig. 10a) highlighted *ATM* (64.8%, 35/54 cases) and *STAT5B* (27.8%; 15/54) by highest frequencies. Potential biological significance could also be ascribed to less-often mutated genes based on their clustering in pathways like the DDR, i.e., its branch of mismatch repair (*MSH3*, *MSH6*). Epigenetic modulation was another prominent cluster, e.g., represented by a high incidence of the mutated histone methyl transferases *KMT2C*, *KMT2D*, and *KMT5A* cumulatively in 61% (33/54) of cases. Other recurrently affected pathways were apoptosis/survival signaling and cell cycle regulation (Supplementary Data 12 and GSOA in Supplementary Fig. 10b).

Potential driver mutations: In the 17 WES data sets of paired t/g-samples allowing stringent background estimation, 26 genes were identified as significantly mutated (MuSiC^39^ with FDR < 0.1; Supplementary Data 13), including *ATM*, *JAK3*, *STAT5B*, *ILK*, *CDC27*, and *CXCR4* (Fig. 4d, red asterisks). When pooled with the 37 pseudosomatic singleton WES data sets, genes identified as significantly mutated (*n* = 668 total, Supplementary Data 13) further included *IL7R*, *EZH2, MLH1*, *HIST1H1A, KDM1B*, *FAT2, SAMHD1*, and *FASTKD1*. This confirms the relevance of disturbed DNA repair, cytokine or apoptotic signaling, and epigenetic regulations. Importantly, only a small number of SNVs and indels showed high VAFs / cellular prevalences of 80–100%. This total of 16 genes (1.4%) in 11/17 cases included *SAMHD1*, *USP9X*, or *FASTK*. However, *ATM* was the only gene harboring highly recurrent mutations with a cellular prevalence >80%, thus most likely represents a common driver in T-PLL (Supplementary Data 14, Fig. 4e, Supplementary Fig. 10c for all mutations).

Co-occurrence of mutations and copy-number events: Integration of sCNA and WES data to speculate on selection for dysfunctional targets revealed that 192 of the 6970 mutated genes (including read-throughs) were affected by gain-of-function (GOF, CNV > 2.2, VAF > 0.5) or loss-of-function (LOF, CNV < 1.7, VAF > 0.5) aberrations. Somatic mutations combined with focal gains/losses were found in 72% (*n* = 39/54) of cases. They dominantly included the DDR master regulator *ATM* (19/54 WES cases) and the histone-Lysine N-methyltransferase *EZH2* (3 cases; Fig. 4f, Supplementary Data 15). This emphasizes the particular relevance of genes associated with DNA repair and epigenetic regulation. Further genes simultaneously affected by sCNAs and SNVs/indels, included *POT1*, *JAK1*, and *PCM1*, which are all linked to hematologic malignancies. Associations of SNVs/indels with UPDs were found in 81.5% of cases, affecting 321 genes including *STAT5B* (3 cases) and *ATM* (3 cases). We observed a significant co-occurrence of mutations affecting *STAT5B* and *ATM* (*p* = 0.045, Fisher’s exact test), suggesting an accessory role of *STAT5B* in T-PLL tumorigenesis. Only 9.7% of T-PLL were neither *ATM* nor *JAK/STAT* mutated. Further associations are depicted in the multivariate *q*-value matrix of Supplementary Fig. 10d.

Subclonal *JAK/STAT* variants: The observed high frequencies of mutations in JAK/STAT signaling components, shown previously also in smaller series^21–23,42^, underline their somatic character. However, their low and generally CN-neutral VAFs implicate these lesions rather as subclonal events (Fig. 4e). Combining all sequencing approaches employed here, *IL2RG* (3.7%), *JAK1* (5.6%), *STAT5B* (23.6%), or *JAK3* (33.3%) were mutated in a total of 61.6% of the 72 analyzed T-PLL. These were predominantly mismatch mutations in the SH2 (*STAT5B*) and pseudo-kinase (*JAK1*/*JAK3*) domains (Fig. 4g). Basal phosphorylation states of activating JAK/STAT motifs were elevated in T-PLL over normal T-cells (Supplementary Fig. 11a, b; pSTAT5B^Tyr694^; human: *p* = 0.017; murine h*TCL1A*-tg leukemia: *p* = 0.054, Student’s *t*-test). This was not restricted to *JAK1/JAK3/STAT5B* mutated T-PLL. In fact, genomic losses of negative JAK/STAT regulators like the phosphatases *DUSP4* or *SOCS3*, found in 33 and 8% of our cases, respectively, implicate alternative activating mechanisms. *JAK/STAT* mutated and -unmutated T-PLL showed similar extents of STAT5B phospho-activation in response to interleukins (Supplementary Fig. 11c). To interrogate for a differential activating potential of the *STAT5B* mutations, we overexpressed N642H (most frequent), T628S, D634V, and V712E (outside SH2 domain, not reported for T-PLL) in model cell lines (Supplementary Fig. 11d, e). Particularly N642H conferred elevated basal levels of phospho-STAT5B^Tyr694^. N642H and V712E conveyed a significant pro-survival effect in IL-3 dependent 32D cells (*p* < 0.0001 and *p* = 0.0002, Student's *t*-tests; Supplementary Fig. 11e).

### Mutations in *ATM* reveal clustering in functional domains

**Fig. 5:**
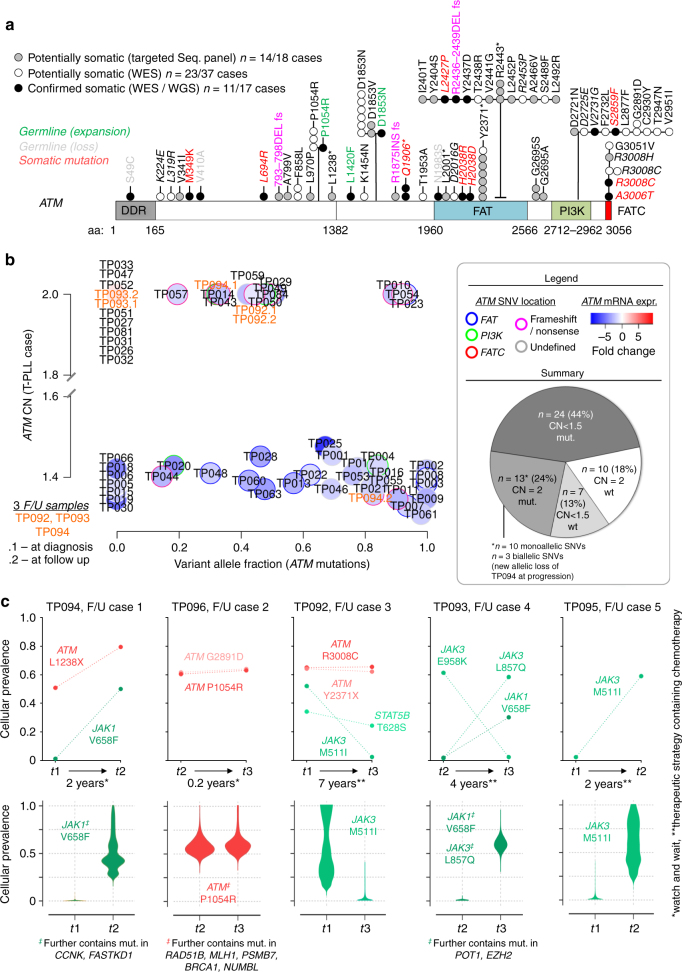
*ATM* sequence variants and dynamics of clonal compositions. **a** *ATM* mutations identified in WES (54 cases) and TAS (18 cases) mapped on the schematic polypeptide strand show clustering in the FAT (21/67 (31%) total mutations) and PI3K (11/67 (16%)) domains (* denotes nonsense mutation). See Supplementary Fig. 12a,b for validations and meta-analysis with published *ATM* mutations in T-PLL. **b** Integration of *ATM* CNs, VAFs, and mRNA levels in 54 T-PLL with complete platform overlap (3 sequential samples: TP092, TP093, TP094). Largest subsets among the 44 CNA/mutation affected cases: LOH genotype (enriched FAT domain mutations; *p* = 0.0079, Fisher’s count test) followed by ATM-mutated/CN-biallelic cases (enriched frameshift or nonsense mutations; *p* = 0.021, Fisher’s count test). UPDs in 3 cases: TP010, TP023, and TP054. **c** Five sequential samples analyzed by WES. Top: VAFs for single mutations are copy-number- and contamination-corrected to obtain cellular prevalences based on Bayesian models (PyClone*;* Supplementary Data 18 for all genes). For illustrative purposes the prevalences of mutations between time points carry dashed connectors for better visual guidance, although such clonal dynamics likely do not follow a strict linear function. There were redundant and prominent changes in relative subclonal ‘sizes’ of *JAK1/JAK3/STAT5B* variants. The increase in cellular prevalence of *ATM*^L1238*^ in treatment-naive TP094 at *t*_2_ was attributable to a loss of the remaining wt-allele (CN < 1.5, Fig. 5b). This was accompanied by a further downregulation of *ATM* mRNA (fc = −1.63 vs. fc = −2.35). Bottom: Clone sizes of clusters (^‡^) containing mutations shown in the top panels (identified via PyClone). The *JAK3* changes in cases F/U4 and F/U5 corroborate the inverse relationship of *JAK3* and *ATM* variants (Supplementary Fig. 10d). For longitudinal changes in GEP and sCNAs see Supplementary Data 16, 17

Variants of *ATM* were detected in 48/72 (67%) of cases (WES and TAS; Fig. 5a). They were mostly missense SNVs (*n* = 42/49 distinct mutations), less frequently nonsense SNVs (*n* = 4/49), or frameshift indels (*n* = 3/49), unlike the predominantly truncating lesions found in *A-T* individuals^43^ (Fig. 5a, Supplementary Fig. 12a). We cataloged lesions at 36 unreported localizations. Previous studies of low sample numbers each individually suggested an unbiased distribution of *ATM* mutations in T-PLL across the molecule. However, our data, enriched for somatic calls, revealed for the first time a clustering of mutations in the FAT (31%) and PI3K (16%) domains. A meta-analysis of our and published data on *ATM* mutations in T-PLL further emphasized this (Supplementary Fig. 12b).

Integrating the data on GE, sCNA, and mutation profiling showed that the vast majority of T-PLL was affected by monoallelic deletions or/and mutations of *ATM* (44/54, 81%; Fig. 5b). These cases generally showed a reduced *ATM* transcript abundance (global fc = −2.32, *q* = 2.2 × 10^−11^ vs. normal T-cells, Student's *t*-test). Generally, downregulated *ATM* expression was associated with significant reductions of 5 of 7 protein encoding transcript variants (WTS analysis of 15 T-PLL vs. 4 healthy T-cell controls; fc > |1.5|; *p*-values from *p* = 3.4 × 10^−5^ to *p* = 0.04, Student's *t*-tests; Supplementary Fig. 12c).

Most frequently, ATM was subject to an LOH event (CN < 1.5; *ATM* mutated, VAF > 20%) (*n* = 24/54, 44%), also reflected by significant co-occurrence of *ATM* mutations with *ATM* sCNAs (*p* = 0.0046; odds ratio 4.33, Fisher’s exact test; Supplementary Fig. 10d). *ATM* expression in the 17 T-PLL with an unmutated *ATM* sequence was unchanged in the CN-biallelic subset (*n* = 10, fc = 1.13), but highly deregulated in the CN-monoallelic cases (*n* = 7, fc = −3.26).

The 10 *ATM* CN-biallelic/unmutated T-PLL included one case with a *TP53* mutation (TP032; *p*.X215Q; VAF = 0.23; CN = 2), one case with a *BRCA1* mutation (TP093), 3 *SAMHD1* mutated cases (TP026, TP027, and TP031), and 3 cases (TP033, TP047, and TP081) with multiple damaging mutations in pro-apoptotic and epigenetic regulators, e.g., *FASTKD1, CASP10, KMT2C,* and *DNMT3A*. The remainder of 2 cases (TP051, TP052) was only subjected to the selected TAS panel.

### Longitudinal changes in clonal compositions

To reconstruct hierarchies of chronological alterations, we analyzed serial samples from diagnosis / before therapy vs. progression / post-therapy relapse of 5 patients (constant sample purities; Supplementary Fig. 13a). Such progression-associated changes or those selected by therapy were most prominent at the global mRNA level (Supplementary Fig. 13b, Supplementary Data 16). Gene CNAs suggested sequential increases in global complexity (1.4-fold, *p* = 0.06, Wilcoxon test, Supplementary Fig. 13c, Supplementary Data 17). As expected, for the majority of gene mutations cellular prevalences remained unchanged, but specific time-point restricted calls (clusters) existed (Supplementary Fig. 13d, Supplementary Data 18). Differential calls at progression or relapse were markedly enriched for genes associated with ‘interleukin-‘ and ‘STAT3 signaling’ (*p* = 0.27 × 10^−4^, hypergeometric tests via ConsensusPathDB, Supplementary Data 19), dictated by dynamics of subclone sizes of mutated *JAK1*, *JAK3*, or *STAT5B* (Fig. 5c). Variants with exclusively increasing cellular prevalences were annotated to ‘ATM-/TP53-regulated transcription of DNA repair genes’ (*p* = 0.00049) and ‘Notch signaling’ (*p* = 0.00065; Supplementary Data 19).

### *TCL1A* and *ATM* as the most recurrently affected genes in T-PLL

**Fig. 6:**
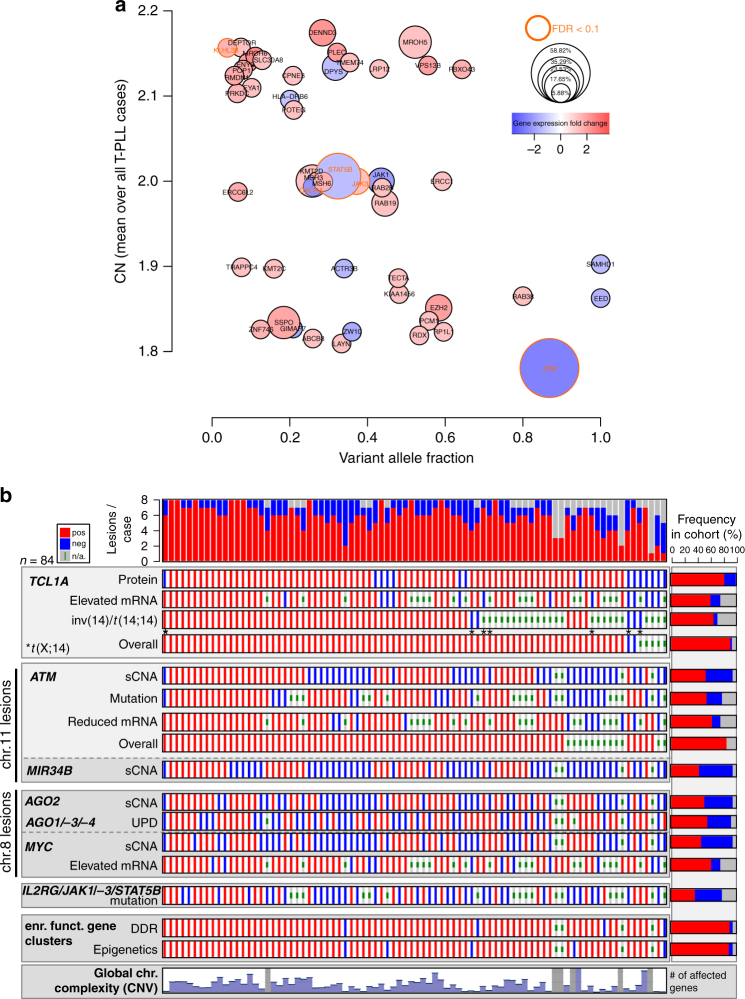
Dysregulated *TCL1* and lesions in *ATM* as the genetic basis of T-PLL. **a** Genetic events across all analyzed T-PLL cases (one circle per gene). *y*-axis: sCNA-affected (CN-mean over all T-PLL, 83 cases); *x*-axis: mutated (mean VAF over all detected mutations, 54 cases); circle size: mutation frequency among all cases; circle border coloring: FDR < 0.1 of mutations; circle color: fc-gene expression (70 cases). Somatic mutations (SNVs and small indels) with at least one damaging prediction were considered. Selection criteria for visualized genes: CN-affected (CN > 2.2; CN < 1.8) and mutated at significant (FDR < 0.1) and/or at prominent clonal (VAF > 0.5; see Fig. 4d) level. *ATM* was the most prominent gene affected by CN losses and mutations of high VAFs, associated with overall mRNA downregulation. **b** Presence of dominant lesions detected in GEP (high/low expression), sCNA (gain/loss), and mutation (present/absent) profiling summarized for 84 T-PLL (red: lesion present, blue: lesion absent, gray: not analyzed). Chromosomal complexity: moderate with <2000 (*n* = 25) and high with >5000 (*n* = 26) sCNA-affected genes. Order of cases according to the number of calls in the following 8 ‘lesional categories’: (1) overall *TCL1 / MTCP1* affected (note that there were no mutations detected in *TCL1* genes), (2) overall *ATM* affected, (3) *AGO2* sCNA present, (4) *AGO1/AGO3/AGO4* UPD present, (5) *MYC* sCNA present or *MYC* mRNA upregulated, (6) *IL2RG*/*JAK1*/*JAK3*/*STAT5B* mutated, and differentially expressed genes associated with epigenetic regulators (7; Supplementary Data 20) or DDR (8; Supplementary Data 21). Each case was affected by at least one of these core lesions

We summarize in Fig. 6a, b the lesional landscape of T-PLL in a global gene-centric integration across all analyzed cases. Dysregulated *TCL1A* mRNA or protein expression and structural variations involving the *TCL1A* or *MTCP1* locus were detected in 94% of samples. Somatic losses and mutations affecting *ATM*, associated with significant mRNA downregulation, were identified as highly prevalent as well (86%). Beyond that, 69% of cases were affected by at least one of the other key aberrancies, i.e., sCNAs of chr.8 or mutations in *JAK/STAT* genes. Finally, aberrant expression of genes associated with the canonical DDR or with epigenetic regulation was inherent to 93% of T-PLL. To further substantiate a disease model, functional studies, i.e., centering on phenotypic outcomes of *ATM* and *TCL1A* deregulation, were undertaken.

### Impaired damage responses implicate a functionally hypomorphic ATM

**Fig. 7:**
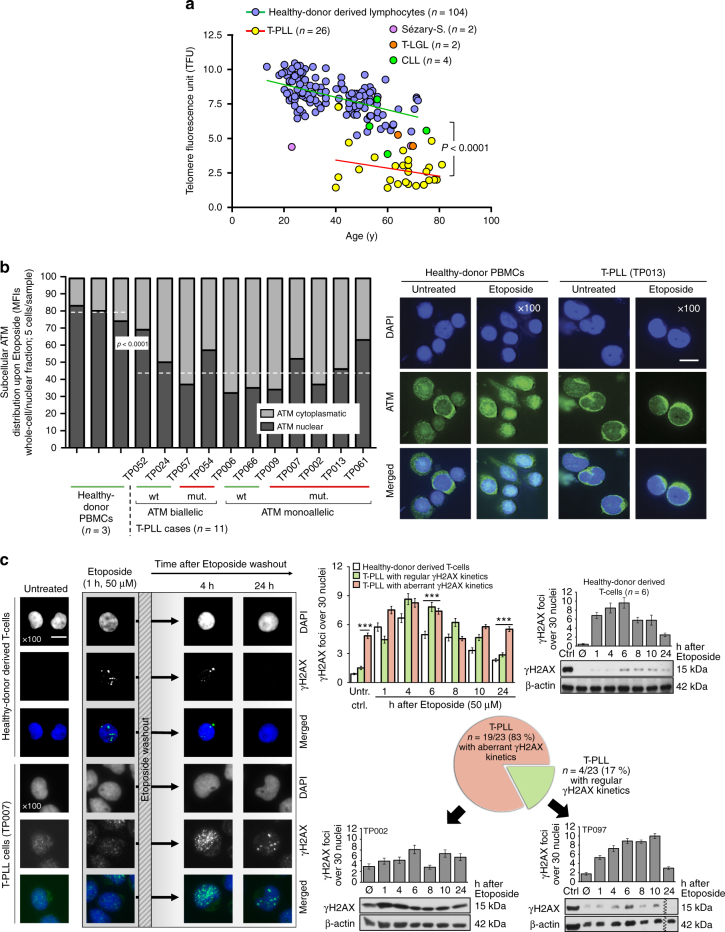
T-PLL cells show an aberrant DDR linked to hypofunctional *ATM*. **a** Reduced telomere lengths (flow-FISH, age-correlated) in T-PLL (1 telomere fluorescence unit (TFU) corresponds to 1 kb pairs; *p* < 0.0001, Student’s paired *t*-test); see also Supplementary Fig. 14a–c for WGS-based analyses and associations with *ATM* lesions. **b** Immunofluorescence (IF) microscopy. Impaired nuclear translocalization of ATM in T-PLL upon DSB induction at 1 h exposure to 50 µM Etoposide (independent of genomic *ATM* status). Left: ImageJ-quantified mean fluorescence intensities (MFIs) for nuclear signals (dashed medians). Healthy-donor PBMCs 78.6% vs. T-PLL 42.5% (*p* < 0.0001, Student’s *t*-test). Right: representative samples (scale bar = 5 µm; entire set in Supplementary Fig. 14d). **c** Aberrant kinetics of DSB-induced foci in T-PLL cells. Left: experimental outline and exemplary IF microscopy (scale bar = 5 µm). Right: summary of quantifications of yH2AX foci (30 nuclei/case; mean with SEM) in 6 samples of healthy-donor T-cells vs. 23 T-PLL with examples represented by individual focus counts over time and immunoblots (mean with SEM; Student's *t*-test, ***p < 0.0001). Abnormal (not resembling the pattern of normal T cells) formation and kinetics of yH2AX foci in 19/23 T-PLL: hardly any or delayed induction (5/19), as well as inefficient/protracted removal (14/19). In 4 cases the pattern resembled the one of normal T cells (‘regular’). The entire set of yH2AX IF and a summary of densitometries from all immunoblots are provided in Supplementary Fig. 15

The complex karyotypes of T-PLL suggest a high genome instability. Given the recurrent *ATM* lesions, we implicated abrogation of central functions of this apical master regulator of the canonical DDR as the foremost cause. In agreement with ATM’s other recognized roles in regulating ROS levels and telomere lengths, we described the elevated redox burden (Fig. 4c, Supplementary Fig. 9d; *p* = 0.005, Student's *t*-test) and demonstrated markedly shortened telomeres (*p* < 0.0001, paired Student’s *t*-test; Fig. 7a, Supplementary Fig. 14a–c); the latter corroborating a previous observation by others^44^.

We next examined the capacity of the leukemic cells to mount an adequate response to DSBs. T-PLL cells showed aberrant cytosolic retention of ATM protein upon damage induction via Etoposide (median nuclear fraction 78.6% in normal PBMCs vs. 42.5% in 11 T-PLL, *p* < 0.0001, Student’s *t*-test; Fig. 7b, Supplementary Fig. 14d). Furthermore, the kinetics of induction and resolution of induced DSB platforms marked by ATM’s target γH2AX were aberrant (different from normal T-cells) in 19 of 23 T-PLL as analyzed in time-lines of western blots and microscopic focus counts (Fig. 7c, Supplementary Fig. 15). This mainly included cases with elevated marks at baseline and a protracted resolution, but also comprised a subset (5/19) with hardly any noticeable basal and stimulated γH2AX. These data implicate impaired proximal damage sensing and processing.

Besides dysfunctional ATM, this could also be due to primary defects of γH2AX, as these are also T-cell lymphomagenic^45^ and given the monoallelic losses of the *H2FAX* locus in 22.9% (19/83) of our T-PLL. Therefore, we further recorded activation of the ATM-specific substrate and heterochromatin builder KAP1^46^ (Supplementary Fig. 16a, b). KAP1 phosphorylation in response to γ-irradiation was noted in 16/23 cases, as defined by >20% of the level of immortalized control lymphocytes from a healthy *ATM*-wt member of an *A-T* pedigree. However, 30% (7/23) of cases displayed a diminished or absent response. Lower pKAP1 responses were predominantly seen in cases with aberrant γH2AX induction (*p* = 0.0065, Mann–Whitney *U*-test; Supplementary Fig. 16c). Complete abrogation of pKAP1 induction was found in the rare T-PLL with truncating *ATM* mutations (e.g., TP053) in analogy to *ATM*^*mut/mut*^ lymphoid *A-T* cells, while most *ATM*-biallelic/wt cases were more pKAP1 responsive (Supplementary Fig. 16a–c).

Overall, we conclude that despite genomic *ATM* defects, there is preserved γH2AX / pKAP1 induction in most T-PLL, but often at sub-maximal levels or with altered kinetics of proximal platform recruitment and resolution.

### TCL1A contributes to the aberrant telomere, ROS, and DDR phenotype

**Fig. 8:**
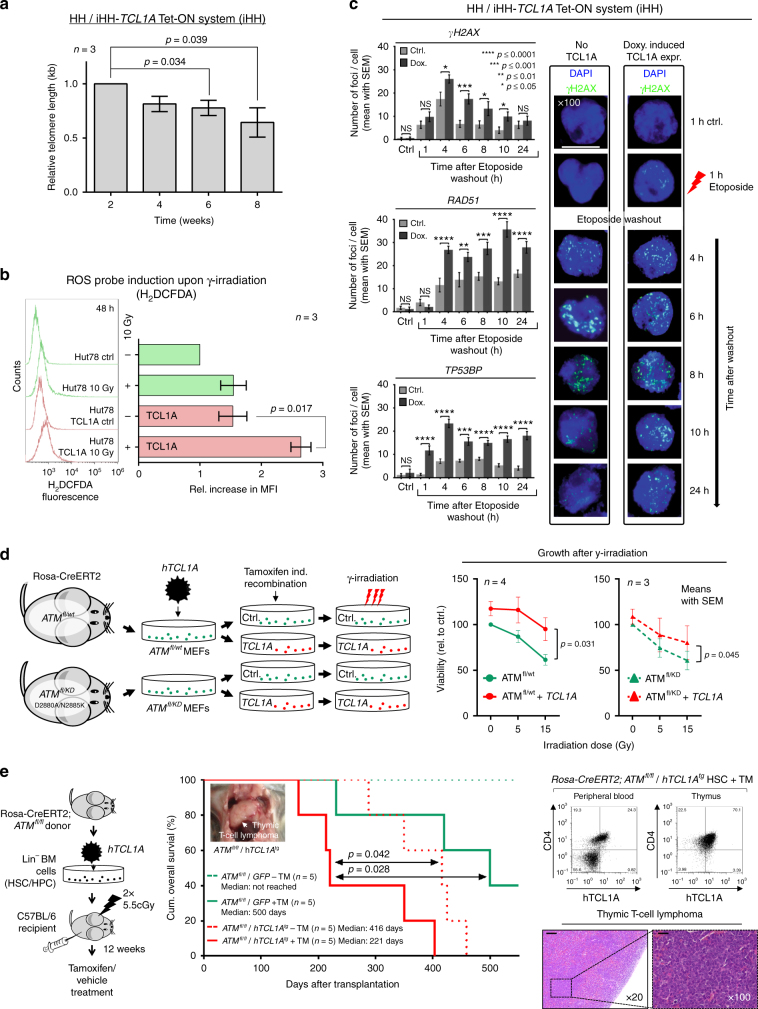
TCL1A affects ATM functions and cooperates with ATM deficiency. **a** Protracted TCL1A overexpression in HH T-cell leukemia cells mediates telomere shortening (flow-FISH; Supplementary Fig. 17b–d for controls and qRT-PCR based validations, mean with SEM; Student's *t*-test). **b** H_2_DCFDA-based measurements of ROS induction upon γ-irradiation (10 Gy) comparing parental Hut78 T-cell leukemia cells to their derivatives of stable TCL1A transfection. Left: examples of H_2_DCFDA fluorescence readout 48 h post irradiation, one representative experiment shown. Right: quantification of mean fluorescence intensity (MFI, *n* = 3 biological replicates, mean with SEM) relative to Hut78 cells without irradiation (*p* = 0.017, Student's *t*-test). **c** Enforced TCL1A expression in HH cells (doxycycline-inducible iHH) mediates higher peak focus counts of DSB marks (γH2AX, RAD51, and TP53BP1) and their impaired resolution in time-lines after washout from a preceding 1 h of Etoposide exposure. Left: quantified focus counts (mean with SEM; Student's *t*-test, ****p<0.0001, ***p<0.001, **p<0.01, *p<0.05); right: representative examples (scale bar = 7.5 µm; controls in Supplementary Fig. 17f–h). **d** Left: setup of MEFs (*Rosa-Cre*^*ERT2*^*;ATM*^*fl/wt*^ and *Rosa-Cre*^*ERT2*^*;ATM*^*fl/KD*^)^76^ stably transfected to express human (h)TCL1A. *ATM* loss was induced through Tamoxifen treatment over 3 passages resulting in *ATM*^−^^*/*wt^ and *ATM*^−^^*/*KD^ cells. Immunoblots in Supplementary Fig. 18a verify reduced ATM protein and overexpression of TCL1A. The KD-mutations D2880A/N2885K correspond to D2870A/N2875K in human *ATM*. Right: γ-irradiation reduced cell viability (proliferation) as per MTT assay (48 h) with TCL1A mediating a protective effect in scenarios of genetic *ATM* disruption (mean with SEM; Student's *t*-tests). **e** In vivo model of overexpression of *hTCL1A* and inducible *ATM*-abrogation. Left: hematopoetic stem cells (HSCs) of *Rosa-Cre*^*ERT2*^*;ATM*^*fl/fl*^ mice were retrovirally transduced with *hTCL1A* or a GFP control vector and transplanted into irradiated syngeneic hosts. Recombination of the *ATM* locus (fl/fl) was induced by Tamoxifen (TM) injections starting 12 weeks after transplantation (1 mg/day i.p. for 5 consecutive days; further details in Supplementary Fig. 18c, d and Methods section). Middle: Kaplan–Meier curve showing accelerated T-cell lymphoma/leukemia onset and shorter animal survival of the *ATM*^*fl/fl*^/*hTCL1A*^*tg*^ genotype (log-rank tests, time from transplantation to event). Right: evidence of hTCL1A-protein positive T-cells (flow cytometry) in blood and thymus involved by a CD4^+^/8^+^ T-cell tumor (*ATM*^*fl/fl*^*/hTCL1A*^*tg*^). H/E stains (scale bar left = 100 µm; right = 400 µm) of one exemplary thymic T-cell lymphoma (*ATM*^*fl/fl*^*/hTCL1A*^*tg*^)

In contrast to *ATM*^null^ *A-T* cells, the *ATM* lesions in T-PLL are obviously not associated with elevated chemo-/radiosensitivity. This might be linked to the residual functions of the mutated *ATM* (above, Fig. 7c, Supplementary Figs. 15b,16) and could also involve a rescue relationship with anti-apoptotic TCL1A. Consequently, we modeled the specific impact of TCL1A after introduction into the *ATM*-biallelic mature T-cell leukemia lines HH (inducible; Supplementary Fig. 17a) and Hut78 (stable). In resemblance of the phenotype of T-PLL cells, TCL1A propagated telomere shortening (*p* = 0.039 at 8 weeks, Student’s *t*-test; Fig. 8a, Supplementary Fig. 17b–d) and higher γ-irradiation-induced ROS levels (*p* = 0.017, Student’s *t*-test, Fig. 8b). This was likely not attributed to replicative stress, as there was no pro-proliferative effect by TCL1A (Supplementary Fig. 17e). Furthermore, in the presence of TCL1A, the extent of induced DSBs was increased and their processing was markedly protracted, as per kinetics of γH2AX, RAD51, and TP53BP1 foci and γH2AX expression levels (Fig. 8c, Supplementary Fig. 17f–h).

We next interrogated scenarios of deficient *ATM*. For that, we introduced *TCL1A* into immortalized murine embryonic fibroblasts (MEFs) carrying transgenes for the conditional expression of the *ATM* variants *ATM*^*fl/wt*^ (monoallelic loss) or *ATM*^*fl/KD*^ (PI3-kinase dead (KD) mutation)^43^, mimicking the lesions identified in T-PLL. We observed protective effects of *TCL1A* expression in response to γ-irradiation in both lines of *ATM* disruption (*p* = 0.031 (*ATM*^*fl/wt*^), *p* = 0.045 (*ATM*^*fl/KD*^), Student's *t*-tests; Fig. 8d, Supplementary Fig. 18a). Similarly, TCL1A mitigated the irradiation-induced reductions in Hut78 T-cell viability (*p* = 0.034), particularly in the context of inhibition of ATM activity by KU-55933 (*p* = 0.026, paired Student's *t*-tests; Supplementary Fig. 18b).

### A pro-leukemogenic cooperation of TCL1 with ATM deficiency

We asked whether there are pro-tumorigenic synergisms between *TCL1A* overexpression and genomic *ATM* loss in mice. A design of *TCL1A* overexpression initiated in hematopoetic precursors followed by induced ATM depletion after engraftment in syngeneic hosts allowed assessments for lineage biases and error-prone DSBs during physiological *V(D)J* gene rearrangements to take place. Induction of thymic T-cell lymphomas/leukemias, which were more aggressive in *ATM*^*−l*^^*−*^*/hTCL1A*^*tg*^ animals (median survival 221 days) than those induced by the single-hit genotypes (*ATM*^*−/−*^*/GFP*^*+*^, 500 days, *p* = 0.028; *ATM*^*fl/fl*^*/hTCL1A*^*tg*^, 416 days, *p* = 0.042, log-rank tests; Fig. 8e, Supplementary Figs. 18c,d), provided affirmation of a TCL1/ATM cooperation.

### Pharmacologic targeting of the dysfunctional ATM/p53 axis

**Fig. 9:**
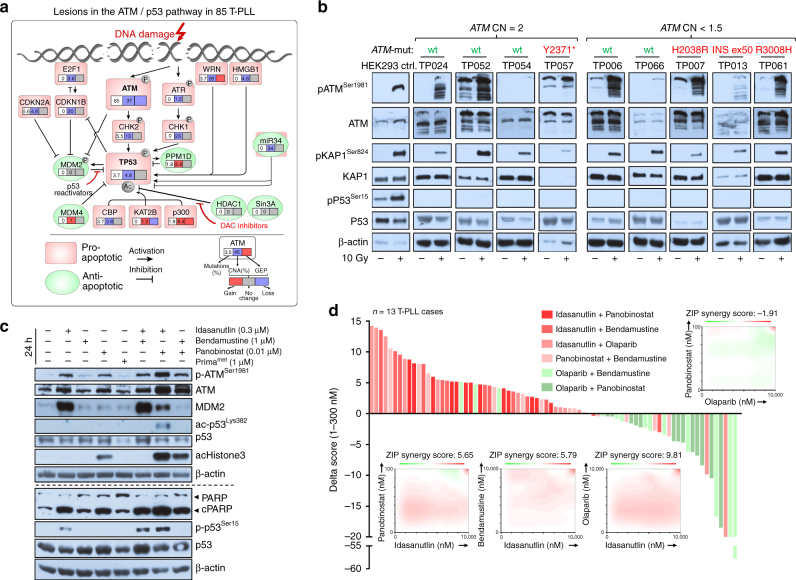
Pharmacologic exploitation of the defective ATM/p53 axis. **a** Summary of genomic lesions (sCNAs, mutations) and gene expression changes affecting *TP53* and its direct regulatory network detected in this series of T-PLL. **b** ATM^Ser1981^, KAP1^Ser824^, and p53^Ser15^ phosphorylation upon 10 Gy ionizing irradiation in 9 T-PLL; controls: ATM/P53-competent HEK293 cells. Robust pKAP1 induction (e.g., TP052; ATM-wt/CN = 2), as well as reduced activation is observed (e.g., TP007; ATM-mut/CN = 1.5). Despite at least weak pATM/pKAP induction for most cases, none showed a pP53 response (irrespective of genomic *ATM* status). All cases lacked 17p sCNAs and *TP53* mutations. Median purity: 97.5% T cells; lanes separated for genotype-based arrangement (* denotes nonsense mutation). For results on CHEK2 phosphorylation in a subset of cases see Supplementary Fig. 19a. **c** Idasanutlin (0.3 µM) reinstated phospho- and acetyl-marks of p53 activity in T-PLL cells. Co-treatment with Bendamustine (1 µM) or Panobinostat (0.01 µM) further enhanced this, including the apoptotic response (cleaved PARP). Immunoblot of case TP104 (representative of 3) at 24 h. There was no p53 phospho-activation by Prima-1^met^, a selective reactivator of mutated p53 (Supplementary Fig. 19b). **d** In vitro screen for interactions of Idasanutlin (I), Panobinostat (P), Bendamustine (B), and Olaparib (O) across 13 T-PLL. Each of the 6 combinations was plated in a matrix of 7 concentrations covering a 1000-fold range. Incubation of samples in duplicates for 72 h and cell viability as per luminescent CellTiter-Glo assay; positive control: 100 μM Benzethonium-chloride; negative control: DMSO. A delta score reflects positive (synergistic) and negative (antagonistic) drug interactions. It is calculated based on a zero-interaction potency (ZIP) reference model and is computed using the synergyfinder R-package (details in Online Supplements). Waterfall plot: ranking of each combination based on the delta scores of the low dose ranges (1–300 nM) across all samples. Rank-sums over 13 cases significantly differed among combinations (I + P:63, I + B:60, I + O:56, P + B:51, O + B:24, O + P:19; *p* < 0.0001, one-way Friedman Anova, highest ranks for highest score values). Combinations of I + P (*p* = 0.015) and I + B (*p* = 0.04) were superior to P + B (Wilcoxon rank-sum test). O + B or O + P showed antagonistic relationships in most T-PLL. Four selected 2D plots illustrate the distribution of synergistic (red) or antagonistic (green) interactions over the entire dose range of all 13 cases (positive controls in red in upper right corners)

Pro-apoptotic signaling in response to most kinds of DNA damage is centrally relayed through activation of p53. In our cohort of T-PLL, sCNAs (4.8%, 4/83 cases) and mutations (3.7%, 2/54 cases) that disrupt *TP53* were rare. P53 regulators were also very infrequently affected (Fig. 9a). Given the preference for *ATM* lesions and the outlined phenotype of partial loss of ATM function (above), we tested whether p53 could still be activated via the ATM/CHEK2 route. Upon γ-irradiation, irrespective of any pATM^S1981^ induction (retained in 8 of 9 cases), T-PLL cells failed to generate a pP53 response in all 9 analyzed samples (Fig. 9b; accompanied by lack of pCHEK2 induction in 4/6 cases, Supplementary Fig. 19a). This deficient upstream activation would implicate that the apparently genetically intact, hence functional, p53 is retained in its inactive state.

Such a tonus of p53 inactivity is usually mediated by higher net binding to its repressor MDM2 and deacetylation of its functional domains. Consequently, in a proof-of-principle approach we interrogated T-PLL cells for vulnerabilities around their ATM/p53 incompetence. We applied the MDM2-inhibitor Idasanutlin as a pharmacologic backbone of p53 de-repression and investigated co-operations with selected other classes of compounds. Several notions prompted us to additionally target chromatin remodeling and protein deacetylation by a histone deacetylase inhibitor (HDACi; Panobinostat): (1) our profiling data identify various recurrent lesions in DNA repair molecules and histone modifiers (Figs. 4, 6, Supplementary Data 12; 91.7%), (2) DNA-repair viably depends on histone modifications^46^, (3) sufficient activation of p53 and ATM involves their direct HAT mediated acetylation^47,48^, (4) HDACi’s show a particularly high activity in T-cell tumors and can revert resistance in T-PLL^49^. For additional DSB induction we opted for the nucleoside-like alkylator Bendamustine, because of its clinical activity in T-PLL^50^. A fourth class of substances, PARP-inhibitors (here Olaparib), was chosen based on the well-established synthetic lethal relationship with defective ATM^51^.

Idasanutlin at sub-LD50 concentrations reinstated repressed phospho- and acetyl-marks of p53 activity in T-PLL cells (Fig. 9c, Supplementary Fig. 19b). This was enhanced by co-treatment with sub-LD50 dosages of Panobinostat or Bendamustine. As single agents, Idasanutlin and Panobinostat profoundly and selectively induced apoptosis in T-PLL cells with LD50s in the nM range (Supplementary Fig. 19c). In a combinatorial matrix of the 4 substances (each at 7 concentrations over a 10^3^-fold range) we then interrogated for over-additive interactions affecting cell viability in 13 T-PLL (Fig. 9d). Synergisms in low-mid nM-ranges were identified for all Idasanutlin combinations, which were particularly active when involving the HDACi Panobinostat. Finally, in a pilot experiment of mice transplanted with leukemic *CD2*-*MTCP1*^*p13*^ cells, Idasanutlin reduced T-PLL burden in blood (*p* = 0.048) and spleens (*p* = 0.003, Student's *t*-tests; Supplementary Fig. 19d).

## Discussion

Unraveling the molecular liabilities of T-PLL would be seminal for therapeutic advancements in this devastating disease. In a large T-PLL cohort we integrated multi-level genetic alterations and delineated from mechanistic models that constitutive activation of TCL1A cooperates with dysfunctional ATM to drive transformation. This unique lesional partnership is not observed in other T-cell lymphomas^52,53^.

Virtually every case (94.6%) fulfilling the WHO classification criteria for T-PLL^1,52^, demonstrated here a genomic rearrangement involving a *TCL1* gene and/or its abnormal expression. TCL1A can enhance signals from the most central growth receptor of T-cells, the TCR^5^. As suggested here also from TCL1A-initiated murine T-PLL, this primary step toward perturbation of a protective T-cell homeostasis^54^ likely entails additional downregulation of negative TCR regulators (e.g., *CTLA4, SLAMF6*), resulting in augmented TCR net activation. Fittingly, there were no recurrent mutations in TCR pathway genes, unlike in nodal mature T-cell lymphomas^55,56^.

As a phenotypic hallmark of T-PLL, we identified a pronounced genomic instability, demonstrated by complex losses and gains and novel molecular hybrids. The global signature of nucleotide exchanges indicated cumulative genotoxic insults in a repair deficient precursor cell. *ATM* was the gene most recurrently affected (86%, Fig. 6) by allele deletions (37%) and/or clonally dominant mutations (65%). The previously undisclosed clustering of the mostly missense mutations in *ATM*’s FAT and PI3K-domains fits well with the profile of *ATM* variants from >5400 cancer genomes^43^.

It has been shown that *ATM* PI3K-domain dead (KD) alleles are more oncogenic than the *ATM*^null^ scenario^43,57^. Furthermore, the ATM kinase domain seems dispensable for recruitment to damage sites^43,57^. This is in agreement with the residual, albeit diminished, damage sensing, and platform recruitment, observed here. It is therefore attractive to speculate that the expressed mutated ATM in T-PLL is hypomorphic only with respect to selective ATM functions. In fact, a robust activation of the heterochromatin factor KAP1 was frequently seen (Supplementary Fig. 16a, b) and may represent a GOF branch of altered ATM, in resemblance of oncogenic empowerments of mutated p53^58^. In indirect support of this theory is the assumption that an *ATM*^*null*^ constellation is likely disadvantageous given the reported absence of *ATM* promoter silencing in T-PLL^59^ and a complete lack of cases with biallelic *ATM* losses here (Fig. 5b).

Obviously, major ATM/p53-mediated branches of the DDR to restore genome integrity or to execute safeguarding responses, e.g., to oncogenic stressors or therapy, are insufficient in T-PLL. This might also be due to incomplete compensation by stand-in’s (i.e., ATR) or supplementing defects of other DNA repair genes, as we detected this in the few cases with unaffected *ATM*. Importantly, we provide first hints that consequences of functional ATM deficiencies (e.g., in regulated redox homeostasis or maintenance of telomere length^18,32^) are aggravated by the pro-survival or other specific effects of TCL1 (Figs. 7, 8). In support, we previously showed TCL1A to augment mitochondrial ROS biogenesis^60^. As full ATM incompetence per se is pro-apoptotic, the coinciding impact of TCL1 likely perturbs such protective programs making this a potent leukemogenic liaison. In fact, TCL1A can rescue the apoptotic phenotype of *A-T* cells while potentiating their chromosome fragility^15,61^.

Among the cataloged aberrations, the most recurrently affected functional branch was the DDR. However, at the regulatory level the category of epigenetics, predominantly defined by histone modifying molecules (*EZH2*, *KMT*s, and *HDAC*s) was most frequently involved (Fig. 6). Intriguingly, chromatin modulation is an increasingly recognized determinant of proper DSB processing and dictates treatment resistance^46,47,49^.

The spectrum of other pivotal alterations included amplified programs of *MYC* or miR-based dysregulations (e.g., *AGO* genes, *MIR34* cluster). Subclonal mutations in *JAK/STAT* genes were detected in 61.6%, a frequency that is in agreement with recent reports^21–24,42,62^. Nevertheless, their differential GOF effects need more thorough alignments with inhibitor sensitivities^62^.

A central mechanistic finding was the inability of T-PLL cells to mount a DSB-induced activation of p53. This can explain the clinical chemo-refractory behavior of T-PLL. In contrast to many cancers^63^, the incidence of p53 disrupting genomic lesions in T-PLL is surprisingly low. Our data implicate that in T-PLL mainly dysfunctional proximal ATM renders p53 inactive. Since ATM kinase activity and p53 de-repression stand in a synthetic lethal relationship^64^ and in light of the need for non-conventional therapies in T-PLL, we devised here a promising interventional strategy. P53 reactivation by MDM2-interference was highly synergistic with DAC inhibition of histones and central relay proteins, including ATM and p53.

**Fig. 10:**
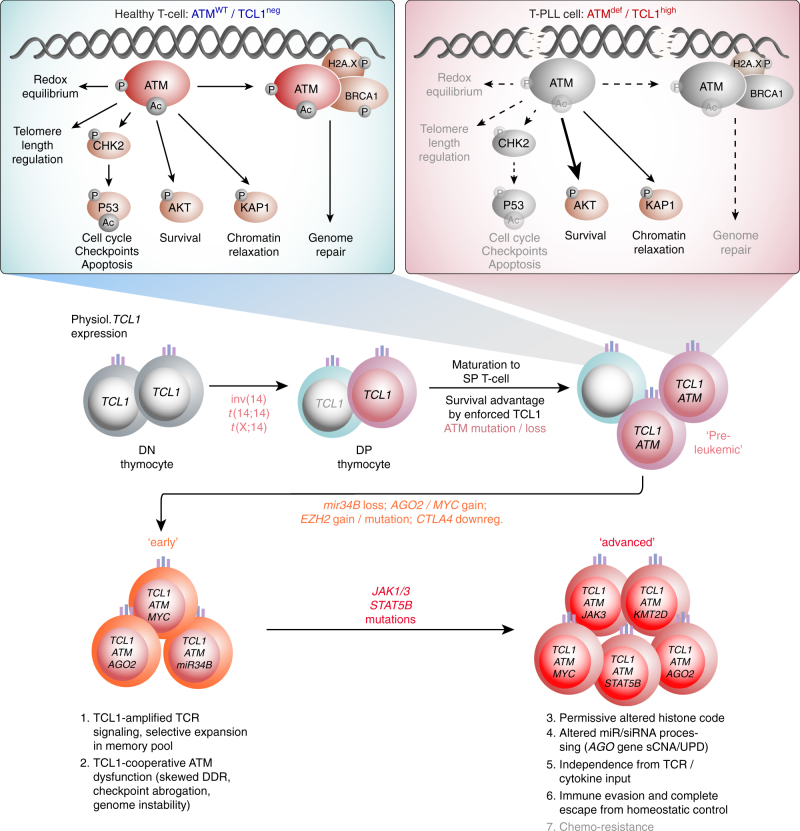
Proposed model of T-PLL development. Top: Projections from an ‘unaffected’ thymic emigrant (left) compared to the scenario of a post-thymic T-PLL precursor (right box) with inappropriate expression of TCL1 and deficient ATM. Effects of this postulated initiating core lesion of *TCL1*^up^/*ATM*^def^ on the key signaling branches and functions of ATM are highlighted by differential arrows. It includes perturbation of some of ATM’s safeguarding tasks by TCL1 resulting in cell-death evasion. The ‘TCL1’-lesion refers to the deregulation of any TCL1 family member. *TCL1*^up^/*ATM*^def^ jointly confer a functional signature of ATM to be inefficient in counteracting oxidative damage, to maintain telomere and genome integrity, and to activate protective p53 programs. Timeline: Chronology assumptions are based on identified frequencies in sCNA data and tumor fractions in sequencing data. Constitutive TCL1 expression and loss-of-negative regulators (e.g., CTLA4) cause amplifications of TCR-derived survival signals. In the context of dysfunctional ATM this entertains ROS accumulation and genomic instability, which in turn entails further alterations of oncogenes like *MYC*, of epigenetic modifiers, and of miR processing (e.g., AGO2). Overt-stage autonomous proliferation, including escape from niche-defined homeostatic control relies on independence from milieu input, as potentially conveyed by *JAK/STAT* mutations. Emerging data, e.g., on the impact of JAK/STAT signaling on non-canonical functions of histone modulators like EZH2^77^ indicate yet unrecognized cross-talks between the affected functional branches

Overall, the presented molecular profiles and novel functional insights allowed the formulation of a first integrative model of T-PLL leukemogenesis (Fig. 10). However, this has to be expanded on at various levels. Also, the T-cell permissive cooperation between TCL1 and ATM needs to be further interrogated for the concise pathway overlaps and the exact modes of checkpoint abrogations through TCL1. This also has to consider the significance of a protein–protein interaction of TCL1 with ATM^65^.

## Methods

### Patient samples

Primary T-PLL cells were isolated from peripheral blood (PB) of 111 T-PLL patients diagnosed according to WHO criteria^1,52^. Differential diagnosis was based on clinical features, immunophenotyping (flow-cytometry and histochemistry; including TCL1A/MTCP1 expression), FISH/karyotypes, and molecular studies (e.g., *TCR*-monoclonality). Human tumor samples were obtained from patients under IRB-approved protocols following written informed consent according to the Declaration of Helsinki. Collection and use have been approved for research purposes by the ethics committee of the University Hospital of Cologne (#11–319). The cohort was selected based on uniform front-line treatment (87% of cases) with either single-agent alemtuzumab or fludarabine-mitoxantrone-cyclophosphamide (FMC) plus alemtuzumab chemo-immunotherapy (similar efficacy, refs. ^3,4,66^) as part of the TPLL1^4^ (NCT00278213) and TPLL2 (NCT01186640, unpublished) prospective clinical trials or as included in the nation-wide T-PLL registry (IRB# 12-146) of the German CLL Study Group (GCLLSG; Supplementary Data 1).

Patients had a median age of 66 years at diagnosis and included 1.5-times more men than women. Overall survival (OS) was measured as the time from diagnosis to disease-specific event or censoring. Kaplan–Meier curves were compiled with PRISM6; with log-rank statistics for 2-group comparisons.

A small number of samples from other entities was included as references: T-cell large granular lymphocytic leukemia (T-LGL, *n* = 2) for WES and telomere length assessments, as well as Sézary syndrome (SS, *n* = 2) and chronic lymphocytic leukemia (CLL, *n* = 4) for telomere length assessments.

### Flow cytometry

Flow cytometry was performed on a Gallios (BeckmanCoulter) cytometer, using antibodies against human CD3 (clone Hit3a), CD4 (clone OKT4), CD5 (clone UCHT2), CD7 (clone 6B7), CD8 (Hit8a), CD45 (clone 2D1), and TCL1A (clone 1–21^67^), all from BioLegend. Intracellular staining was performed according to the manufacturer’s instructions using the IntraPrep kit (BeckmanCoulter). We observed CD4 single positivity in 58%, CD8 single positivity in 16%, and CD4/CD8 double positivity in 24% of cases.

### Magnetic-bead based cell enrichment

Peripheral blood mononuclear cells (PBMCs) of T-PLL patients or healthy volunteers were obtained by density gradient centrifugation (Histopaque, Sigma-Aldrich). DNAs of matched tumor/germline (t/g)-pairs were obtained after magnetic-assisted cell sorting (MACS), separating CD4^+^ or CD8^+^ T-PLL cells from non-tumor hematopoietic cells with a final purity of >98% (Supplementary Fig. 1b). We conceptualized this T-cell enrichment to involve a sequential 2-step separation process of which each was carried out according to the manufacturer’s (Miltenyi Biotec) instructions: (1) positive enrichment of T-PLL tumor cells followed by (2) depletion of residual T-PLL cells from the flow-through obtained from step 1 to recover a pure non-tumor cell fraction. According to the predominant immunophenotype, samples were first enriched for CD4^+^ (#130-045-101, Miltenyi Biotec) or CD8^+^ (#130-045-201, Miltenyi Biotec) lymphocytes using microbeads of the MACS system (Miltenyi Biotec) and LS Columns (#130-042-401, Miltenyi Biotec). (2) For depletion of the normal control fractions (neutrophils, monocytes, NK-cells, B-cells) by contaminating T-PLL cells, LD Depletion Columns (#130-042-901, Miltenyi Biotec) were used to remove residual CD4^+^ or CD8^+^ cells from the flow-through obtained from step 1. Purity of cell populations was assessed by flow cytometry. PBMCs of healthy volunteers were enriched for CD3^+^ pan T-cells (regular CD4^+^/8^+^ ratio of 1.5–2.5) or CD45RO^+^ pan memory T-cells using MACS beads (130-050-101 (CD3+) and 130-091-893 (CD45RO+), Miltenyi Biotec).

### Cell cycle analysis

Flow-cytometry based cell cycle analysis was performed according to standard protocols. Briefly, cells were collected, vortexed intensely in Nicoletti buffer (0.1% w/v Sodium citrate, 0.1% v/v Triton X-100, 50 µg/ml propidium iodide freshly added) and incorporation was measured.

### Murine models for T-PLL and in vivo compound efficacy testing

We re-derived the originally described hemizygous *Lck*^*pr*^*-hTCL1A*^*+/-*^ transgenic (tg) mice^10^ from frozen sperm straws (JAX mice research, The Jackson Laboratory) by egg fertilization and embryo transfer. They represent an autochthonous model for human T-PLL^68^. Following the early (thymic) onset of constitutive expression of human TCL1A, according to the activity of the proximal *Lck* promoter, the animals develop a CD8^+^ disease that resembles human T-PLL^68^.

To test drug efficacies (e.g., Idasanutlin) in vivo, transplantable leukemias/lymphomas derived from *CD2-MTCP1*^*p13*^ *tg* mice^11^ (predominantly blood, spleen, bone marrow) were i.p. injected into background-matched recipients to facilitate the generation of uniform cohorts, which is not possible in the original systems due to long latencies and their wider ranges (despite 100% penetrance) of clinical disease onset. The *CD2-MTCP1*^*p13*^ tg system is a T-PLL model analogous to TCL1A-tg, but transplantable lines with slow and fast (latter chosen here) growth kinetics were only established for the *CD2-MTCP1*^*p13*^ model at the time of study. 1 × 10^7^ cells were i.p. injected into syngeneic recipients (*n* = 16). Starting on day 11 post transplantation for 5 consecutive days, mice were treated with vehicle control, or Fludarabine (35 mg/kg days), or Idasanutlin (35 mg/kg). Animals were randomly assigned to treatment groups (homogeneous distribution as per leukocyte counts).

In order to test the in vivo pro-leukemogenic cooperation of *ATM* loss with *TCL1A* overexpression, hematopoietic stem cells (HSCs) from *Rosa26-CreERT2;ATM*^*fl/fl*^ mice^69^ were isolated from bone-marrows and retrovirally transduced in vitro with an expression vector for human *TCL1A* or *GFP*. Transduced HSCs were re-transplanted into sub-lethally irradiated background-matched 8-week-old recipients and Tamoxifen at 1 mg/day was i.p. injected for 5 consecutive days 12 weeks after transplantation to generate *ATM* deficiency from the recombined *ATM*^*fl/fl*^ alleles.

Murine formalin-fixed and paraffin embedded tissue material (FFPE) was stained with hematoxylin and eosin (H/E).

All experiments involving living animals were conducted according to the German Animal Welfare Act (approval numbers: 20.12.A166 (*Lck*^*pr*^*-hTCL1A* mice; LANUV, NRW, Germany), 2012.A394 (in vivo treatment of *CD2-MTCP1*^*p13*^; LANUV, NRW, Germany), F21/03_RP_Darmstadt (transplantation of sub-lethally irradiated mice with genetically modified HSCs; MUKLV, HE, Germany). The animal experiments served as proof-of-principle/pilot studies, hence, minimal sample sizes of *n* = 5 were considered sufficient.

### Gene expression profiling (GEP)

GEP of human T-PLL cells: For sample preparation, PBMCs were isolated from T-PLL patients (>95% purity of T-cells) and CD3^+^ T-cells isolated from PB of healthy donors (see paragraph “Magnetic-bead based cell enrichment” for detailed descriptions on cell purification) were submitted to RNA isolation using the mirVana kit (Invitrogen). GEP analyses were conducted using Illumina HumanHT-12 v4 BeadChip arrays according to manufacturer’s instructions.

Bioinformatics: We used the Illumina proprietary software GenomeStudio v1 to background-correct and to initially annotate the probes of the HumanHT-12 v4 Expression BeadChip. We filtered samples and genes by detection *p*-values and fluorescence intensities for at least 2/3 hits (*p* < 0.05) to reduce false calls. Batch-effects were corrected by the ComBat method which uses an empiric Bayesian model framework. For literature references to bioinformatics methods and algorithms see Supplementary Table 2. Since the official Illumina HumanHT-12 v4 Expression BeadChip annotation is outdated, we used the data mining tool biomaRt, version 75 of the Ensembl data base with R, version 3.1.0, and Bioconductor, version 2.10.

T-PLLs (*n* = 70) and normal controls (CD3^+^ T-cells from 10 healthy donors) were grouped and tested separately for differential expression using the Student’s *t*-test on log-transformed fluorescence values (normally distributed). Fold-changes (fc) were calculated on the fluorescence values without logarithmic transformation. False discovery rates (FDRs) were calculated using the R package “q-value”. Hierarchical clustering was carried out using the R package gplots, version 2.15.0 (distance function: euclidean; clustering: complete linkage). In Fig. 1a, the dendrogram was manually cut to obtain clusters with unique expression patterns. Gene expression overlaps between human and mouse were evaluated using Venny. Functional analyses of (differentially expressed) genes was carried out by Ingenuity Pathway Analysis (IPA, http://www.ingenuity.com/products/ipa), ConsensusPathDB (GSOA), Broad GSEA 2–2.2.1, and KEGG/GO enrichment from the R package STRINGdb, version 9_05.

For identification of prognostic GEP signatures, GEPs of T-PLL cases with longest (>800 days, 5 cases) overall survival (OS; time from diagnosis to death of disease; no other events included) were compared with GEPs of cases with shortest OS (<300 days, *n* = 5) using “Significance analysis of microarrays” (SAM) analysis in survival mode^70^ (first training set of 10 cases). We only considered cases in which the sampling date was no longer than 6 months from diagnosis and with similar lymphocyte doubling times (LDTs) at presentation. From an initial most informative index-set of 5 differentially expressed probes (*RAB25*, *KIAA1211L-probe1*, *KIAA1211L-probe2*,* GIMAP6*, *FXYD2;* FDR < 0.1), linear regression (and one outlier removal by setting OS < 200 days, second training set of 9 cases), followed again by SAM analysis (survival mode), resulted in a 2-gene/3-probe set (*ILMN_1791826* mapping to 4 transcripts including *RAB25-001/ENST00000361084* responsible for standard protein *ENSP00000354376*; *ILMN_1776121* and *ILMN_3243366* both mapping to *KIAA1211L-001/ENST00000397899* responsible for standard protein *ENSP00000380996*; no other probes mapping to both genes) as the most robust predictors (only when combined). Their probe sets were used to calculate an expression index (via additive model fit using Tukey’s median polish procedure) on the test set of uniformly treated 40 cases of the GEP-analyzed T-PLL cohort (9 cases of training set (above) excluded) fulfilling the respective criteria (available GEP and OS data). Kaplan–Meier curves (log-rank tests for differences) were created based on stratified values per patient of this “2-gene/3-probe prognostic expression index”. Ranking the cases solely based on these expression indices, the 5 T-PLL with the lowest values indeed showed significantly superior OS (only 2 of these 5 cases received an allogenic stem cell transplantation) over those with higher expression index values (index fc = −1.62; Supplementary Fig. 2e).

GEP in murine T-cell leukemia: For sample preparation, murine spleens removed post-mortem were mashed through a 100 μm cell strainer (BD Biosciences) and lymphoid cells were isolated using density gradient centrifugation. Cells were subsequently enriched for CD8^+^ lymphocytes using MACS beads (130-049-401,Miltenyi Biotec). RNA was isolated from murine tissues using the mirVana kit (Invitrogen). We hybridized 3 control RNA samples (pooled from CD3^+^ T-cells enriched from 9 spleens of age- and background-matched wt animals), as well as RNA isolated from CD8^+^-enriched splenic T-cells of 3 “chronic phase” and of 5 “exponential phase” *Lck*^*pr*^*-hTCL1A*^*+/−*^ mouse lymphoma samples on Affymetrix Mouse Gene 1.0 ST Arrays. Definition of stages: “chronic”—30–70% tumor cells in PB and spleen, average age 12 months; “exponential”—mean PB lymphocyte doubling time (LDT) 12 days (SEM 0.8), >80% tumor cells in PB, >90% in spleen, average animal age 15 months.

Bioinformatics Arrays were pre-processed, background-corrected (RMA), quantile-normalized, and separately analyzed (chronic phase vs. ctrl., exponential phase vs. ctrl.) with the “affy” R-package. Annotation of mouse probe sets and human orthologues was carried out with biomaRt. We did not only overlap Ensembl IDs, but converted MGI gene names and overlapped them with official gene symbols as well.

### Somatic copy-number alterations (sCNAs)

Human T-PLL cells: For sample preparation, DNAs were isolated from PBMCs of T-PLL patients (*n* = 83, >95% purity of T-cells) that included 13 CD4/CD8-enriched/depleted tumor/germline (t/g) pairs (see chapter “Magnetic-bead based cell enrichment” for details on cell purification) using the QIAamp DNA Kit (Qiagen). SNP-array analyses were conducted using Affymetrix SNP 6.0 chips according to manufacturer’s instructions.

Bioinformatics: To globally infer on sCNAs across the T-PLL genome, the T-PLL data sets were compared to the pooled controls (non-tumor hematopoietic cell DNA as ‘germline’ from T-PLL patients, *n* = 13) obtained by the Affymetrix Power Tools, version 1.14.2 with duplicate SNP/CN markers (by identical position) removed. We segmented the called SNP/copy number (CN) markers by the Circular binary segmentation (CBS) algorithm (default options, *p* < 0.01) within the DNAcopy R-package and converted the output files to .seg files to view them in the “Integrative Genome Viewer”. Since the CBS algorithm only reports significantly altered segments/regions and therefore disregards gene structure (perhaps splits them in two or more segments), we mapped regions on gene CDS (based on version 75 of the Ensembl annotation) within the GenomicRanges R package, version 1.16.4, and clustered CNs by gene names and 100 kb regions with the gplots R package. We calculated the frequency by which samples surpassed CN thresholds (CN < 1.5 for losses, CN > 2.5 for gains) enabling the identification of the minimal (common) deleted or amplified regions (MDRs/MARs) and their prevalence across the T-PLL cohort. Hot spots of sCNAs were identified by visual inspection, by genes (CDS ranges) assigned to segments called by the CBS algorithm as well as by confirmatory GISTIC2.0 analyses (with removal of centromeric and telomeric regions with options: —smallmem 1 —broad 1 —brlen 0.9 8—conf 0.99 —armpeel 1 —qvt 0.05).

Loss-of-heterozygosity (LOH): To evaluate CNNLOH (copy-number neutral LOH)/UPD (uniparental disomy), we focused on those genes that show LOH and are in a biallelic state (CN between 1.9 and 2.1). We obtained genotypes from the SNP array data using Affymetrix Power Tools, version 1.14.2, and the Birdseed algorithm, and mapped specific SNPs to the genes by version 75 of the Ensembl annotation.

Meta-analysis A meta-comparison of published data on neoplasms hybridized to Affymetrix GenomeWide SNP 6.0 arrays available at GEO was performed to compare the spectrum of sCNAs with the one of our T-PLL data set (references in Supplementary Table 2). The HapMap data set “GenomeWideSNP_6.hapmap270.na32.r1.a5.ref” obtained from the Affymetrix support site served as a reference. Each sample was analyzed via CBS and those with significant gains or losses (CN > 2.5 or CN < 1.5) were selected. We grouped these segments into region size bins for each sample, i.e., one for segments of size from 1 to 1000 bp, one for 1001 to 10,000 bp, and so on. This enabled comparisons between the CN spectra across experiments and entities.

Murine T-cell leukemia: We hybridized DNA samples (QIAamp DNA Kit, Qiagen) onto the Affymetrix MOUSEDIVm520650 chip. We compared 4 controls (DNA isolated from normal liver tissues of age- and background-matched wild-type mice) to 3 ‘chronic phase’ and 5 ‘exponential phase’ (defining features in "GEP in murine T-cell leukemia") splenic isolates from T-cell leukemia / lymphoma bearing *Lck*^*pr*^*-hTCL1A*^*+/-*^ mice.

Bioinformatics Arrays were pre-processed and separately analyzed (‘chronic phase’ vs. ctrl., ‘exponential phase’ vs. ctrl.) with the ‘mouseDivGeno’ R-package.

### Whole-exome sequencing (WES)

Sample preparation: DNAs were isolated from CD4 or CD8 enriched tumor/germline (t/g)-pairs (*n* = 13) and from CD45RO+ enriched memory T-cells from healthy donors (see “Magnetic-bead based cell enrichment” for details on cell purification) using the QIAamp DNA Kit (Qiagen). Exomes were prepared by fragmenting 1 μg of DNA using sonication technology (Bioruptor, Diagenode, Liège, Belgium) followed by end repair and adapter ligation including incorporation of Illumina TruSeq index barcodes. After size selection and quantification, pools of 5 libraries were each subjected to enrichment using the SeqCap EZ v2 Library kit from NimbleGen and following the NimbleGen SeqCap EZ Library SR User’s Guide version 3.0 protocol^71^.

Bioinformatics: We sequenced 17 T-PLL (t/g)-pairs and 37 T-PLL tumor-single samples (with F/U samples on 5 of them). To establish the ‘T-cell-aging’ -associated mutational signatures, we analyzed memory T-cells isolated from 3 age-matched healthy donors (ages 61, 63, and 65 years; median for the T-PLL cases was 66 years) as well as memory T-cells from 3 young healthy donors (ages 22, 28, and 31 years). To exclude most inter-individual variations, WES profiles of each “old” memory T-cell samples were compared to each “young” memory T-cell sample separately and only recurring SNVs (3/3) that passed the ‘pseudosomatic’ filters (see above) were kept. Median numbers of G > T mutations in affected patients (*n* = 15/17) and median numbers of C > A mutations in affected patients (*n* = 12/17) were compared to all remaining substitutions and those numbers found in memory T-cell comparisons (Fig. 4c) with Odds ratios and Fisher’s count test.

We used the Illumina HiSeq2000 at the Cologne Center for Genomics (CCG), except for 8 t/g-pairs and 8-tumor singles that were analyzed at another facility (University of Michigan, collaborator/co-author K.E.-J.) for evaluations of data robustness. The cumulative exonic region (based on Ensembl 71 annotation) with at least 30× average depth coverages (calculated with GATK DepthOfCoverage) were: 38,120,877 and 38,599,542 bp (~38.4 Mbp) for the CCG facility and 27,182,553 bp (~27.2 Mbp) for the outside facility^21^; average depth: 43× and 45× for the CCG facility and 23× for the outside facility; median insert-sizes: 167 and 200 bp for the CCG facility and 263 bp for the outside facility (calculated with Picard 1.88). Assembly was performed with BWA 0.6.2 on the UCSC hg19 reference genome. After sorting and indexing of the resulting BAM files with SAMtools, version 0.1.19, PCR duplicates were removed with Picard 1.88. Exonic regions were re-aligned and the base quality scores were re-calibrated according to the Genome Analysis Toolkit Best Practices recommendations. For ‘somatic’ comparisons we used the same-patient pair-matched germline if available, otherwise a representative germline sample obtained from the same batch (‘pseudosomatic’) was used.

For somatic single-nucleotide variants (sSNVs) MuTect 1.1.4 and MuTect v2 were employed with default parameters, while for somatic indels (insertions and deletions) VarScan 2.3.6 was used. We also used Genome Analysis Toolkit UnifiedGenotyper 2.7–4 for germline SNVs and indels. Mutations were annotated using ANNOVAR with the associated packages NCBI dbSNP 138, COSMIC 70 WGS, ESP6500-SI (W. NHLBI GO Exome Sequencing Project Seattle), 1000G April 2012, ExAc0.3 (Exome Aggregation Consortium, Cambridge, MA (http://exac.broadinstitute.org [06/08/2015 accessed via ANNOVAR])), NCI60, and clinVar release 20150330.

Pseudosomatic SNVs and small indels were filtered (i) by exclusion of potential SNPs by eliminating mutations with a 1000G and/or ESP6500-SI frequency and/or ExAc0.3 minor allele fraction (MAF) ≥0.01 (PopFreq <0.01 considered as SNV or indels), (ii) by determination of genes that are enriched for likely damaging mutations using PROVEAN (for indels), PolyPhen2 (score ≥0.957) and SIFT (score ≤0.05) algorithms, (iii) by controlling the false-discovery rate (FDR) by a statistical comparison of observed and expected mutation rates (WUSTL MuSiC), and (iv) by inclusion of mutations that are annotated within the COSMIC data base v70. For our paired WES cases, we applied more lenient filters (standard MuTect) and skipped (ii). Since we observed a high portion of C:G > A:T transversions indicative for oxidative DNA damage (8-oxoguanine (8-oxoG) lesions), we applied additional filters similar to the ones used in Costello et al. and Chen et al.^37,38^. First, we ran MuTect v2 to obtain FoxoG ratios (fraction of alternate allele supporting reads with e.g., G > T on read 1 and C > A on read 2 or vice versa; not to be confused with “strand bias”) and tumor loads (estimated log odds that the observed number of alternate allele reads from the tumor sample could have arisen from a reference allele) for each mutation we previously screened with the less stringent MuTect v1.1.4.

We discarded all mutations with tumor fractions below 0.5 that were not found by MuTect v2 (and therefore no FoxoG ratios and tumor loads available). We further discarded all mutations not surpassing the empirical filter of Costello et al.^38^ and thereby having a potential allelic imbalance: tumor loads ≥10 + (100/3) × FoxoG. A Lego plot of SNV frequencies with trinucleotide contexts was prepared using a modified source code by developer C. Wardell (https://github.com/cpwardell/ 3dbarplot).

We calculated the mutational frequency without background-correction, by dividing the average number of somatic mutations per sample per target Mb (NimbleGen SeqCap EZ Exome v3: 64,000,000 bp). Since we also ran samples on the lower targeting SeqCap2 and Agilent SureSelect Human All Exon V6, the mutational frequency is actually underestimated (conservative estimate). Mutation frequencies of other neoplasms were obtained with the same caller (‘Published validation rates of calls made by previous versions of MuTect in coding region’).

We inferred structural variations by mapping distance and order of paired-end reads using DELLY (version 0.7.2) and filtered for a minimum genotype quality of 200, for no LowQual entries, minimum depth of 5 reads, and for split-read support (more precise breakpoint localization). For structural variations affecting TCL1 oncogenes, we applied more lenient filters (genotype likely somatic). CN neutral entries in the database of genomic variants (GRCh37_hg19_variants_2013-07-23) were further used to filter within a 1 kb breakpoint window. The resulting list was then annotated with the COSMIC SV data sheet (02/04/2014 last modified; liftOver from hg38 to hg19 with UCSC Utilities web-GUI) and visualized with circos 0.64. Chromotripsis patterns were evaluated as previously described^35,36^.

For the detection of sCNAs in WES data, we used “EXCAVATOR2“ at default settings, which evaluates significant drops of coverage. As the reference set, we pooled all germline samples obtained from the same batch of the respective tumor sample. Potential microsatellite-instability (MSI) was assessed using MSIsensor with default settings.

Sequential samples were compared in a pair-wise fashion: sample at F/U vs. sample at diagnosis. Estimations of cellular prevalences and clustering of mutated populations were obtained by the Bayesian framework PyClone using its own Binomial density model with 10,000 MCMC iterations and default priors (shape = 1.0; rate = 0.001; initial concentration = 1.0; alpha = 1; beta = 1). As input, we only considered mutated genes that were among those TOP 50 that increased the most in VAF between first and second time point or those that were only mutated at second time point, and were simultaneously members of enriched pathways (*p* < 0.01; see also Supplementary Data 19): e.g., “TP53 Regulates Transcription of DNA Repair Genes” (Wikipathways), “Notch Signaling Pathway” (Wikipathways), “TP53 Regulates Transcription of DNA Repair Genes” (Reactome), “TCR” (NetPath), “Interleukin-4 and −13 signaling” (Reactome), and “JAK STAT MolecularVariation 2” (INOH).

### Whole-genome sequencing (WGS)

Sample preparation: DNA extraction was performed as described under "Whole-exome sequencing (WES)". Sample processing for WGS was performed according to standard techniques.

Bioinformatics: We sequenced 3 T-PLL t/g-pairs and one T-PLL tumor single on an Illumina HiSeq2000 using the same settings as for WES analysis, except for different target regions for alignment and mutation calling, including non-coding (nc) regions. The Broad Institute hg19 Catalog of long-intergenic non-coding RNAs, Gencode lncRNAsv7 summary table (05/02/2012 accessed), mirBase Release 20 (around 2000 validated and over 4000 predicted miRNAs), FANTOM5 hg19 enhancer sites (accession 29/11/2012), and promoter regions derived from version 71 of the Ensembl annotation (−2000 to +200 bp of TSS) were used. Telomere lengths were analyzed using ‘telseq’.

### Whole-transcriptome sequencing (WTS)

Sample preparation: PBMCs of T-PLL patients (>95% purity of T-cells) and CD3^+^ T-cells isolated from PB of healthy donors (see “Magnetic-bead based cell enrichment“ for details on cell purifications) were subjected to RNA isolation using the mirVana kit (Invitrogen). WTS analyses were conducted using the Illumina HiSeq2000 platform according to standard techniques.

Bioinformatics: Reads were mapped to the human reference genome, build GRCh37, using Tophat v2.0.10 and the genome annotation based on the Ensembl data base, version 75. After duplicate removal, the read counts were further processed using DESeq v1.14.0 and DEXSeq v1.16.0 to analyze differentially expressed and differentially spliced genes between all 15 T-PLL samples and 4 healthy-donor derived control T-cell samples. Fusion events were analyzed using Tophat-Fusion and the associated downstream filtering pipeline (Tophat-Fusion Post). Alternatively with less stringent quality filters, but with calculation of oncogenic potential, we used oncofuse with two complementary filters: passenger probability <0.001, driver probability >0.999 and minimum support reads >10, as well as passenger probability <0.01, driver probability >0.99, and minimum support reads >100. In a validation approach we aligned reads with STAR_2.5.2a in 2-pass mode to the GRCh37/hg19 reference genome. Sub-routine STAR-Fusion was used to evaluate fusion transcripts. General overlap to results obtained by TopHat-Fusion was quite low (sample-wise: 21/96; global by gene partners: 30/96); however, all prominent hits were confirmed: *TCL1A-TRAJ49* as well as *PLEC* with other genes on chr.8 (i.e., *ZC3H3* or *SHARPIN*). WTS samples were screened for SNVs (as anchor points for allele-specific expression) with GATK UnifiedGenotyper 2.7–4 and very low quality thresholds (–filter_mismatching_base_and_quals–filter_reads_with_N_cigar-stand_call_conf5-stand_emit_conf2). Cuffdiff (cufflinks-2.2.1.Linux_x86_64) was used to generate FPKM values (fragments per kilobase of exon per million reads mapped).

VirusFinder2.0, did not identify any viral transcripts except for J02482/Coliphage phi-X174, a control in the sequencing run. The integration-site file was empty and whole-genome screens did not reveal viral sequences.

Differential exon usage was analyzed using the DEXSeq package, version 1.16.0. Exons were selected using 5 as a cutoff for linear fold enrichment and 0.01 for the adjusted *p*-value, ending up with a list of 1927 alternatively used exon segments in 1091 genes. Among these alternative splicing events, 140 exons in 85 genes overlapped with events reported for acute myeloid leukemia (AML) and 105 exons in 67 genes overlapped with events reported for diffuse large B-cell lymphoma (DLBCL) according to the TCGA SpliceSeq data base. Alternative splicing events in 56 genes are overlapping between all 3 hematologic malignancies (T-PLL, AML, and DLBCL).

### Targeted amplicon sequencing (TAS) and Sanger sequencing

T-PLL tumor singles of 18 cases were analyzed by a customized targeted-amplicon sequencing (TAS) panel that we designed. It covered *ATM* (ex.1–63), *JAK1* (ex.9–15), *JAK3* (ex.10–17) using the Illumina MiSeq platform, and *STAT5B* (ex.16) using Sanger sequencing (see Supplementary Data 22 for oligonucleotides).

Sample preparation: Amplicons were generated using standard PCRs. Products were purified using the ZR-96 DNA Clean-up Kit (Zymo Research), and an equimolar amplicon-pool was prepared for each patient. Library preparation was conducted using the TruSeq DNA LT Sample Prep Kit (Illumina) with 1 µg amplicon DNA. Amplification was carried out using 8 cycles. The MiSeq Reagent Kit v3 (Illumina) was used for sequencing and the samples were analyzed on the MiSeq NGS platform. Library preparation and sequencing was performed according to the manufacturer's instruction at the Cologne Center for Genomics (CCG).

Bioinformatics: For read alignment and further read processing, we followed the same strategy as for WES (above). The Genome Analysis Toolkit Unified Genotyper 2.7–4 was used without down-sampling (dCov = 10,000) to call mutations (SNVs and indels). We calculated the VAF with “bam-readcount” (https://github.com/ sjackman/bam-readcount, accessed 19/12/2014) with minimal mapping and a base quality of 20. A Phred-scaled quality of at least 100 and a depth of coverage of at least 10 was presumed to restrict false positives. We further used the same filters as for pseudosomatic mutations in WES.

Sanger sequencing: Primer spanning all regions of interest were designed and used for PCRs according to standard protocols. PCR products were sequenced using the Big Dye Terminator Sequencing v3.1 kit and ABI PRISM 3730XL DNA Analyzer (Applied Biosystems). Capillary electrophoresis was carried out at the CCG. For electropherogram analysis SnapGene (v2.8.2, SnapGene) and 4Peaks (v1.8, nucleobytes) were used.

### Integrative approaches of bioinformatic analyses

Major analysis steps were executed through our own ‘Cancer Pipeline’^72^ within the QuickNGS framework and downstream Semantic Web applications. Thus, mutation analysis results are written in the RDF/N3 (resource description framework) format, and stored in a jetty-6.1.26 servlet engine running an OpenRDF Workbench Version 2.6.10 Sesame server. Combinatorial (with patient data) and multiple data set analyses (Figs. 4f, 5b, 6a, and Supplementary Figs. 6,7, as well as sample organization was done by implementing queries that were further processed with the R-package “SPARQL 1.16”.

### Quantitative real-time PCR

Total RNA was extracted from human CD3^+^ pan T-cells and murine CD8^+^ T-cells following manufacturer’s instructions (mirVana, Invitrogen and RNeasy Mini Kit, Qiagen). Total sample RNA was reverse-transcribed into polydT cDNAs using SuperScript II reverse transcriptase (Thermo Fisher Scientific). Real-time quantitative PCR on human and murine mRNA was carried out using an ABI 7500 Fast System. Primers of the genes encoding human and murine β-actin were used as standard references for quantification using the 2^(-Delta Delta C(T))^ method (see Supplementary Data 22 for Oligonucleotides).

### Cell cultures and cell lines

RPMI-1640 medium (Sigma-Aldrich) supplemented with 2 mM l-Glutamine, 10% fetal bovine serum (FBS) and Penicillin/Streptomycin (100 U/1) was used for in vitro experiments on suspension cultures of primary T-PLL cells, the T-cell lines HH/iHH-TCL1A and HuT78/Hut78-TCL1A, the *A-T* derived B-lymphoblastoid cells, and the 32D cells. Suspension cells were maintained at a density of 1.0–3.0 × 10^5^ cells/ml and of 1.0 × 10^6^ cells/ml (T-PLL cells). ATM^fl/wt^ and ATM^fl/KD^ MEFs (mouse embryonic fibroblasts) were cultured in DMEM supplemented with 2 mM l-Glutamine, 15% FBS, 1% MEM non-essential amino acid, 1% Sodium Pyruvate, 0.12 mM 2-Mercaptathanol and Penicillin/Streptomycin 100 U/1 (PAA). Culturing was done in an incubator at 37 °C and 5% CO_2_ with 90% humidity. HEK293T cells were propagated in complete DMEM medium (10% FCS, 2 mM l-Glutamine, 10 U/ml Penicillin/Streptomycin). The cell lines HH (ATCC; https://www.lgcstandards-atcc.org), Hut78 (ATCC) and the *A-T* patient-derived lines (gift of L. Chessa, Rome, Italy) were originally acquired in 2011 and before. Only original stock propagated immediately upon arrival for 2–3 passages was picked for studies and cultures terminated after the 10th round of passaging (4–6 weeks). Upon thawing for experimentation in years 2011–2017, all lines were authenticated by characteristic growth behavior and by flow cytometry confirming their characteristic immunophenotype. Each thawed passage was tested for *Mycoplasma* infection by standard PCR protocols. All used cell lines are not listed in the latest version 8.0 of the register of cell lines that are known to be misidentified through cross-contamination or other mechanisms.

HH/iHH-TCL1A and Hut78/Hut78-TCL1A systems from human CD4^+^ mature (cutaneous) T-cell leukemia lines: iHH-TCL1A cells of inducible TCL1A expression were created via genetic modification of the parental HH line (TCL1A negative) by transfection with retroviral expression vectors (TRMPVIR system) encoding for human TCL1A under control of the Doxycycline-inducible tet-on promotor (Supplementary Fig. 17) and by subsequent Puromycin-based selection. TCL1A was induced in iHH cells by exposure to 1 µg/ml doxycycline for 24 h or if longer then indicated. Stable Hut78-TCL1A transfectants were created through genetic modification of the Hut78 (TCL1A negative) line by transfection with retroviral expression vectors (MSCV-puro) encoding for human TCL1A and subsequent Puromycin-based selection.

The *A-T* patient-derived B-lymphoblastoid cell lines^73^ ‘AT65RM’ (ATM^Δ/Δ^: c.6573-9G->A/ c.8814_8824del11; ATM protein absent) and ‘AT-CT’ (*ATM*^*WT*^ control from an unaffected relative) were used to assess (ATM related) specificity of pKAP^Ser824^ induction.

For detection of levels of reactive oxygen species (ROS) 6-well plates were coated with anti-CD3 (OKT3, 10 µg/mL) and anti-CD28 (15E8, 20 µg/mL) in PBS for 1 h at 37 °C. The solution was gently aspirated and T-PLL cells (1 × 10^6^/ml) were added. Flow-cytometry based analysis of intracellular ROS levels was conducted using the H_2_DCFDA dye according to manufacturer’s instructions. Measurement of ROS induction in MEF and Hut78(-TCL1A) cells after irradiation (5 and 15 Gy) was performed after indicated timescales using the ROS dye H_2_DCFDA, which was added in a final concentration of 1 µM and incubated for 30 min at 37 °C. Measurement was done in a plate reader or via flow cytometry.

*p*-STAT5B responses of T-PLL cells and CD3^+^ pan T-cells from healthy donors to interleukins was assessed using IL-7 (5 ng/mL), IL-15 (20 ng/mL), and IL-21 (100 ng/mL), all from PreproTech. Cells were treated with interleukins for 30 min.

### Plasmid mutagenesis and transfection

*STAT5B* variants were generated by site-directed mutagenesis (QuikChange Lightning Site-Directed Mutagenesis Kit, Agilent Technologies) using a sequence verified FLAG-tagged human STAT5B pMSCV-IRES-GFP vector. For primers used please refer to Supplementary Data 22. Sequence and mutations were verified by Sanger sequencing. Plasmid transfection of HEK293 cells was performed using SuperFect reagent (Qiagen). 32D cells were electroporated using 5 × 10^6^ cells in 800 µl plain RPMI medium containing 20 µg plasmid DNA. Following electroporation, cells were cultured with RPMI medium containing 20% FCS and 2 ng/ml IL-3 for 2 days. 5 × 10^3^ electroporated 32D cells were seeded with or without 1 ng/ml IL-3 and viability was assessed using CellTiter-Glo Luminescent Cell Viability Assay (Promega).

ATM^fl/wt^ and ATM^fl/KD^ mouse embryonic fibroblasts (MEFs)^43^ were used to generate their TCL1A-expressing sub-lines. The TCL1A expression construct was generated by an insertion of the TCL1A-encoding cDNA sequence into a commercially available PiggyBac expression vector pJ547-17 (DNA2.0, USA). Following Amaxa-based nucleofection (Lonza, Germany) of the TCL1A expression construct, TCL1A-expressing MEF sub-lines were established by the selection of the stable clone with hygromycin. Transfection efficacy was controlled via TCL1-APC staining using flow cytometry analysis. Cells were passed three times (48 h interval) in the presence of 4-hydroxytamoxifen (4OHT, 200 nM) to achieve respective genotypes via the 4OHT-inducible Cre recombinase (performed separately for each biological replicate). Genotyping was done using a standard PCR protocol. For viability assays, MEF cells were seeded in 96-well plates (6 × 10^3^cells/well) and irradiated with 5 or 15 Gy. After 48 h the colorimetric MTT assay was performed (for details see “in vitro drug treatment and cell viability”).

### FISH analysis and karyotyping

FISH analysis was conducted according to manufacturer’s instructions using probes targeting *AGO2* (customized at Empire Genomics, Custom FISH Probe, Clone Library: RPCI-11 (RP11), Clone Name: 628B24), *CEP8* (Metasystems, XCE8, D-0808-050-OR), and *TCRα*/*TCRδ* sequences (LSI TCR alpha/delta dual color Break Apart rearrangement Probe, 05N41-020, Abbott Molecular). The latter probe set was used to supplement karyotypic data in order to confirm inv(14) or *t*(14;14) associated rearrangements of *TCR* gene elements as part of the aberrations that activate *TCL1A* expression. For metaphase karyotyping, PB samples were cultured for 72 h under PHA-mediated stimulation. Cells were arrested using colcemid (Biochrom) and then treated with 0.3% potassium chloride solution followed by fixation in ice-cold Carnoy’s reagent. Cell suspensions were dropped onto microscopic slides. Dried samples were stained using GTG-banding. For interphase FISH analysis, unstimulated cells from PB were used. Slides were prepared as described above. DNA was denatured for 5 min at 73 °C in denaturing solution (70% formamide (Sigma), 10% 20X SSC, and 20% water). Slides were washed in 2X SSC and dehydrated in a 70%, 85%, and 100% ethanol series for 2 min each. Volumes of 10 µL of specific FISH-probe were applied to the slides and coverslips sealed with rubber cement. Following overnight incubation at 37 °C in a wet chamber, slides were washed in 0.4X SSC/ 0.3% NP-40 for 2 min at 73 °C and in 2X SSC/0.1% NP-40 for 1 min at room temperature. Volumes of 10 µL of DAPI counterstain solution (Metasystems) were applied to air-dried slides, which were then sealed using a coverslip and nail polish.

### Telomere length evaluation

Flow-FISH analyses for telomere length assessment were conducted as follows^74^: samples were prepared for cell denaturation and mixed with a FITC labeled telomere specific (CCCTAA)3-peptide nucleic acid FISH probe (Eurogentec) for DNA-hybridization followed by DNA counterstaining with LDS 751 (Sigma). Bovine thymocytes were used as an internal control. Data acquisition was done with a FC-500 flow cytometer (Becton Dickinson). All measurements were carried out single-blinded in triplicates. Healthy control lymphocytes (104 volunteers) were used for age-adaptation of telomere length. Monochrome multiplex quantitative PCR (MM-QPCR) for determination of telomere length was performed as previously described^75^ (see Supplementary Data 22 for oligonucleotides). T/S ratios were calculated by dividing the number of copies of the telomere template (T) by the beta-globin template (S)^75^.

### In vitro drug treatment and cell viability

The MDM2 inhibitor Idasanutlin (Hycultec), the mutant p53 reactivator Prima-1^met^ (GENTAUR), the HDAC inhibitor Panobinostat (Hycultec), the ATM inhibitor KU55933 (Selleckchem), the alkylating agent Bendamustine (Astellas Pharma), and the PARP inhibitor Olaparib (Selleckchem) were solved in dimethyl sulfoxide (DMSO). For key information on the small molecule inhibitors used, including primary references, see Supplementary Table 3. Drug exposures were performed at the indicated concentrations and times. Dosing was based on published ranges and own IC50/LD50 titrations. Apoptosis was determined using dual staining for Annexin-V (AnnxV) and 7AAD via flow cytometry. The colorimetric MTT (3-(4,5-dimethylthiazol-2-yl)-2,5-diphenyltetra-zolium bromide) assay measured using SpectraMax Paradigm Plate Reader, assessed metabolic activity of cells and by that viability.

Screening for compound interactions was performed on mononuclear cell samples from 13 T-PLL patients. Substances were dissolved in DMSO and dispensed on 384-well plates using Echo 550, an acoustic liquid handling device. Each drug combination was plated in duplicates in a matrix of 7 concentrations covering a 1000-fold concentration range. The compounds were dissolved in a shaker with 5 μL of Mononuclear Cell Medium (Promocell) for 10 min. Twenty microliters of single-cell suspension (equivalent to 10,000 cells) were transferred to each well using a MultiDrop Combi dispenser. The plates were incubated at 37 °C and 5% CO_2_, and after 72 h cell viability was measured using the CellTiter-Glo luminescent assay and a Pherastar FS plate reader. The 6 drug combinations were screened in 384 well plates for each sample. The plate layout consists of 6 wells for positive controls (100 μM Benzethonium Chloride) and 6 wells negative controls (DMSO), distributed throughout the plate. Relative percent inhibition is calculated by subtracting each well from negative control, divided by sum of average positive and average negative control. The delta score of drug combinations, which is based on newly developed zero-interaction potency (ZIP) reference model, is computed using synergyfinder R-package.

### Irradiation response

Cell lines and primary T-PLL cells were cultured in standard RPMI-1640 medium (see “Cell cultures and cell lines”) and DNA damage was induced using gamma irradiation at the indicated Gy-dosages by a BIOBEAM GM instrument (Gamma-Service Medical) equipped with a Cs137 radionuclide source. After irradiation, cells were incubated for indicated timescales at 37 °C, 5% CO_2_ and subsequently collected for immunoblotting, flow cytometry, MTT assay, or ROS staining, and so on.

### Immunoblots

Western blots on whole-cell protein lysates were performed according to standard techniques. The primary antibodies included: ATM (clone D2E2), phospho-STAT5B^Tyr694^ (clone C11C5), STAT5B (polyclonal; #9363), phospho-JAK1^Tyr1022/1023^ (polyclonal; #3331), JAK1 (clone 6G4), phospho-JAK3^Tyr980/981^ (clone D44E3), JAK3 (clone D7B12), phospho-p53^Ser15^ (clone 16G8), p53 (clone 1C12), acetyl-p53^Lys382^ (polyclonal; #2525), acetyl-Histone H3^Lys18^ (clone D8Z5H), PARP (polyclonal¸ #9542), γH2AX^Ser139^ (clone 20E3), phospho-TIF1beta^Ser824^ (pKAP1; polyclonal; #4127), TIF1beta (KAP1; clone 4E1), phospho-Chk2^Thr68^ (clone C13C1), Chk2 (clone 1C12), and GAPDH (clone 14C10), all from Cell Signaling Technology; ATM (clone 2C1), β-actin (clone c-11), β-tubulin (polyclonal; sc-9104), and HSC70 (clone B-6), all from Santa Cruz Biotechnology; phospho-ATM^Ser1981^ (clone EP1890Y) from LifeSpan BioSciences, c-Myc (R951-25) from Dianova, and MDM2 (clone IF2) from Calbiochem. Development and use of our anti-TCL1A antibody (clone 1–21) in T-PLL has been described^67^. Ago2 antibody (clone 11A9; diluted 1:5–10) was a gift from G. Meister (University of Regensburg, Germany). All primary antibodies were used at 1:1000 dilutions, except for anti-GAPDH (1:3000 dilution), anti-phospho-Chk2 (1:750), anti-Chk2 (1:500), anti-MDM2 (1:300), anti-β-tubulin (1:5000), and anti-β-actin (1:5000). As secondary HRP-coupled antibodies we used: anti-goat (sc-2020), anti-rat (sc-2303), anti-mouse (sc-2314) and anti-rabbit (sc-2313), all from Santa Cruz Biotechnology, according to the manufacturer’s instructions. Western blots were developed using Western Bright ECL (Advansta). Chemiluminescence was detected using Autoradiography Film Blue (Santa Cruz Biotechnology), and the developer machine CAWOMAT 2000 IR (CAWO Solutions). Signal intensities were recorded by densitometry (ImageJ software). Supplementary Fig. 20 provides uncropped images of all immunoblots shown in the main figures (Figs. 7c, 9b, c).

### Immunofluorescence microscopy

Cytospins were prepared using 1 × 10^5^ primary T-PLL or HH/iHH-TCL1A cells in a Cytospin3 cytocentrifuge (Thermo Shandon) at 800×*g* for 5 mins. Cells were fixed for 15 min at 4 °C in 3% PFA with 2% sucrose in PBS. Cell permeabilization (10 mM PIPES, pH 6.8, 100 mM NaCl, 300 mM sucrose, 3 mM MgCl_2_, 1 mM EDTA, 0.5% Triton X-100), and cytoskeleton stripping (10 mM Tris-HCl, pH 7.4, 10 mM NaCl, 3 mM MgCl_2_, 2% Tween20, 0.5% sodium deoxycholate) were performed on ice each for 10 min. Blocking was carried out using 5% BSA/PBS for 45 mins at room temperature. Primary antibodies against yH2AX (clone JBW301 Millipore/Merck Chemicals), RAD51 (polyclonal; #ab63801, Abcam), TP53BP1 (Cell Signaling Technology), 8-Hydroxyguanosine (clone N45.1, Abcam), and ATM (clone 2C1, Santa Cruz Biotechnology) were used at 1:200 dilution in 5% BSA/PBS overnight at 4 °C in a wet chamber. The secondary antibodies donkey anti-mouse (AF488 labeled) and donkey anti-rabbit (Cy3 labeled) (Jackson Laboratories/Dianova) were diluted at 1:400 in 5% BSA/PBS. Incubation was carried out for 3 h at room temperature. Slides were washed three times for 10 min with 5% BSA/PBS and once shortly with PBS to remove BSA. Slides were coverslipped with Mowiol containing Hoechst 33258 (140 μM). Samples were analyzed using an Axio Scope.A1 fluorescence microscope (Zeiss). Representative images were captured using AxioVision software. Quantification of yH2AX, RAD51, and TP53BP1 foci was performed by manually counting the foci in 30 nuclei per time-point (means with SEM calculated). Cytosolic or nuclear ATM localization was assessed by measuring fluorescence intensity using the ImageJ software. Fluorescence signals derived from the whole cell and from the nucleus were determined separately in 5 cells per sample and condition. Whole-cell fluorescence was set to 100% to calculate the percentile distribution of nuclear fluorescence intensity. 8-Hydroxyguanosine (8-OxoG) levels were accordingly determined over 30 whole cells.

### Data availability

Deep sequencing data supporting the findings of this study are subject to controlled access at the European Genome-phenome Archive (EGA) with accession number EGAS00001002744. SNP-array and microarray-based GEP data are available at Gene Expression Omnibus (GEO) with accession number GSE107513. WES data on 8 t/g pairs^21^ and TAS data for *JAK/STAT* genes on 4 cases^62^ had been published previously.

## Electronic supplementary material

Supplementary Information

Description of Additional Supplementary Files

Supplementary Data 1

Supplementary Data 2

Supplementary Data 3

Supplementary Data 4

Supplementary Data 5

Supplementary Data 6

Supplementary Data 7

Supplementary Data 8

Supplementary Data 9

Supplementary Data 10

Supplementary Data 11

Supplementary Data 12

Supplementary Data 13

Supplementary Data 14

Supplementary Data 15

Supplementary Data 16

Supplementary Data 17

Supplementary Data 18

Supplementary Data 19

Supplementary Data 20

Supplementary Data 21

Supplementary Data 22
