# Supplementary material for: Actionable perturbations of damage responses by TCL1/ATM and epigenetic lesions form the basis of T-PLL
Source: Nat Commun. 2018 Feb 15;9:697. doi: 10.1038/s41467-017-02688-6 (PMC5814445; doi:10.1038/s41467-017-02688-6)
Supplement: Supplementary file 1 — Supplementary Information [file 41467_2017_2688_MOESM1_ESM.pdf]

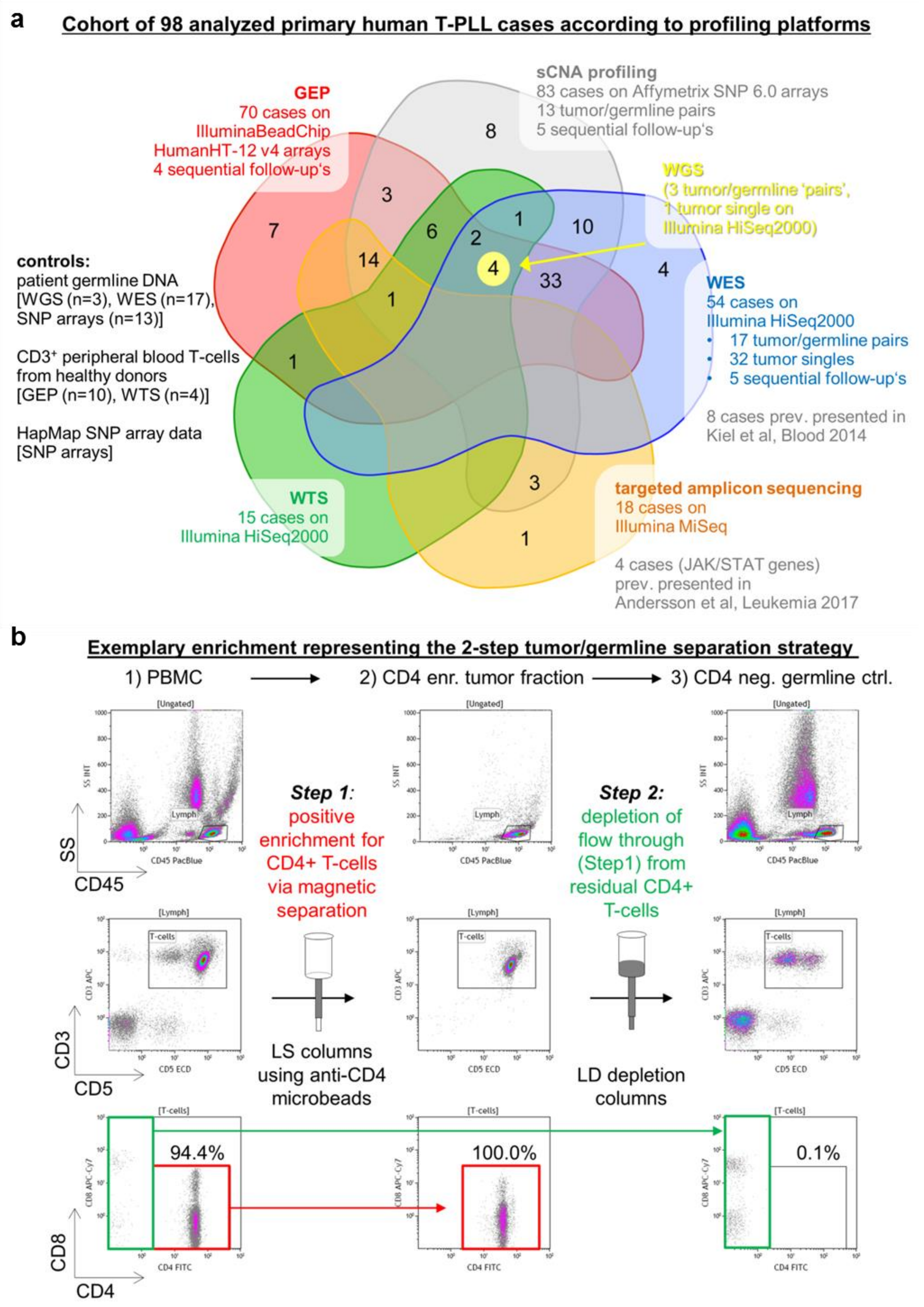

Supplementary Figure 1: Study cohort of 98 T-PLL and controls - platforms and cell isolation

legend on next page

Supplementary Figure 1: Study cohort of 98 T-PLL and controls - platforms and cell isolation

Note that samples from 98 patients were subjected to genomic analyses, but an overall of 111 patients were included in this study accounting also for those on which only *in vitro* experimentation was performed.

a) Purified T-cells from 98 T-PLL patients (**Supplementary Data 1** for additional information) were analyzed using various high-throughput profiling platforms (overlap indicated): Illumina HumanHT-12 v4 BeadChip arrays (n=70 cases) for gene expression profiling (GEP), Affymetrix SNP 6.0 arrays (n=83 cases) for analysis of somatic copy-number alterations (sCNAs), and the Illumina HiSeq2000 next-generation sequencing (NGS) platform. On the latter, whole-genome sequencing (WGS; n=3 matched pairs of same-patient tumor/germline (t/g) DNA and 1 tumor single), whole-exome sequencing (WES; n=17 t/g-pairs in addition to n=37 tumor singles including 5 cases with sequential follow-up (F/U) samples), and whole-transcriptome sequencing (WTS; n=15 tumors) were performed. Further cases (n=18 tumor 'singles') were analyzed by a customized targeted amplicon sequencing (TAS) panel including *ATM* (exons 1-63), *JAK1* (exons 9-15), and *JAK3* (exons 10-17) using the Illumina MiSeq platform and *STAT5B* (exon 16) analyzed via Sanger-sequencing based methods. $CD3^+$ pan T-cells isolated from peripheral blood (PB) of healthy donors with a similar age-median were used as "normal" controls for GEP (n=10) and for WTS (n=4). For sCNA profiling patient-derived germline control DNA from 13 t/g pairs of the 83 cases) were used as a pooled reference alone or in combination with publically available HapMap data sets (http://hapmap.ncbi.nlm. nih.gov/).

b) The isolation strategy of PB tumor cells and matched same-sample germline controls from PB mononuclear cells (PBMCs) of T-PLL patients employed a two-step magnetic separation (MACS columns) process (shown is case TP010). (1) Positive enrichment of T-PLL tumor cells: magnetic beads bound to anti-CD4 or anti-CD8 antibodies (Microbeads, Miltenyi Biotec) and LS Columns (Miltenyi Biotec) were used. The specificity of beads was selected according to the individual immunophenotype. (2) Depletion of residual T-PLL cells from the flow-through designated as normal control: Depletion Columns (LD, Miltenyi Biotec) were used to remove residual CD4 or CD8 positive cells from the flow-through obtained from step 1. For further details, see **Methods** section.

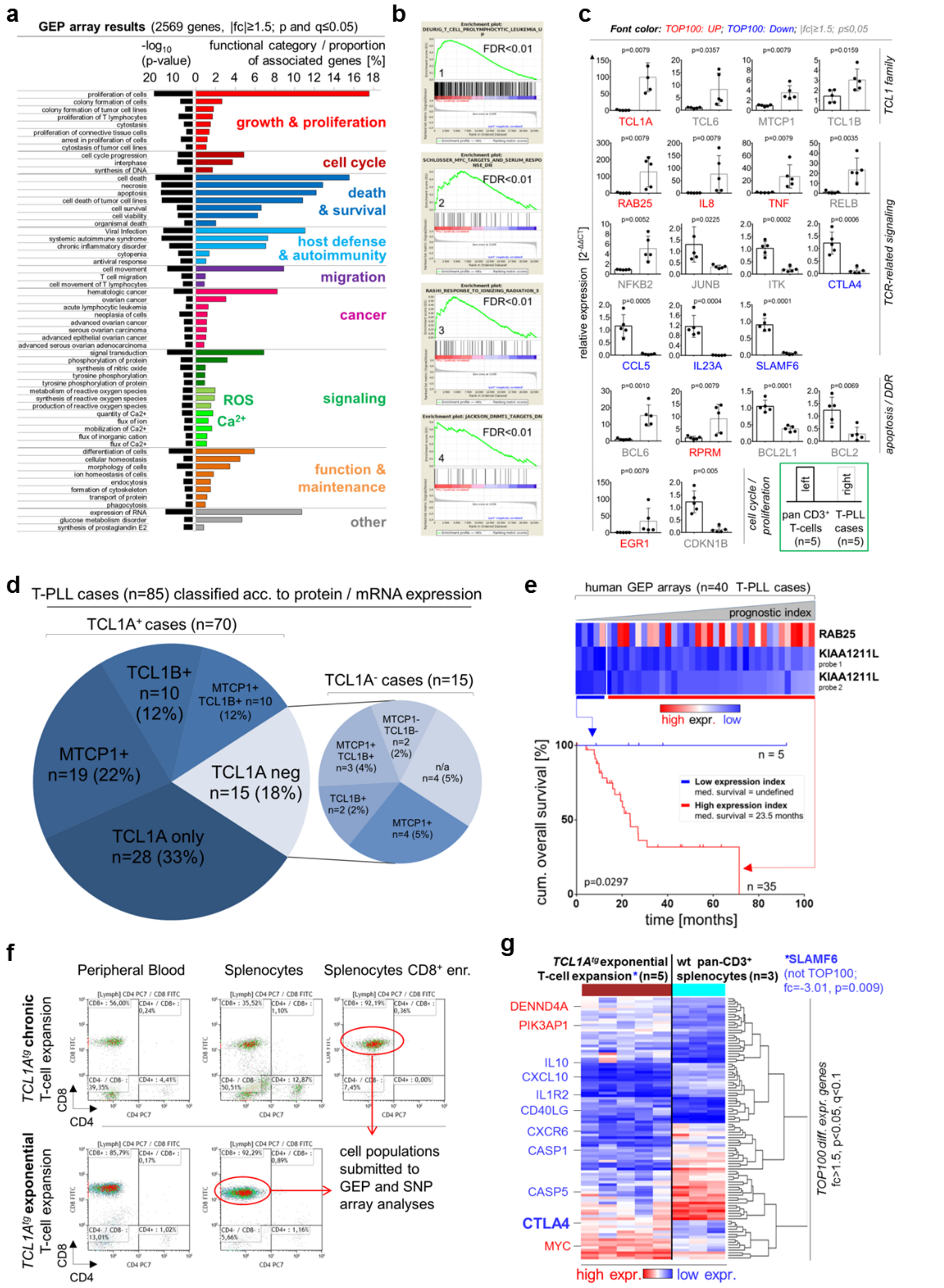

Supplementary Figure 2: Functional annotations of differentially expressed genes in T-PLL with technical (qRT-PCR) and biological (*Lck*^{pr}-*TCL1A*^{tg} mice) validations

legend on next page

Supplementary Figure 2: Functional annotations of differentially expressed genes in T-PLL with technical (qRT-PCR) and biological (*Lck^{pr}-$TCL1A^{tg}$* mice) validations

a) Affiliation of differentially expressed genes (2569 genes; |fc|≥1.5; p ≤0.05; q ≤0.05) to functional groups in Ingenuity® Pathway Analysis (IPA): proportion of genes [%] associated with the respective process in relation to the total number of differentially expressed genes and specific p-values (black bars). Gene sets belonging to the functional groups of 'growth and proliferation', 'death and survival', 'host defense and autoimmunity', or 'ROS/Ca^{2+} signaling intermediates' were significantly enriched (see **Fig.1a** for a heat map of Top100; **Supplementary Data 2** for all differentially expressed genes).

b) To test whether gene sets previously identified to be deregulated in T-cell malignancies or associated with T-PLL are differentially expressed in our set as well, we analyzed for overlaps using the Broad Institute's GSEA[1,2] platform in addition to general annotations by IPA (FDR<0.01, n=22 gene sets; across all MSigDB gene sets). Four examples of identified functional relevance to T-PLL show significant enrichments of genes that were: (1) previously associated with T-PLL (transcriptomes of 8 $CD3^+$ normal donor-derived PB cell samples *vs.* 5 T- PLL[3]), (2) identified as MYC targets (transcriptional program of lymphocytes in response to MYC expression[4]), (3) activated by ionizing radiation regardless of *ATM* status in murine lymphoid tissue[5], and (4) identified to be targets of epigenetic modification (microarray analyses of fibroblasts from *DNMT1* knockout mice[6]).

c) qRT-PCR validations of GEP data, including genes encoding *TCL1* family members (for *TCL6* an independent gene status is still controversial[7]), TCR-related signaling molecules, and apoptosis-/DDR-associated factors (5 T-PLL *vs.* $CD3^+$ pan T-cells from PB of 5 healthy donors).

d) TCL1 gene family status by protein / mRNA: TCL1A and/or MTCP1 pos. in 90.6% (n=77/85) *vs.* neg. or n/a in 9.4% (8 cases). Of the latter, 2/8 showed elevated TCL1B expression, 2/8 were negative for all 3 TCL1 family members, and for 4/8 no additional data other than lack of TCL1A protein was available (n/a). Genomic data: inv(14)/t(14;14) present in 87.5% (n=49/56); t(X;14) in 7.1% (n=4/56). Suggesting an impact of constitutive *$MTCP1^{p13}$* similar to the one by *TCL1A*, there was a considerable overlap of differentially expressed genes (e.g. *CTLA4, SLAMF6*) between TCL1A-positive cases and those 4 carrying an *MTCP1*-activating t(X;14). Further implicating a 'uniform' transcriptome of T-PLL, the GEPs of the 2 exclusively TCL1B-positive cases were similar to those of TCL1A-positive or *MTCP1*-rearranged T-PLL (229 of 412 probes).

e) Differential clinical course prognosticated by a 2-gene/3-probe expression index at the time of diagnosis, given the notion of T-PLL as a disease with a generally short survival, but with recognition of rare indolent phases. Rationale: the prognostic information by *TCL1A* was derived from high expression levels with rather moderate variability (**Figs.1a,b**). Therefore, we performed additional regression modeling based on global gene expression changes to more sensitively infer on indolent phases or particularly aggressive courses through identification of prognosis-associated genes with a wider range of expression. A most informative index of 2

differentially expressed genes (*RAB25*, *KIAA1211L*) originated from a learning cohort. It provided high discriminatory power towards clinical outcome based on stratified index values in the subsequent test cohort. Top: mRNA levels of *RAB25* and both *KIAA1211L* probes (*RAB25* or *KIAA1211L* alone of insufficient power) as the 2 signature genes filtered through regression from the learning-set of T-PLL subjects (**Methods**). Below: Kaplan-Meier curves as application of the stratified index to the test cohort discriminating outcome based on low *vs.* high index values. The oncogenic RAS GTPase *RAB25* was part of the Top100 T-PLL signature (**Fig.1, Supplementary Data 2**) providing normal-T *vs.* tumor-cell distinction.

f) Lck^{pr}-$TCL1A^{tg}$ T-cells and those of age-matched C57BL/6 (wild-type) mice were enriched from splenic lymphocytes by MACS® protocols. Stages: 'chronic phase' (30-70% tumor cells in PB and spleen, average age 12 months, n=3) and 'exponential phase' (mean PB lymphocyte doubling time (LDT) 12 days (SEM 0.8); >80% tumor cells in PB, >90% in spleen, average age 15 months, n=5). Examples for cell populations submitted to GEP (**Fig.1c, Supplementary Fig.2g**) and used in immunoblots (**Supplementary Fig.6e**).

g) GEP of *TCL1A*-induced murine T-cell leukemia at 'exponential phase' (enriched splenic $CD8^+$ T-cells) using Affymetrix GeneChip Mouse Gene 1.0 ST Arrays. Purified splenic $CD3^+$ pan-T-cells isolated from C57BL/6 mice (3 arrays from T-cell pools of 3 mice each (total n=9) were used as matched controls. Besides the commonly affected TCR signaling modulators *SLAMF6* and *CTLA4*, we observed an additional deregulation of T-PLL characteristic oncogenes (e.g. *MYC*) in overt murine leukemia at the exponential growth phase. See also **Fig.1c** showing the differential expression of genes in 'chronic-phase' expansions and **Supplementary Data 3** listing all differentially expressed genes.

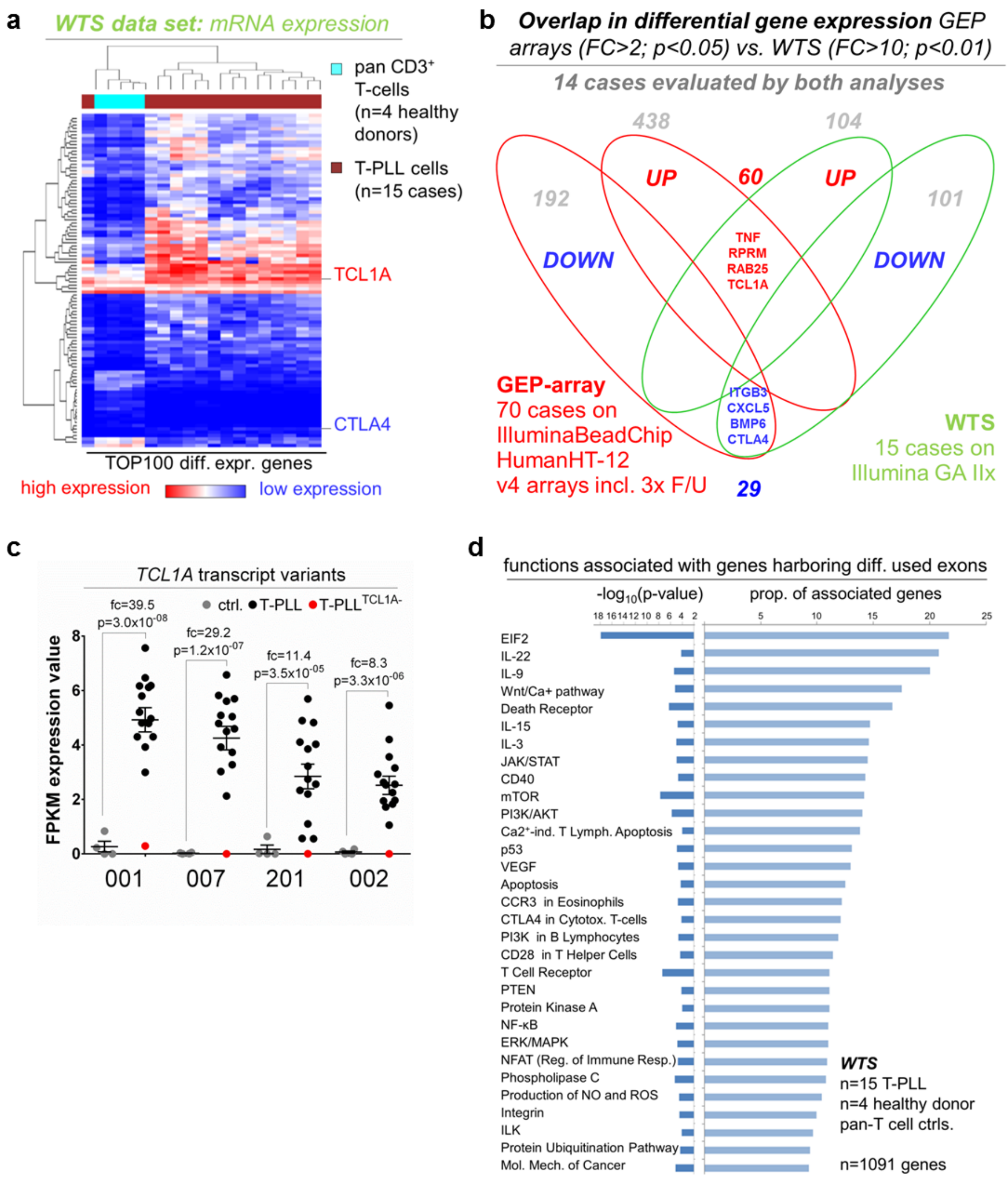

Supplementary Figure 3: WTS confirms patterns of differential gene expression and reveals differential exon usage of genes associated with TCR / cytokine signaling and p53 mediated apoptosis

a) The Top100 most variably expressed transcripts, based on the comparison of WTS data from 15 T-PLL to those from $CD3^+$ pan T-cells isolated from PB of healthy donors (n=4) are represented in a heat map (compare **Supplementary Data 4**).

b) Overlap of significantly differentially expressed genes in T-PLL cells as detected by WTS data (15 T-PLL) *vs.* GEP arrays (n=70 cases); **Supplementary Data 4** for further details.

c) Differential expression of variant *TCL1A* transcripts in primary T-PLL (n=15) compared to healthy-donor derived $CD3^+$ T-cells (‘ctrl.’, n=4) revealed a congruent

upregulation of all detected *TCL1A* transcripts in ‘*TCL1A* positive cases’ and identifies high expression of a new shorter *TCL1A* variant (*TCL1A-007*). Red dots for TCL1A-protein negative case. Detected transcripts (p-values as per Student´s t-test): TCL1A-001 (exons 1, 2, 3; 1,391 bps; 114 aa residues; fc=39.27, $p=3.1x10^{-8}$), a truncated TCL1A-007 (exons 2, 3; 980 bps; 53 aa residues; fc=29.2, $p=1.2x10^{-7}$), TCL1A-201 (exons 1, 2, 3; 1,395 bps; 114 aa residues; fc=11.4, $p=3.4x10^{-5}$), and TCL1A-002 (exons 1, 2, 4; 717 bps; 99 aa residues; fc=8.3, $p=3.4x10^{-6}$). FPKM: fragments per kilobase of exon per million reads mapped (mean with SEM). Generally, differential expression of transcripts was assessed using DESeq v1.14.0 by evaluating the expression of respective isoforms through a gapped alignment.

d) Differential exon usage (DEU) as alternative splicing, evaluated via DEXSeq v1.16.0, provides a descriptive assessment of whether the particular exon bins (containing merged exons for ORF overlaps of multiple genes) are rather retained or skipped. It also includes corrections for potential biases through differential expression. Here, differentially spliced genes (see **Supplementary Data 5,** FDR cutoff q<0.01) identified by comparing WTS data of primary T-PLL cells (n=15) to healthy-donor T-cells (n=4) were associated with TCR / cytokine signaling and p53 mediated apoptosis, as evaluated by ConsensusPathDB.

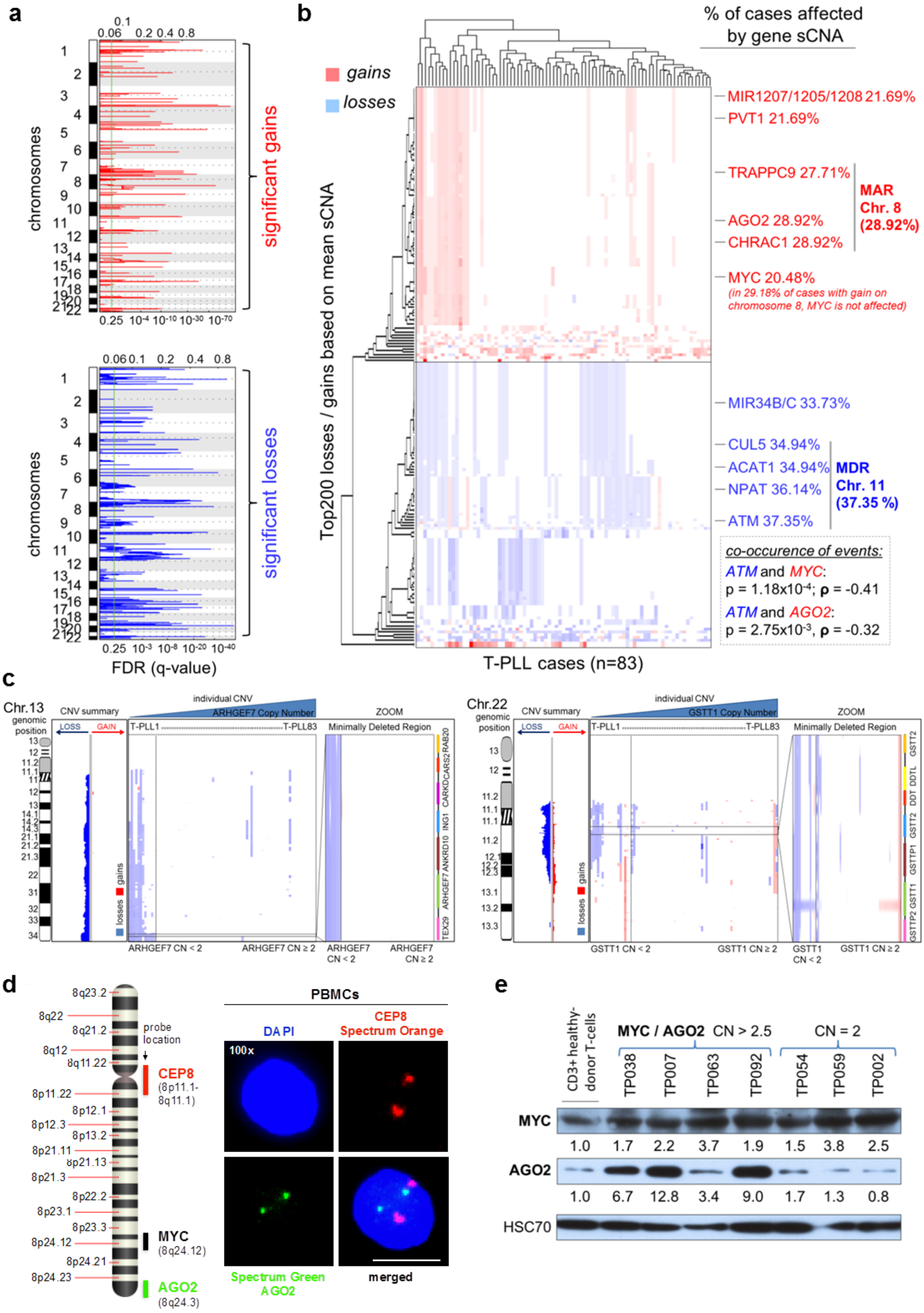

Supplementary Figure 4: Lesions identified in sCNA profiling dominantly include losses at chr.11 (*ATM*) and novel gains located on chr.8 (*AGO2*)

legend on next page

Supplementary Figure 4: Lesions identified in sCNA profiling dominantly include losses at chr.11 (*ATM*) and novel gains located on chr.8 (*AGO2*)

Globally, we identified gains (CN>2.5) in 19,590 genes and losses (CN<1.5) in 27,193 genes (**Supplementary Data 7**). The number of sCNA-affected genes (median 3354) varied inter-individually (e.g. 13,862 in TP038 *vs.* 42 in TP033).

a) GISTIC2.0 [8] analyses showing significant gains and losses in 83 T-PLL compared to 13 patient-derived normal DNAs confirmed the enrichment of lesions on chr.8 and chr.11 (compare **Supplementary Data 6** and **Fig.2b**). Among the genes that exhibit both focal gains and deletions (centers of wide peaks) at 90%-confidence level are *GSTM1* (*Glutathione S-Transferase Mu 1*; chr.1; CN=2.57) and *LCE3C* (*Late Cornified Envelope 3C*; chr.1; CN=1.64), which are also likely due to complex rearrangements.

b) Heat map showing the color-coded CN of Top200 gained / lost genes (CN mean across all T-PLL; red: CN>2.5; blue: CN<1.5). Genes characterizing the minimally amplified region (MAR) on chr.8 and the minimally deleted region (MDR) on chr.11 (see **Fig.2c**), were affected at the highest frequencies of CN events (in %; compare **Supplementary Data 7**). Chr.11 MDR: Slightly less frequently involved than *ATM* were the cell cycle factor *NPAT*, the mitochondrial acetyltransferase *ACAT1,* and the Ras ubiquitin ligase *CUL5*. Chr.8 MAR: *AGO2* is more frequently overrepresented than *MYC*.

c) MDRs on chr.13 (left) and chr.22 (right) (supplementing data to **Fig.2b,c**) showing restrictions to *GSTT1* (glutathione S-transferase theta 1, lost in 24.1% of cases) and *ANKRD10/ARHGEF7* (ankyrin repeat domain 10 / Rho guanine nucleotide exchange factor, lost in 15.7% of cases), respectively (average CN=1.91 / 1.82).

d) Verification of biallelic *AGO2* in healthy donor derived PBMCs using FISH (scale bar =5µm; control to the FISH analyses of **Fig.2d**).

e) Immunoblots on primary human T-PLL cells, n=7 cases (MYC; AGO2), and $CD3^+$ pan T-cells from PB of healthy donors. Quantifications according to HSC70 loading control via ImageJ®. Protein expression of MYC was independent of the presence of the respective sCNA lesion, e.g. showing MYC upregulation in CN-biallelic cases. Markedly elevated AGO2 expression was restricted to the cases with CN>2.5.

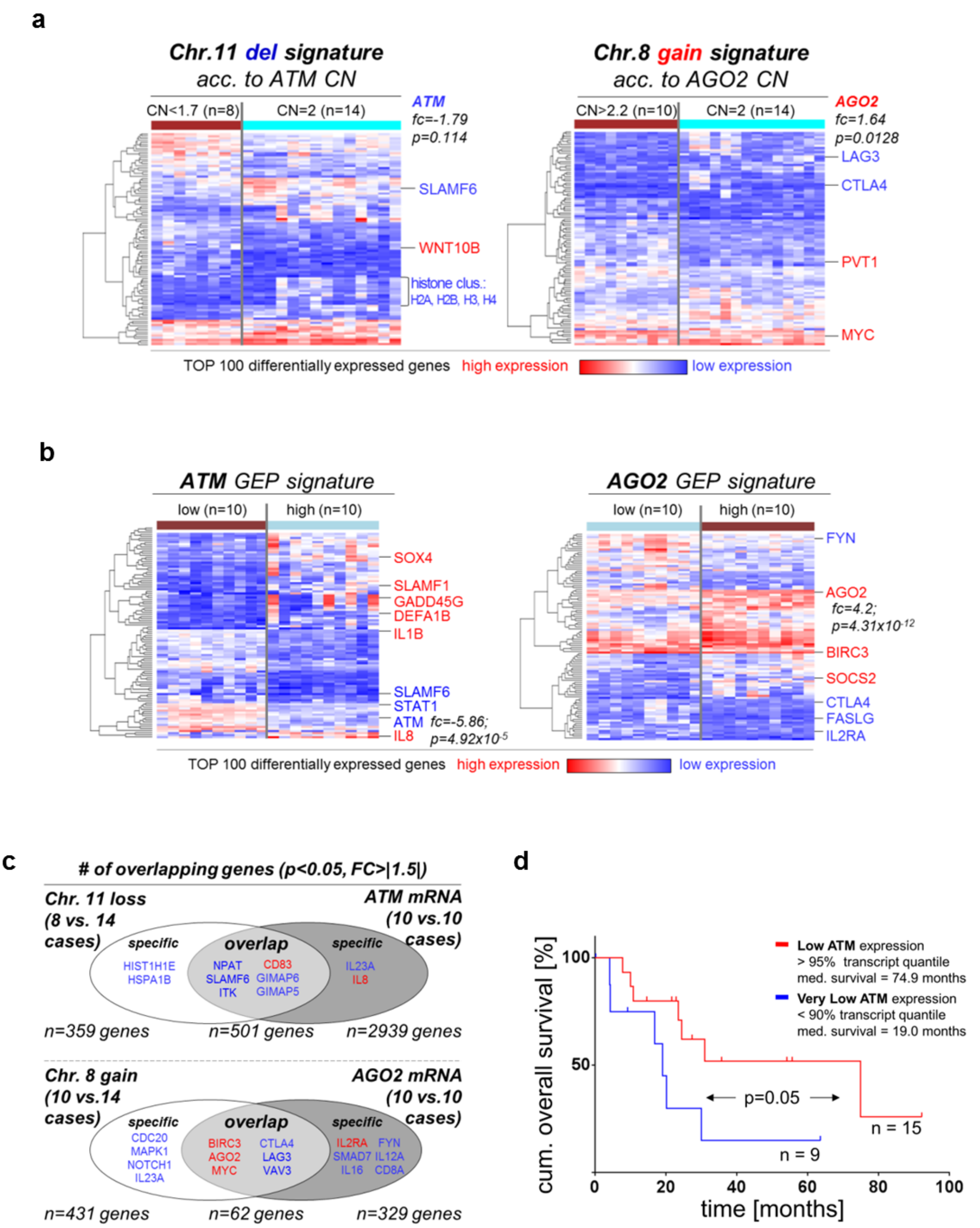

Supplementary Figure 5: Gene expression signatures associated with specific sCNAs or with cases defined by stratified expression of respectively affected genes

Despite a considerable co-occurrence of the sCNAs at chr.11 (MDR) and at chr.8 (MAR) per case (OR=3.89; p=0.002, Fisher's exact test; see **Supplementary Fig.10d** for details), there was a sizable fraction of T-PLL with discordance between the presence of these sCNAs, i.e. 35% of cases with an *ATM* loss did not harbor an *AGO2* gain.

a) Heat maps showing the differential expression (Top100) of genes specifically associated with chr.11 MDR and with chr.8 MAR. For that, GEPs of cases carrying losses at chr.11 were compared to cases 'biallelic' for chr.11 (*ATM* CN<1.7 *vs.* CN=2 according to comparison to HapMap controls; chr.8 affected cases excluded) and GEPs of cases with chr.8 gains were compared to cases 'biallelic' for chr.8 (*AGO2* CN>2.2 *vs.* CN=2 according to comparison to HapMap controls; chr.11 affected cases excluded). Among the genes that 'defined' the global differences of T-PLL cells to normal T-cells regardless of sCNA status (see **Fig.1a**) some were specifically associated with these prominent sCNAs (i.e. *SLAMF6* downregulation with presence of the chr.11 MDR and *CTLA4* downregulation with chr.8 gains (MAR); **Supplementary Data 8** for additional information). These MDRs/MARs are associated with intuitive fold-changes (fc) of expression of their defining genes, *ATM* and *AGO2*, respectively.

b) Heat maps showing the differential expression (Top100) of genes specifically associated with stratified *ATM* and *AGO2* mRNA abundance; comparison: 10 T-PLL with highest *vs.* 10 cases with lowest expression (fc of *ATM* and *AGO2* expression indicated). *AGO2* mRNA levels were significantly elevated in cases with lowest *ATM* levels (fc=1.73; p=0.02, Student´s t-test), while the generally low ATM expression is not different between AGO2 high *vs*. low cases. Interestingly, very low *ATM* mRNA levels were accompanied by an enriched deregulated expression of other DDR-associated genes, exemplified by *RAD50* or *FOXO3* (**Supplementary Data 9**).

As expected, T-PLL with lowest expression levels of *ATM* or highest expression levels of *AGO2* were enriched for those cases affected by CN lesions of the chr.11 MDR or the chr.8 MAR, respectively. *ATM*^{low} *vs. ATM*^{high}: chr.11 MDR in 8 of the 10 cases with lowest ATM expression *vs*. 1 of the 10 cases with highest ATM expression (OR=27.3; p=0.005, q=0.02). *AGO2*^{low} *vs. AGO2*^{high}: chr.8 MAR in 1 of the 10 cases with lowest AGO2 expression *vs*. 7 of the 10 cases with highest AGO2 expression (OR=0.05; p=0.02, q=0.08).

c) Gene expression signatures associated with the presence of chr.8 and chr.11 CN lesions (see **a**) were compared to those derived from stratified *ATM* and *AGO2* mRNA levels (see **b**). The GEPs of exclusively chr.11- and chr.8-affected cases appeared to be determined to a large degree by the minimal-region defining genes *ATM* and *AGO2,* based on marked overlap of GEPs: 501 of 860 differentially expressed genes associated with the chr.11 MDR are likewise associated with altered *ATM* mRNA expression; 62 of 493 differentially expressed genes associated with chr.8 aberrations are likewise associated with altered mRNA AGO2 expression. Together, both frequent sCNAs and the respectively altered expression of their defining genes (*ATM, AGO2*) are associated with unique and joint signatures, but overall with a large number of genes that displayed the most differential expression (*vs.* normal T-cells) in the entire cohort of T-PLL (not stratified by any sCNA, **Fig.1a**), i.e. *CD83, SLAMF6, GIMAPs, CTLA4, or MYC*. Overall, this highlights gene-specific and region-defined contributions to the overall GEP of T-PLL (**Supplementary Data 8, 9**).

d) Shorter overall survival of T-PLL subjects with lower *ATM* mRNA expression (GEP arrays, 5% quantile 'buffer; log-rank test).

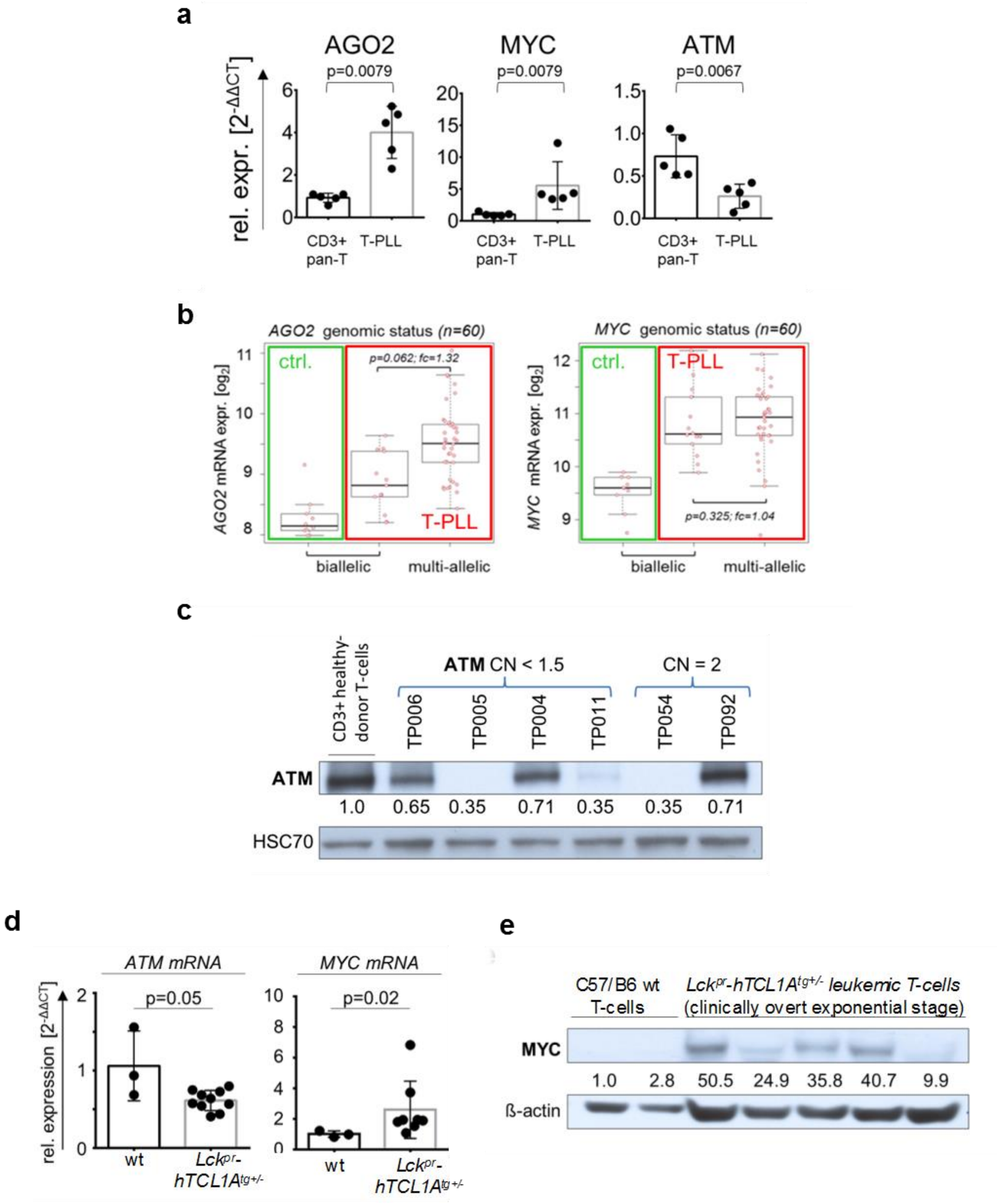

Supplementary Figure 6: Changes in transcript and protein abundance of *ATM* and *MYC* are not entirely explained by somatic CNA events on chr.11 and chr.8, respectively

a-c) Although the genes affected by the chr.11 MDR / chr.8 MAR showed decreased (*ATM*) and increased (*AGO2, MYC*) expression (array-based, qRT-PCR, immunoblots), this was rather generally disease-associated than confined to the presence of the specific genomic CN lesion (see also **Supplementary Fig.7**).

a) qRT-PCR: mRNA expression of *AGO2* and *MYC* was generally upregulated, while *ATM* expression was downregulated in primary T-PLL cells (n=5 cases) *vs.* $CD3^+$ pan

T-cells isolated from PB of healthy donors (n=5); compare GEP data in **Supplementary Data 2**.

b) mRNA expression values [log2] of *MYC* and *AGO2* derived from GEP analyses in $CD3^+$ pan T-cells isolated from healthy donors (green box), and T-PLL cases stratified as 'AGO2/MYC biallelic', and 'AGO2/MYC multiallelic' (red box) according to sCNA profiling (compare **Fig.2** and **Supplementary Data 7**). The fold-change of *AGO2* mRNA levels in *AGO2* multi-allelic over -biallelic cases was 1.32 (p=0.062, Student´s t-test; compare also with **Supplementary Fig.4e**). *MYC* mRNA expression seemed to be generally elevated in T-PLL irrespective of the presence of a *MYC* gain, pointing to additional mechanisms upregulating *MYC* expression that seem independent of genomic amplification. Boxes indicate the interquartile range (IQR). The tick line represents the mean, while whiskers reflect the lower and upper limits. Lower limit = $x_{0.25}$ - 1.5 * IQR. Upper limit = $x_{0.75}$ + 1.5 * IQR.

c) Immunoblot on human T-PLL cells, n=6, and T-cells from PB of healthy donors. Quantifications according to HSC70 loading control via ImageJ®. Protein expression of ATM was independent of the presence of the respective sCNA lesion, e.g. showing absence in CN-biallelic cases like TP054 (carried biallelic *ATM* mutations).

d & e) Murine TCL1A-driven T-PLL-like expansions generally revealed a lower sCNA abundance and recurrence than found in human T-PLL (average 70.7 sCNAs in chronic phase (n=3) and 74.8 sCNAs in exponential phase (n=5; CN<1.8 or >2.2)). **d)** qRT-PCRs of *ATM* and *MYC* mRNA in splenic T-cells of background-matched wild-type and Lck^{pr}-$hTCLA1A^{tg}$ mice reveals a downregulation of *ATM* and an upregulation of *MYC* although respective genetic CN lesions are not observed in leukemic T-cells of these animals, again pointing at CN-independent modes of deregulation (see **Supplementary Fig.2f** for cell enrichment, **Fig.1c, Supplementary Fig.2g,** and **Supplementary Data 3** for GEP derived mRNA expression levels). **e)** MYC protein expression in TCL1A-driven murine leukemic T-cell expansions: immunoblot of splenic T-cells from background- and age-matched wild-type control mice (2 T-cell pools of 3 mice each (total n=6)) and from Lck^{pr}-*hTCL1A* mice with exponential phase leukemia (for definitions see legend to **Supplementary Fig.2**, n=5) corroborated the data on upregulation of *MYC* mRNA in the usually *MYC* 'biallelic' murine leukemias (see **Supplementary Fig.2f** for cell enrichments) and paralleled the sCNA-independent MYC upregulation in human T-PLL. Quantification: ß-actin ratio via ImageJ®.

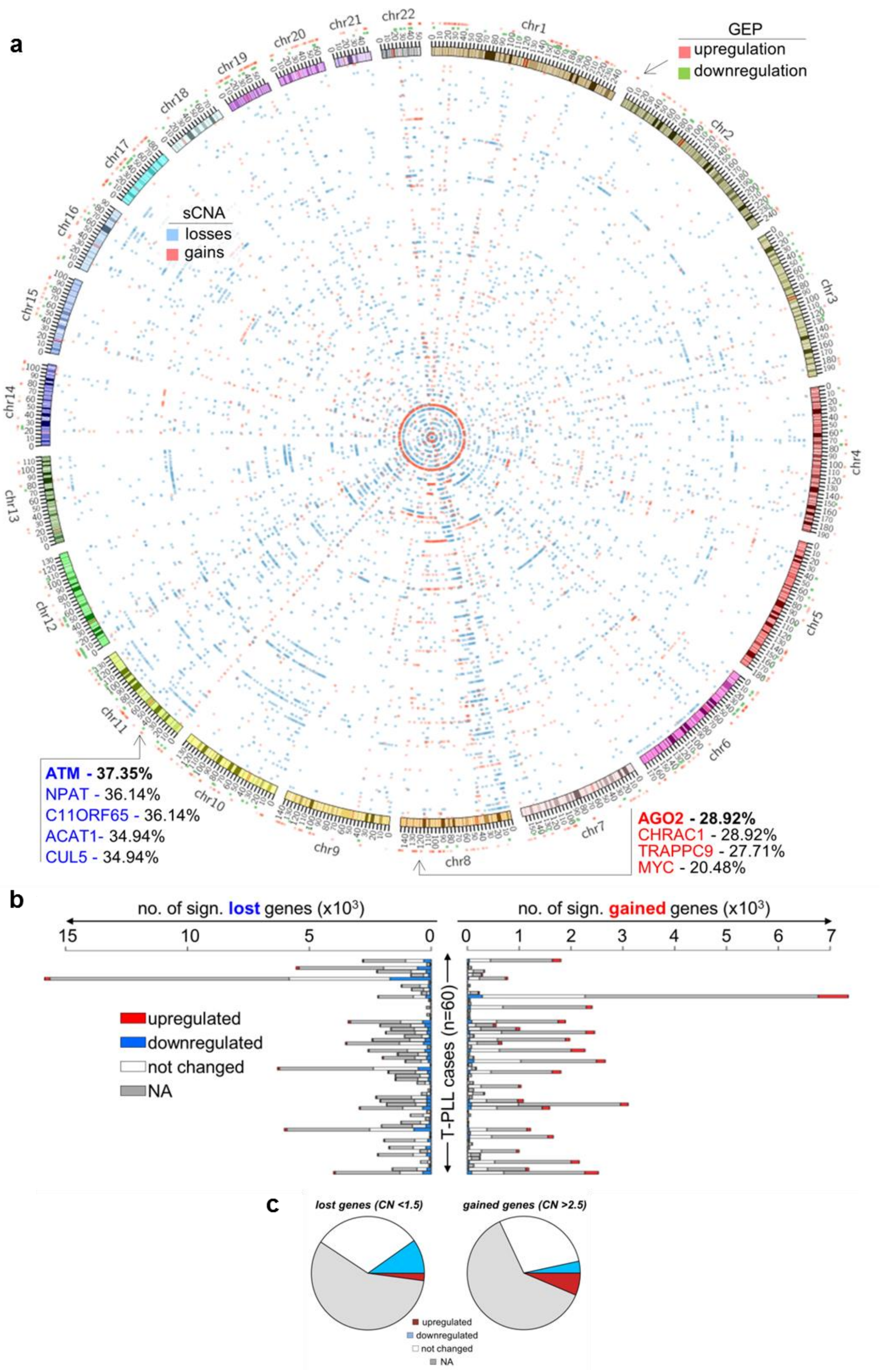

Supplementary Figure 7: Associations of large-scale genomic lesions and deregulations of global gene expression in T-PLL

legend on next page

Supplementary Figure 7: Associations of large-scale genomic lesions and deregulations of global gene expression in T-PLL

a) Circos plot mapping sCNAs and deregulations of gene expression on chromosomal loci (%: frequencies of sCNA events across entire T-PLL cohort).

b) GEPs superimposed on sCNAs with global data per case. CN lesions (exclusively monoallelic) were correlated with the differential expression of genes located in the respective regions. Although sCNA-associated changes in GEP were of generally intuitive directionality, a larger proportion of genes showed no down- / upregulation in the context of genomic losses / gains.

c) Summary of b): pie charts illustrating the association of gene-specific sCNA events with differential expression of genes. For the majority of genes, their transcript abundance remained unchanged upon losses or gains; a smaller percentage of sCNA-affected genes show an altered expression intuitively corresponding to the respective genetic change (combination of GEP and sCNA profiling data; n=60 T-PLL cases; blue: downregulated; red: upregulated; white: unchanged; grey: not annotated (N/A)). Again, all sCNA events are monoallelic.

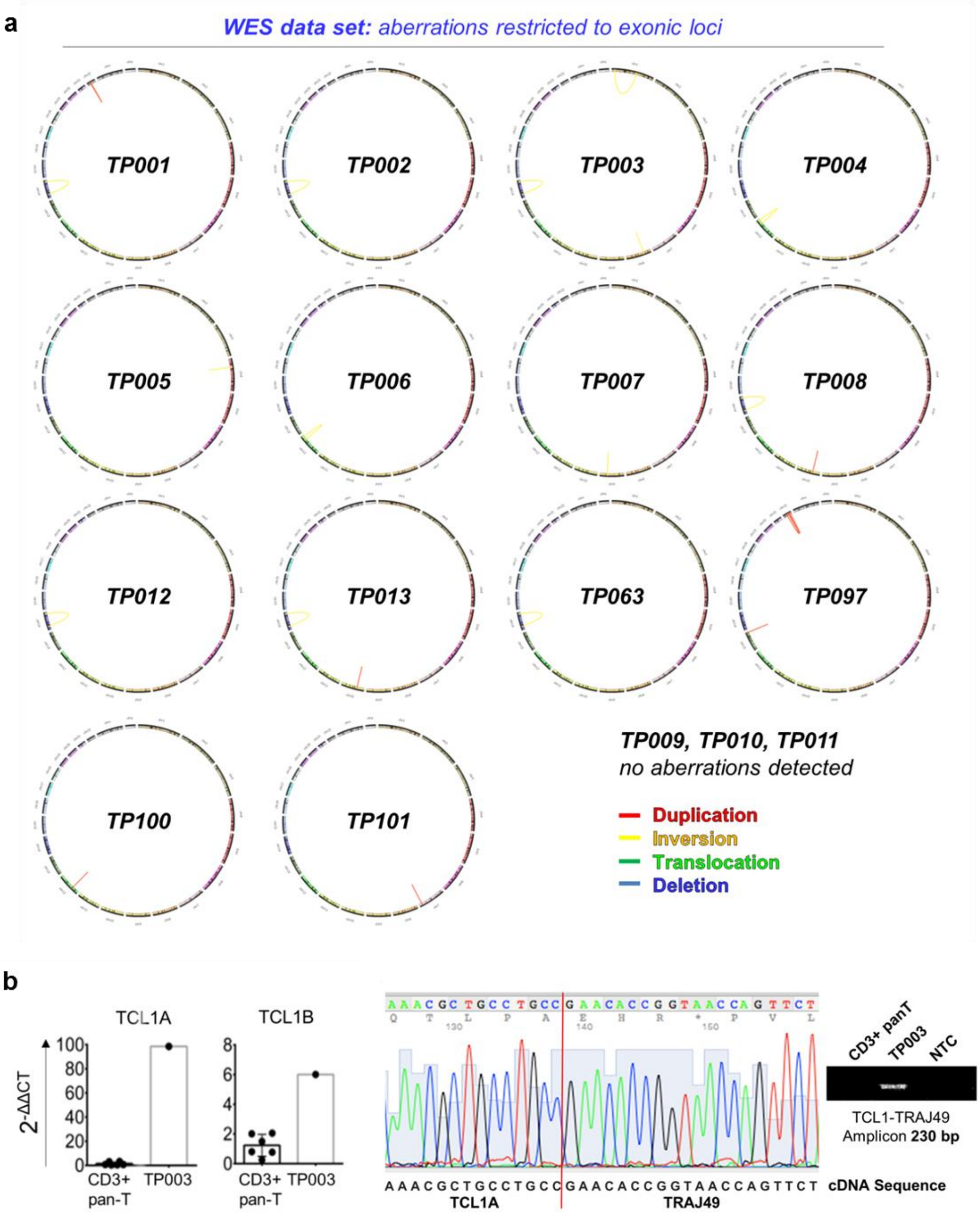

Supplementary Figure 8: Novel structural variations (SVs) in T-PLL

a) SVs (color-coded inversions / translocations / deletions) detected in exonic regions are mapped to involved chromosomal loci for all T-PLL t/g-pairs analyzed by WES. The data supplement the WGS data of **Fig.3a** (see also **Supplementary Data 10**). Based on the stringent filters applied, tandem-duplications were not detected and no SVs were detected in TP009, TP010, and TP011.

b) Left: qRT-PCR analysis showing elevated *TCL1A* and *TCL1B* transcript levels in primary T-PLL cells of case TP003 that carried the *TCL1A-TRAJ49* compared to controls (CD3$^+$ pan T-cells isolated from PB of healthy donors (n=5)). Mid: the fusion transcript was confirmed by Sanger sequencing of cDNA from TP003 (see **Fig.3b** for a schematic representation of the fusion transcript and **Figs.3c,d** for the confirmation of the genomic inv(14) and residual TCL1A protein expression). Right: Validation of the fusion transcript *TCL1A-TRAJ49* expression via RT-PCR in case TP003 compared to healthy donor derived T-cells (NTC - ’no template’ control).

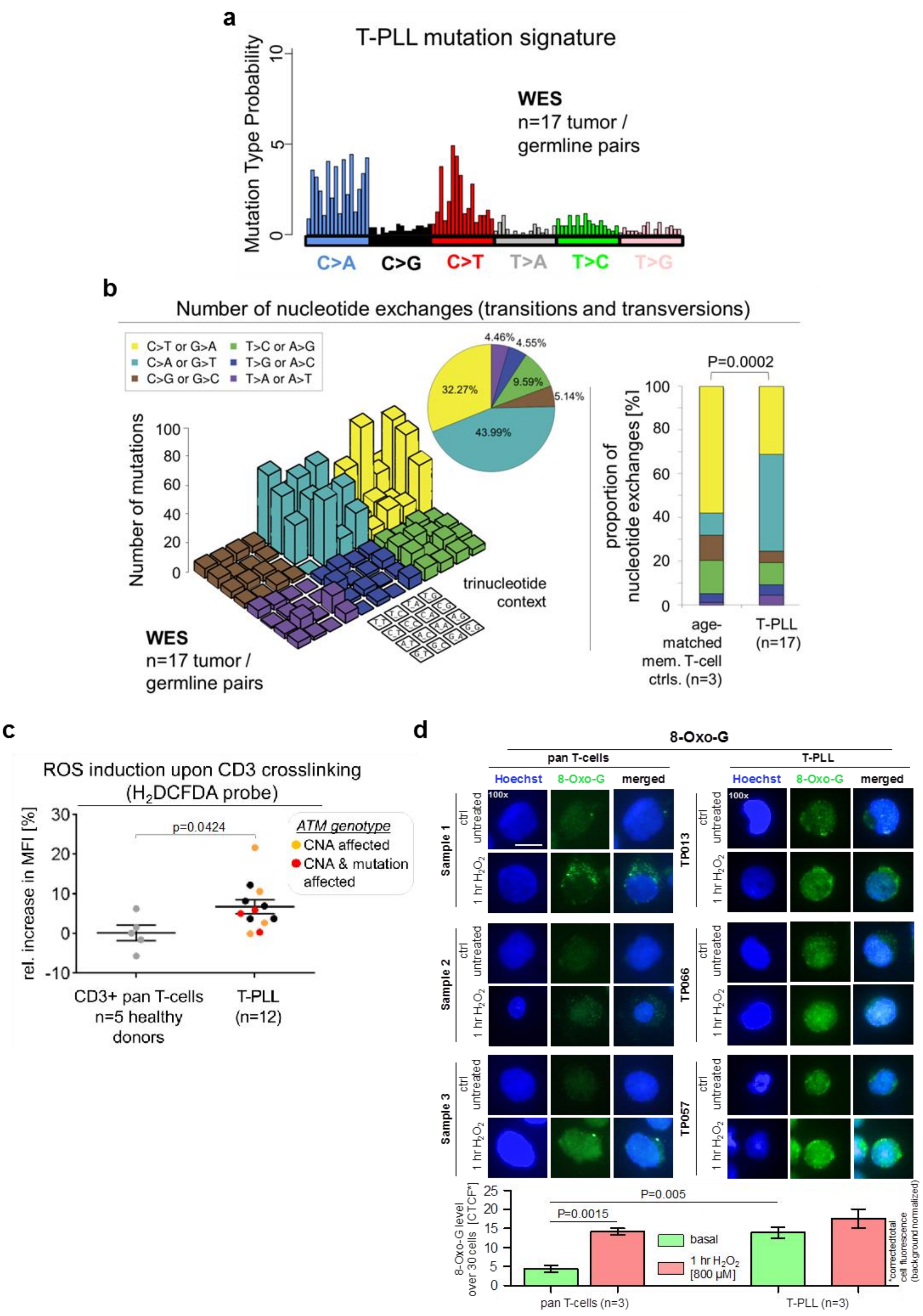

Supplementary Figure 9: Characteristics of WES detected mutations in T-PLL

legend on next page

Supplementary Figure 9: Characteristics of WES detected mutations in T-PLL

We observed a high portion of G>T and C>A transversions indicative for oxidative DNA damage (8-oxoguanine (8-oxoG)) lesions) during sample preparation. Thus, we applied additional filters similar to the ones used in *Costello et al. 2013* [9] (see **Methods** for details). Aberrations of mismatch-repair genes like short nucleotide deletions in *MSH3* of case TP002 were not associated with a generally higher number of SNVs (compare **Fig.4b**), base-exchange preferences, differences in mutation rates by loci, or microsatellite instability. **a)** Mutation signature of T-PLL according to Alexandrov and colleagues[10] derived from WES data of 17 t/g-matched cases. Comparing the T-PLL specific signature to previously catalogued cancer genomes, the Top5 significantly correlated signatures (Spearman correlations) were: signature 29 ('tobacco chewing'; $\rho=0.69$; $p=7.07\times10^{-15}$), signature 1 ('aging'; $\rho=0.65$; $p=8.86\times10^{-13}$), signature 18 ('neuroblastoma'; $\rho=0.63$; $p=4.37\times10^{-12}$), signature 14 ('smoking'; $\rho=0.62$; $p=2.13\times10^{-11}$), and signature 19 ('pilocytic astrocytoma'; $\rho=0.59$; $p=1.21\times10^{-10}$). For a visualization of those signatures please refer to http://cancer.sanger.ac.uk/cosmic/signatures. **b)** Left: Lego plot of SNV (PopFreq<0.01 or COSMIC-annotated, 8-OxoG corrected) frequencies with trinucleotide context and overall percentages in pie chart / stacked columns. C:G>A:T (44%) and C:G>T:A (32.3%) transversions represent the largest portions of exchanges observed in a di-thymidine (T_T) context. Right: The global distribution of SNV frequencies is significantly different comparing suggestive 'T-cell-aging acquired' *vs.* 'tumor-associated' patterns of mutations ($p=0.2\times10^{-3}$, Wilcoxon test). The WES data of isolated memory T-cells from age-matched healthy donors (n=3; ages 61, 63, 65 years) were compared to memory T-cell subsets of young donors (ages 22, 28, 31 years). Note that the apparent discrepancy of frequency-ranks compared to **Fig.4c** is explained by the illustration of medians in main **Fig.4c** whereas an illustration of overall frequencies is given here. **c)** 2',7'-dichlorodihydrofluorescein diacetate (H_2DCFDA) based measurements of reactive oxygen species (ROS) induction upon T-cell receptor (TCR) activation comparing healthy T-cells (grey dots) to primary T-PLL cases (mean with SEM). *ATM* genetic status: orange - CN<1.5, no mutation; red - CN<1.5, mutated; black dots - no genomic ATM status available). Although ROS induction upon CD3/CD28 crosslinking seems to be independent of the presence of an *ATM* sCNA/mutation, there was a generally higher increase of ROS levels in stimulated T-PLL cells compared to $CD3^+$ pan T-cells isolated from PB of healthy donors. This observation might be linked (1) to a sub-standard performance of the ROS attenuator ATM in T-PLL, (2) to the TCR-sensitizer function of TCL1A[11], (3) to TCL1A's effect on mitochondrial ROS generation[12] or (4) to other aberrancies such as inefficient buffer systems. It fits also well with the relative increase of C:G>A:T exchanges observed among all WES-detected SNVs (compare **Supplementary Fig.9b**), which can specifically result from ROS induced DNA damage[13]. **d)** Cellular 8-oxoG evaluated by IF staining and microscopy with an 8-oxoG specific antibody. Basal levels are higher in primary T-PLL cells (right panel) than in healthy-donor derived pan-T-cells (left panel; p=0.005, Student´s t-test; data supplementing **Fig.4c**). Whole-cell signals (nuclear and mitochondrial, scale bar =5µm) of 8-oxoG were quantified as CTCF (corrected total cell fluorescence) values. Specificity is shown by increased CTCF values upon 1hr treatment with H_2O_2 (p=0.0015, Student´s t-test).

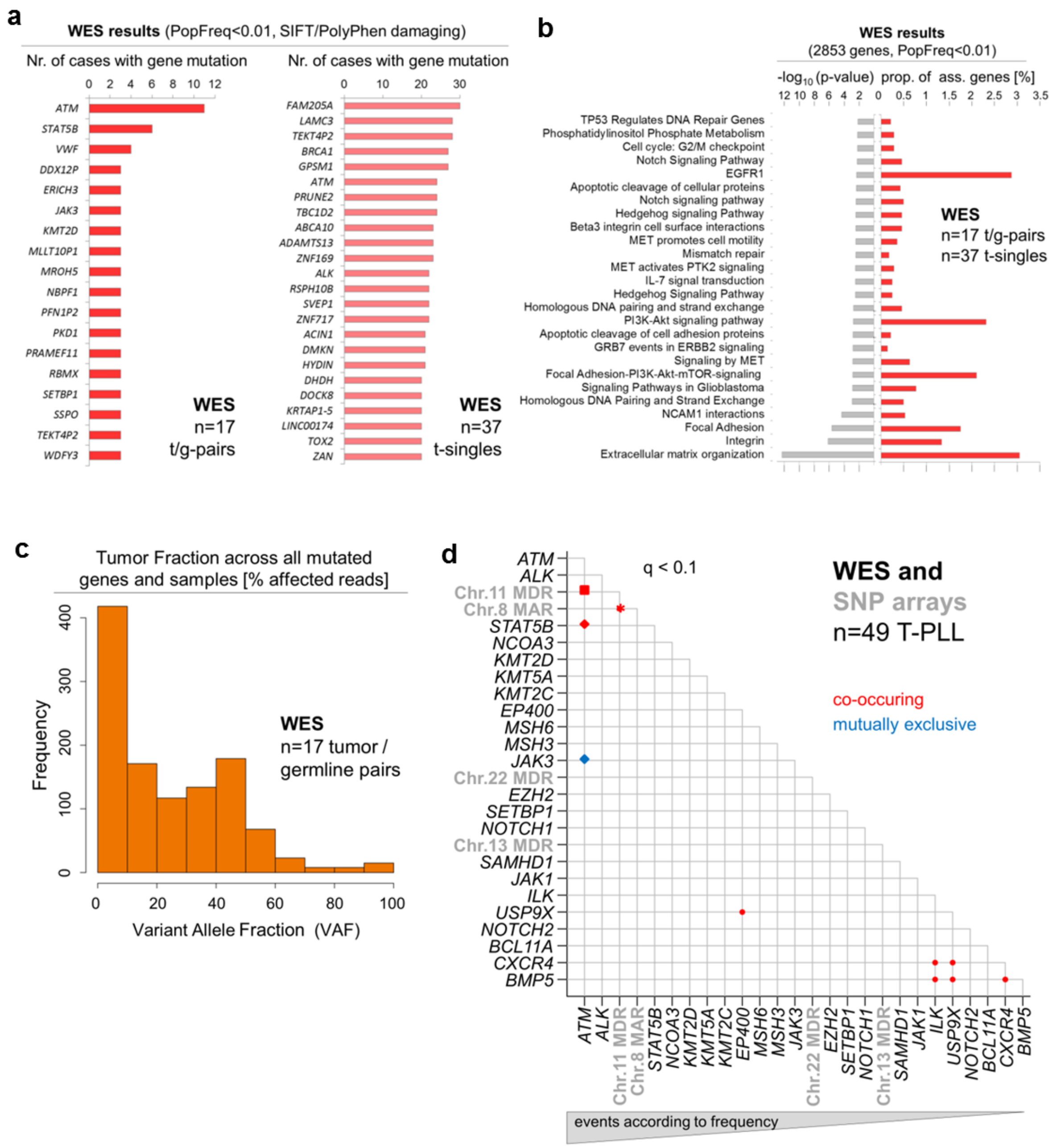

Supplementary Figure 10: Functional affiliations and correlations of highly frequent variants

a) Lists of genes recurrently mutated with highest frequencies across all analyzed t/g-pairs and t-singles (only SIFT[14]/PolyPhen2[15] and PopFreq-filtered or COSMIC-annotated mutations included; compare **Supplementary Data 12** and **Fig.4d** for a selection of functionally annotated genes).

b) GSOA from 2853 genes harboring mutations in exonic regions (PopFreq<0.01) as identified in t/g-pairs and t-singles revealed an overrepresentation of those involved in p53 mediated DDR, cell cycle regulation, apoptosis, and interleukin signaling. Proportion of genes [%] associated with the respective process in relation to the total number of mutated genes (red bars) and specific p-values (grey bars) are given.

c) Tumor fractions (variant allele fractions, VAFs) of all identified mutations detected in WES data (% positive reads) according to their overall frequencies (Y-axis). The

incidence of mutations showing a 'high clonality' (80-100% tumor fraction) was rather low (1.4% of all mutations) pointing to a small number of drivers compared to a high number of subclonal passenger mutations; e.g. 36.6% of all mutated genes have VAFs of ≤10% (see also **Fig.4e** and **Supplementary Data 12** for tumor fractions of specific genes).

d) Correlations of most frequent genomic events were calculated based on distinct datasets. They were embedded in a primary q-value-matrix with correlations (circle) estimated by Fisher's exact test with Benjamini-Hochberg FDRs derived from the 49 T-PLL representing the overlap between cases analyzed by SNP arrays and WES. Star: Associations among sCNAs in the dataset of all 83 SNP-array analyzed T-PLL revealed a significant co-occurrence of the presence of a chr.8 MAR with the detection of a chr.11 MDR (OR=3.89; p=0.002, Fisher's exact test). The case distribution was as follows: only chr.8 MAR in 10 cases (12%); exclusively chr.11 MDR in 21 cases (25%); co-occurrence of both lesions in 23 cases (28%); none of both lesions in 29 cases (35%). Thus, when present (54 cases) these lesions coincide in 42.6% of such cases and are mutually exclusive in 57% of such cases. Square: The LOH relationship of *ATM* mutations with presence of a chr.11 MDR (mono-allelic *ATM* deletion) within our data set including TAS und further cytogenetic information (72 cases due to increased platforms overlap) is reflected by a significant co-occurrence of both lesions (OR=4.33; p=0.0046, Fisher's exact test). Diamond: Among the somatic mutations, we additionally identified a significant co-occurrence of lesions in *STAT5B* and *ATM,* when using the MuSic mutation relation analysis and subsequent testing (OR=4.5; p=0.045, Fisher's exact test). In the extended set of WES cases (n=54), a biased presence of *JAK3* variants in *ATM* unmutated cases was associated with an OR=0.41 for *JAK3* mutations and *ATM* mutations (p=0.098, q<0.1, Fisher's exact test).

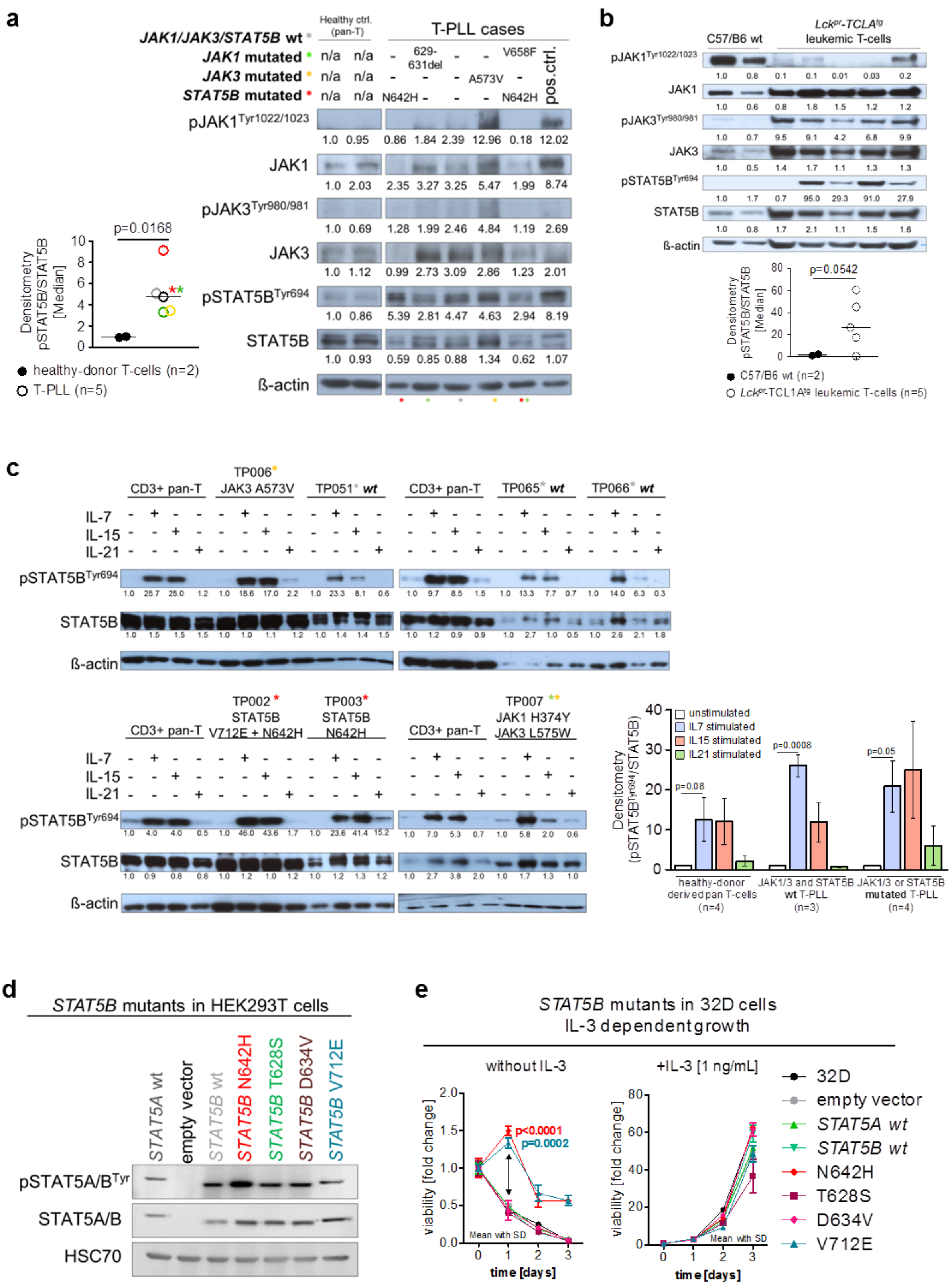

Supplementary Figure 11: Genomic alterations in *JAK/STAT* signaling pathway components do not predict basal and interleukin-induced JAK/STAT phospho-activation levels, but confer specific pro-survival effects

legend on next page

Supplementary Figure 11: Genomic alterations in *JAK/STAT* signaling pathway components do not predict basal and interleukin-induced JAK/STAT phospho-activation levels, but confer specific pro-survival effects

a) Immunoblot analysis showing protein levels with basal phosphorylation (activating motifs) of JAK1, JAK3, and STAT5B in primary T-PLL cells (5 cases) with known respective gene mutation status. No obvious association of analyzed basal phospho-activation levels with the presence of a respective mutation. Controls: $CD3^+$ pan T-cells isolated from PB of healthy donors (n=2). Lysates from IL-2 stimulated HH cells represent positive controls. Quantification of immunoblots: ImageJ®, represented as bar charts (Student’s t-test).

b) Immunoblots for protein levels with phospho-activation status of murine JAK1, JAK3, and STAT5B motifs (cross-species reactivity of the antibody) in primary splenic T-cells of Lck^{pr}*-hTCL1A* transgenic mice (overt exponential phase, n=5). Controls: splenic T-cells of genetic-background and age-matched wild-type animals (2 batches as pools of T-cell isolates from 6 animals (3 each)). Quantifications of immunoblots: ImageJ®, bar charts, Student’s t-test with n=2 for control T-cells.

c) To model micromilieu mediated conditions via cytokine signaling, healthy donor derived pan-T-cells (n=4) and primary T-PLL cells with known *JAK1/JAK3/STAT5B* mutation status (n=7) were exposed *ex vivo* to IL-7, IL-15, and IL-21. Activation was recorded via phospho-$STAT5B^{Tyr694}$ immunoblots. Quantification: ImageJ®, represented as bar charts, fold-induction upon IL-7 stimulation was compared based on densitometric quantifications (healthy-donor derived pan-T-cells: 12.6 fold, p=0.08; *JAK1/3* and *STAT5B* wt T-PLL: 20.9 fold, p=0.0008; *JAK1/3* and *STAT5B* mutated T-PLL: 20.8 fold, p=0.05, Student’s t-tests). The extent of *ex vivo* cytokine-mediated pSTAT5B induction in *JAK/STAT* mutated T-PLL does not differ from the one in unmutated cases (IL7: p=0.54, IL15: p=0.42, IL21: p=0.41, Student’s t-tests).

d) Ectopic expression of *STAT5B* mutants in HEK295T cells. Expression and phosphorylation of STAT5B via immunoblot (n=2 biological replicates; one representative experiment shown).

e) Growth of 32D hematopoetic precursor cells and their altered IL-3 dependence upon expression of *STAT5B* mutants (mean with SEM; p-values relative to the *STAT5B* wt condition; Student´s t-test); n=2 biological replicates with 4 technical replicates each.

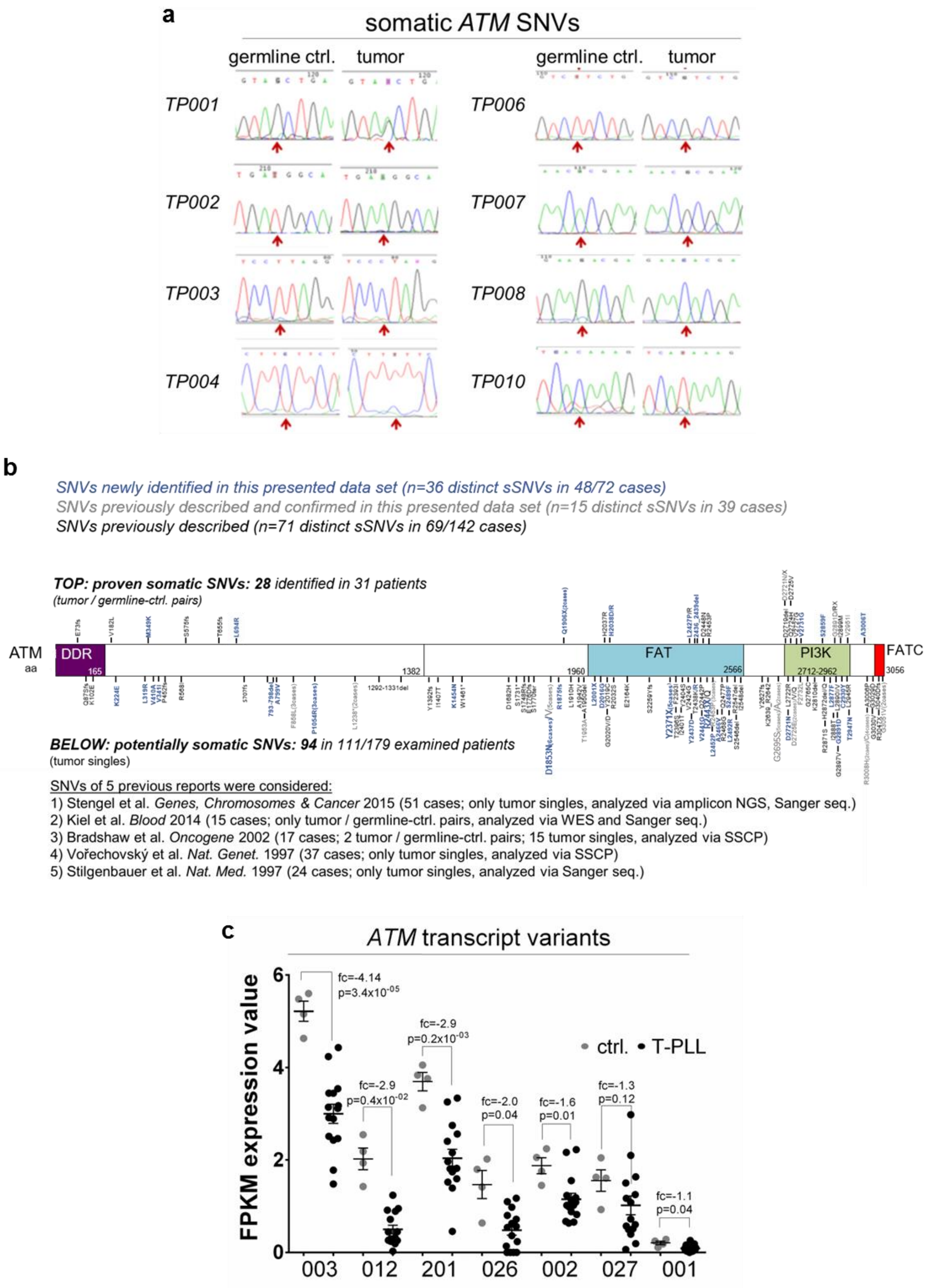

Supplementary Figure 12: Validations of *ATM* somatic mutations and clustering of *ATM* SNVs in the FAT and PI3K domains

legend on next page

Supplementary Figure 12: Validations of *ATM* somatic mutations and clustering of *ATM* SNVs in the FAT and PI3K domains

a) *ATM* mutations detected in t/g-pairs by WES are validated by Sanger sequencing. Eight distinct exemplary SNVs were confirmed as somatic mutations present in 8 T-PLL (compare **Supplementary Data 12**).

b) Scheme of the ATM molecule with mapping of mutations identified by WES, TAS, and Sanger sequencing (i) according to their description in this series *vs.* previous publications[16–20] (all published data sets carrying sequencing data on *ATM* in T-PLL were selected) and (ii) according to their calling from t/g-pairs (proven somatic, top) *vs.* from tumor singles (potentially somatic, bottom). A clustering in the FAT and PI3K domains (29.2% and 21.4% of all mutations, respectively) is revealed. Compare **Fig.5a** for a scheme showing *ATM* SNVs only identified as part of this study. A dominant missense character of mutations is described, unlike the dominant truncating mutations identified in *A-T* individuals[21].

c) Differential expression of variant *ATM* transcripts (WTS analysis) in T-PLL (n=15) compared to healthy-donor $CD3^+$ T-cells ('ctrl.', n=4). Confirmed downregulation of 5/7 protein coding *ATM* variants in T-PLL (fc>|1.5|); those not differentially expressed are expressed at generally low levels in both, ctrl. and T-PLL (mean with SEM; p-values as per Student´s t-test).

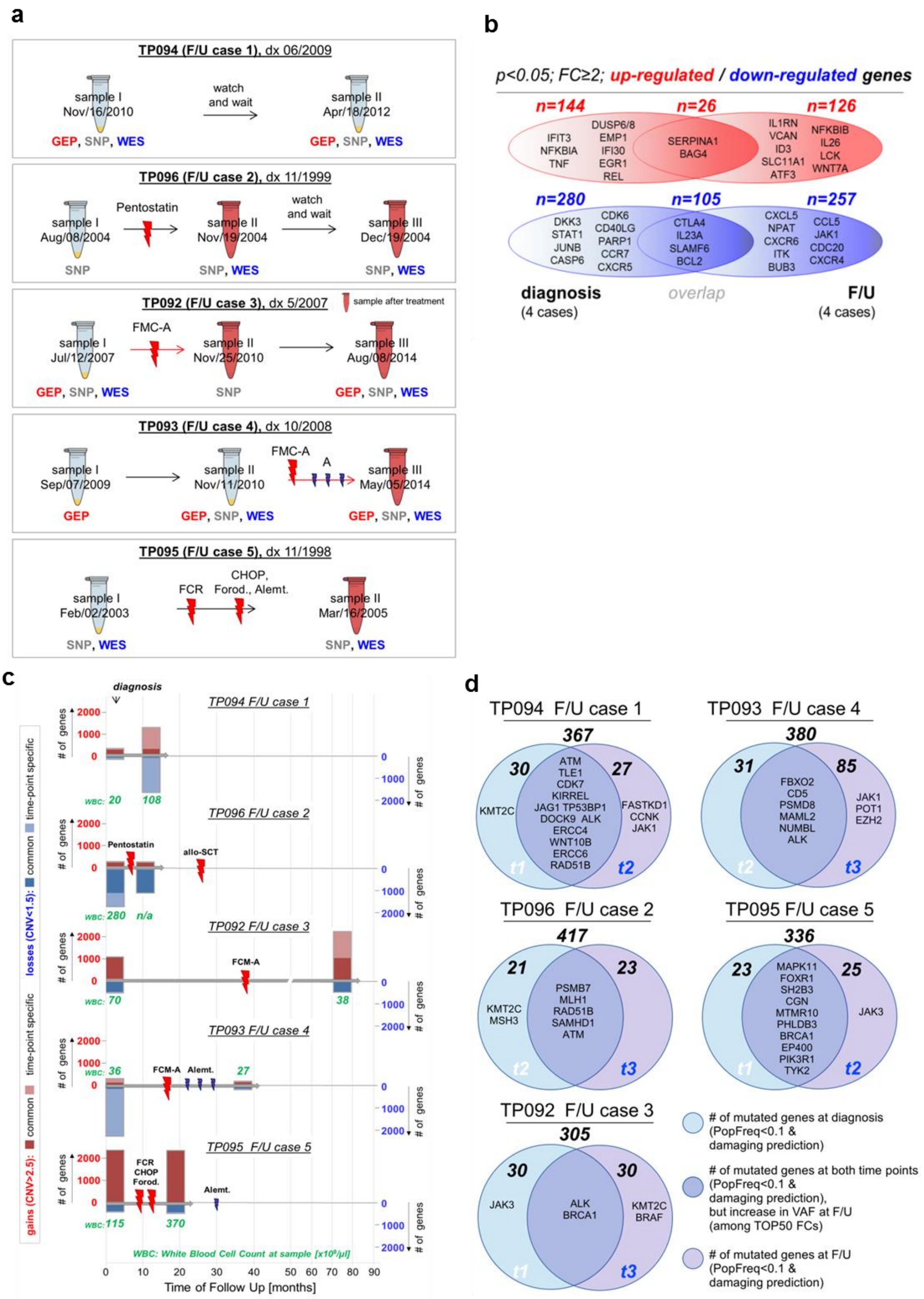

Supplementary Figure 13: Analysis of sequential follow-up (F/U) samples

legend on next page

Supplementary Figure 13: Analysis of sequential follow-up (F/U) samples

a) Among the total of 72 T-PLL analyzed on sequencing platforms, sequential samples were available for n=5 cases with sufficiently long F/U (13 samples). The median total F/U time for all cases was 24 months (range 5 - 85) and the median of sample intervals was 20.5 months. The first samples, close to initial diagnosis (treatment naïve), were followed by those after clinically relevant progression or relapse after therapy. These samples were analyzed by at least one of the profiling approaches: GEP, SNP-arrays (for sCNAs), and WES. For F/U case 1, one second sample was collected after 17 months. In F/U case 2, within 8 months 3 samples were collected and analyzed via sCNA profiling and WES. In F/U case 3, 3 sequential samples were collected over a long course of 95 months and subjected to GEP, sCNA profiling, and WES. This patient received an FMC-A chemo-immunotherapy (fludarabine, mitoxantrone, cyclophosphamide; followed by alemtuzumab) between 1st and 2nd sampling. F/U case 4: over 56 months, 3 samples were collected and analyzed via GEP, sCNA profiling, and WES. F/U case 5: 2 sequential samples within 24 months. This patient was heavily treated in-between with distinct chemo-immunotherapies: FCR (fludarabine, cyclophosphamide, rituximab), CHOP (cyclophosphamide, doxorubicine, vincristine, and prednisone), forodesine, and single-agent alemtuzumab. Here, sCNA profiling and WES were performed.

b) GEP of 4 cases with available F/U sample pairs. Differential expression calculated separately for each time point (*vs.* healthy-donor T-cells). Selection from lists of differentially up- (red) and down-regulated (blue) genes at each time-point or with overlap (**Supplementary Data 16**). The majority of transcripts were specifically restricted to either time-point. We furthermore identified an increase in *TCL1A* mRNA levels (fc^{t1}=4.24, p=0.09 *vs.* fc^{t2}=11.34, p=0.03, Student’s t-test) in all cases. The genes with unchanged dysregulated mRNA levels were frequently those that most significantly contributed to the difference of T-PLL to normal T-cells, i.e. *CTLA4*, *SLAMF6* (**Fig.1**).

c) Total numbers of genes affected by sCNAs (gains=red / losses=blue) plotted for the 5 F/U pairs. The difference of global sCNAs between t1 (2402 events at diagno-sis) and the time of progression / relapse (3356 events) is quantified by 1.4-fold (p=0.06, Wilcoxon test). Treatments and leukocyte counts at sampling are indicated (also **Supplementary Data 17**).

d) WES of 5 F/U cases: Mutated genes at first *vs.* second time-point were compared; a selection of affected genes is highlighted and overall numbers provided. See also **Supplementary Data 18** and **Fig.5c** for dynamics of dominant clusters and their lead variants.

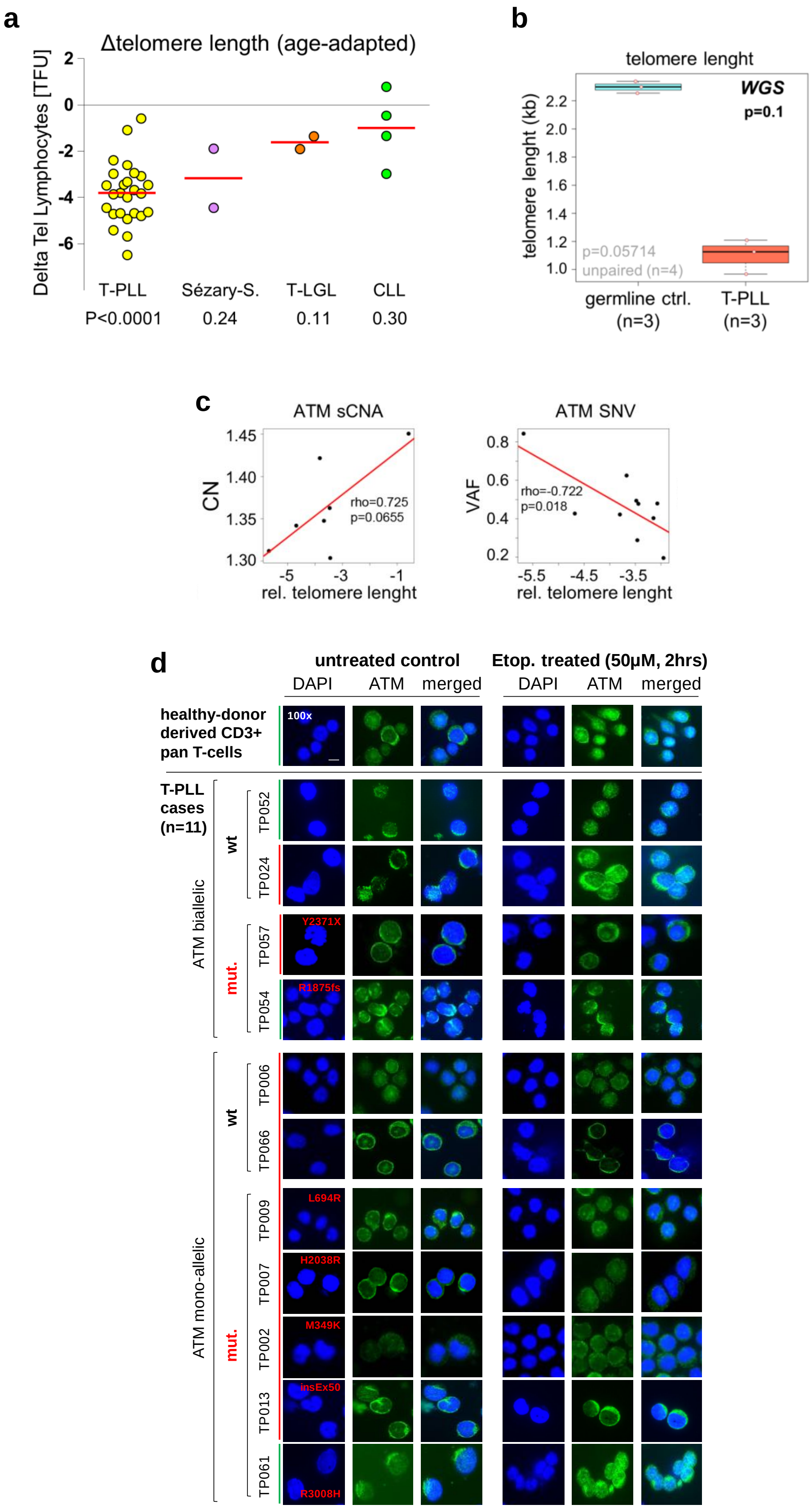

Supplementary Figure 14: Primary T-PLL cells show a phenotype of severe telomere attrition and impaired ATM nuclear translocalization upon DNA damage

legend on next page

Supplementary Figure 14: Primary T-PLL cells show a phenotype of severe telomere attrition and impaired ATM nuclear translocalization upon DNA damage

a) Telomere lengths in 26 T-PLL cases (compare to **Fig.7a** for an age-adjusted depiction) as well as 4 CLL, 2 T-LGL, and 2 cases of Sézary Syndrome. Measurements were done by flow-FISH and healthy controls were used for age-adaptation as described previously[22]. One telomere fluorescence unit (TFU) corresponds to 1 kilobase pair(s). The data confirm indications of particularly short telomeres in T-PLL in a previous smaller series[23].

b) Telomere lengths were evaluated according to WGS data using the 'telseq'[24] algorithm. The difference between tumor and germline samples (n=3 paired WGS data sets and the 1 WGS tumor 'single' included) was not statistically significant (p=0.1 in Wilcoxon paired test; p=0.06 in unpaired; consider small sample size). Boxes represent the interquartile range (IQR); thick line reflects the mean; whiskers indicate the lower and upper limits. Lower limit = $x_{0.25}$ - 1.5 * IQR and upper limit = $x_{0.75}$ + 1.5 * IQR.

c) Association of telomere lengths (flow-FISH) with *ATM* lesions (sCNAs and sSNVs): showing shorter ends in cases with low *ATM* CNs and high *ATM* VAFs.

d) Subcellular ATM localization in IF microscopy of cytospins of untreated *vs.* Etoposide-treated primary T-PLL cells and PBMC controls (scale bar =5µm; supplementary data to **Fig.7b**; here all analyzed cases). IF panel: Only 3 of 11 cases (green marks) show a predominant nuclear translocalization of ATM upon DSB induction comparable to healthy-donor PBMCs (one representative example of 3 experiments shown). Among cases with regular ATM subcellular kinetics, one harbored an ATM-biallelic / unmutated constellation, one had an ATM biallelic genotype with a mutation (R1875fs), and one an ATM-monoallelic genotype with a mutation in the FATC domain (R3008H). Seven of the 8 cases without proper ATM translocalization (red marks) had affected, but heterogeneous, *ATM* genotypes.

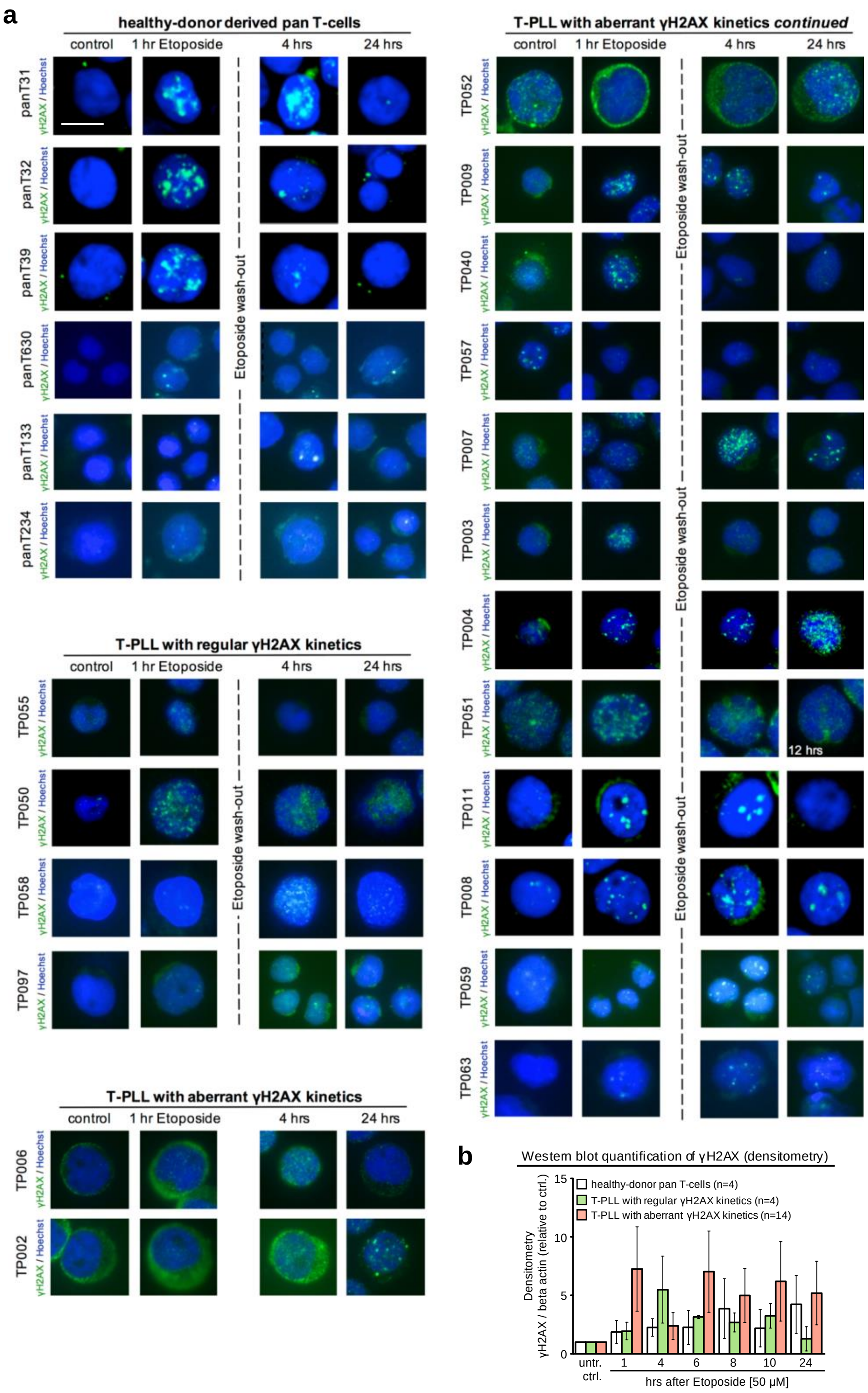

Supplementary Figure 15: γH2AX focus induction and removal in a set of 23 primary T-PLL analyzed via immunofluorescence microscopy and / or immunoblot

a) Entire set of 18 T-PLL analyzed by γH2AX immunofluorescence (IF) microscopy. Overlap with Western blot data (b) towards 23 cases in total. The data supplement **Fig.7c**. Six healthy-donor T-cell samples served as controls (scale bar =5µm).

b) Summary of densitometries from immunoblots of n=18 cases *vs.* 4 T-cell controls (mean with SEM).

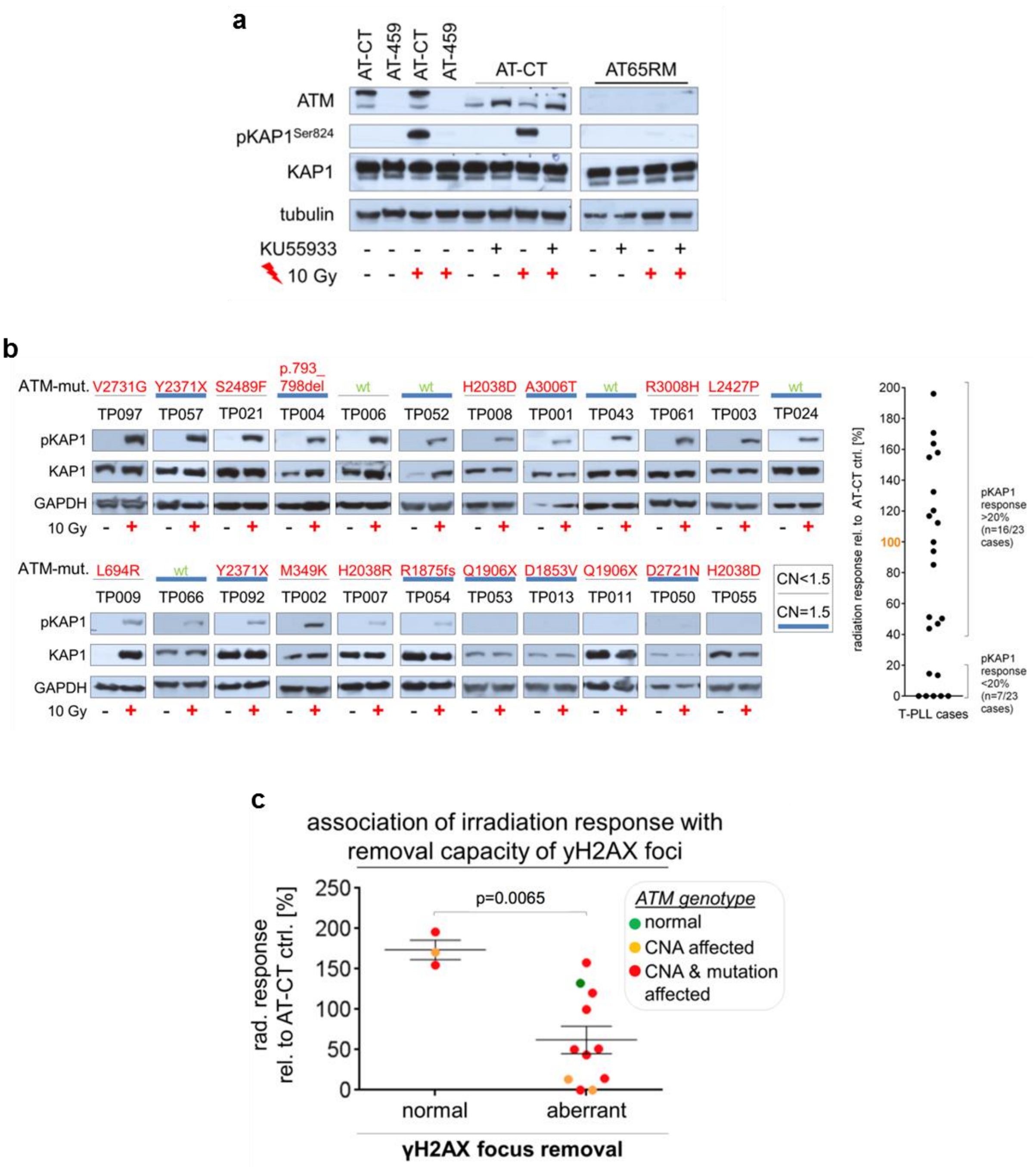

Supplementary Figure 16: ATM in primary T-PLL cells is hypomorphic as per canonical effector functions

a) Control system for activation of the ATM target KAP1 (see **b**): lymphoblastoid B-cell lines from *A-T* patients[25] (AT65RM, $ATM^{\Delta/\Delta}$, c.6573-9G->A/ c.8814_8824del11, ATM protein absent) or unaffected relatives (AT-CT, ATM^{wt}) were pretreated with the ATM kinase inhibitor KU55933 at 50µM for 2hrs. Cells were then exposed to 10Gy ionizing irradiation (IR) and pKAP1^{Ser824} levels were detected 1hr thereafter by Western blot. IR-induced phosphorylation of KAP1 is only detectable in ATM wt cells without KU55933 treatment underlining the specificity of ATM mediated KAP1 phosphorylation.

b) KAP1^{Ser824} phosphorylation upon 10Gy IR was assessed in primary T-PLL cells of 23 cases. Note that separation of lanes in the presentation of Western blot data was done in order to better assemble cases according to their pKAP1 response levels. Overall, the bulk of cases showed residual pKAP1 induction, despite genomic *ATM* lesions; some (9/23 cases) even above the levels (100%-mark) seen in AT-CT control cells (densitometry data on right). T-PLL with *ATM* in CN-biallelic / SNV-wt constellation usually revealed IR-induced KAP1 phospho-activation, while the rare T-PLL with truncating mutations (TP011; Q1906*) or some few cases with CN monoallelic / ATM mutated status (TP055) did not. Quantification of IR response by densitometry of immunoblots: the levels of pKAP^{Ser824} protein relative to pan-KAP1 and housekeeping controls were normalized to induced pKAP1^{Ser824} levels in the AT-CT control cell line (set to 100%).

c) There is a correlation of the capacity to phosphorylate KAP1 upon IR (mean with SEM) with the capacity to induce / remove yH2AX foci following etoposide treatment (see **Fig.7c**). Cases with regular biochemical IR responses (pKAP1) show normal yH2AX kinetics. More than half of cases with abnormal yH2AX platform induction / resolution show reduced pKAP1^{Ser824} responses.

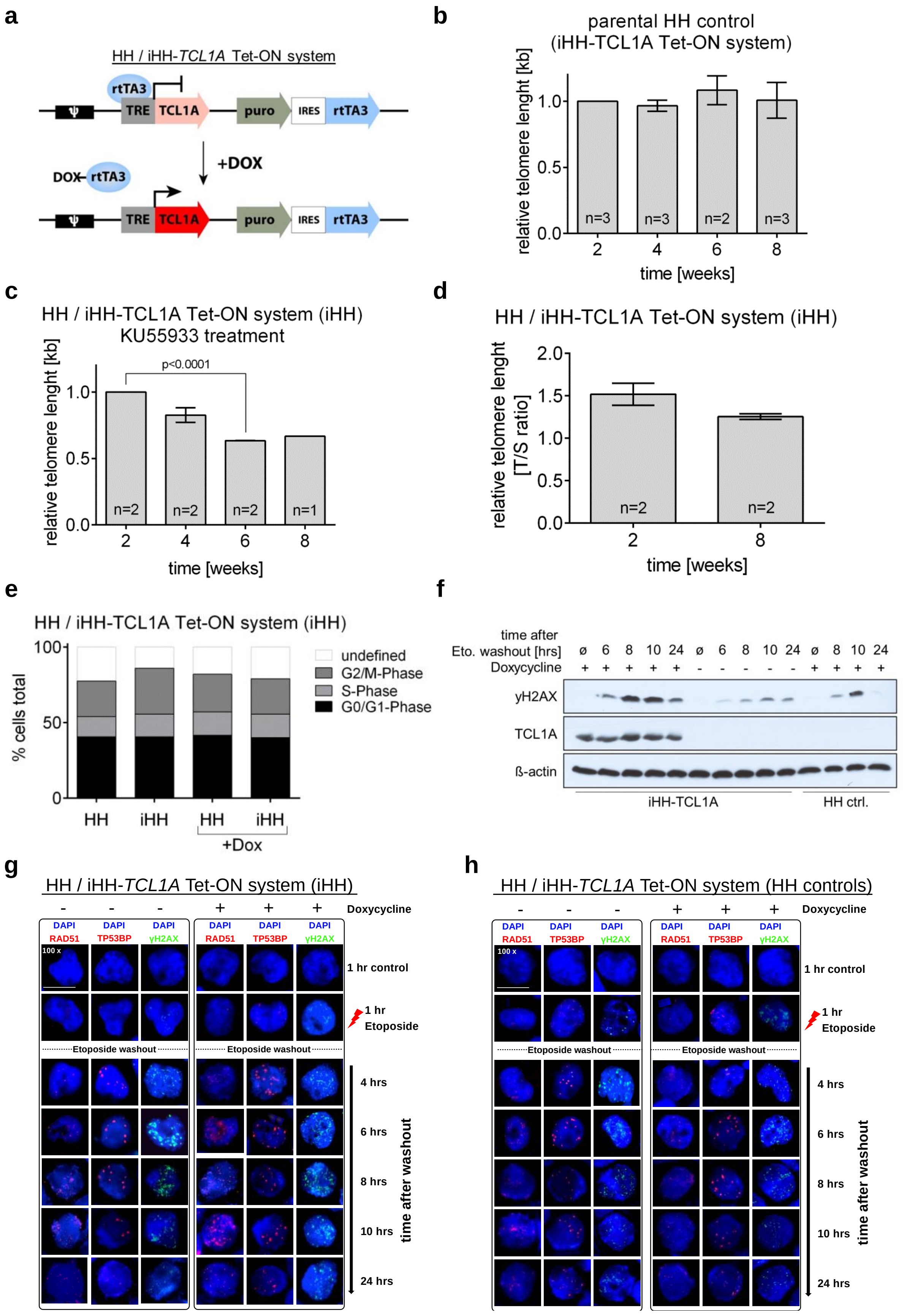

Supplementary Figure 17: Ectopic expression of *TCL1A* affects telomere maintenance and the yH2AX mediated DDR

legend on next page

Supplementary Figure 17: Ectopic expression of *TCL1A* affects telomere maintenance and the yH2AX mediated DDR (Supplements to **Fig.8**)

a) Schematic representation of the *TCL1A* expression vector stably transfected in HH mature T-cell leukemia cells (resulting line 'iHH'). TRE: tetracycline responsive element; Puromycin: Puromycin resistance cassette; IRES: internal ribosomal entry site; rtTA3: reverse tet-transactivator 3. Inducible *TCL1A* expression: upon Doxycycline (Dox) treatment, release of the transactivator protein from *TCL1A* promoter binding results in induction of *TCL1A* transcription.

b-d) Telomere length measurements (mean with SEM, supplementary data to **Fig.8a**). **b&c)** Flow-FISH data: Doxycycline treatment of parental HH cells does not affect telomere lengths over a time-course of 8 weeks; telomere shortening in response to pharmacological ATM inhibition (63.3% after 6 weeks; $p<0.0001$, Student´s t-test). **d)** qRT-PCR based validations of telomere length reduction in Doxycycline treated (TCL1A expressing) iHH cells (early *vs.* late time point) using published protocols[26].

e) iHH-TCL1A cells and HH parental controls were treated with Doxycycline for 24hrs (1µg/ml). Cell cycle profiles, determined by DNA content assessments using propidium-iodide based flow-cytometry (2 replicates), showed no altered proliferation of TCL1A expressing HH cells, allowing to exclude increased replicative stress as a main cause for the altered DDR and telomere length (net gain in genomic instability) in the presence of TCL1A.

f) Immunoblots for yH2AX in iHH / HH cells (no *ATM* sCNA, see also DSMZ catalogue #ACC707 for karyotype of HH cells) upon Etoposide-induced DSBs (50µM; 1hr) monitored over 24hrs. Doxycycline-induced TCL1A expression enhances yH2AX levels in response to DSBs induction (compare **Fig.8c**, **Supplementary Figs.17g,h** for parallel time lines of immunofluorescence (IF) microscopy based recordings of yH2AX foci).

g) IF stainings of cytospins of iHH cells (+/- Doxycycline pre-exposure) after DSB induction by Etoposide (50µM; 1hr). Enforced TCL1A expression and its impact on the kinetics of yH2AX, RAD51, and TP53BP1 focus induction and removal: delayed resolution in the presence of TCL1A. Representative images are shown (scale bar =7.5µm; overall quantifications (focus counts) and representative yH2AX time lines are in **Fig.8c**.

h) In analogy to g) here for the parental HH cells, including Doxycycline controls. Representative images and focus counts (means, SEM) are shown. In the absence of a transfected *TCL1A* overexpression construct, no difference in focus induction and resolution was detected between the +/- Doxycycline conditions.

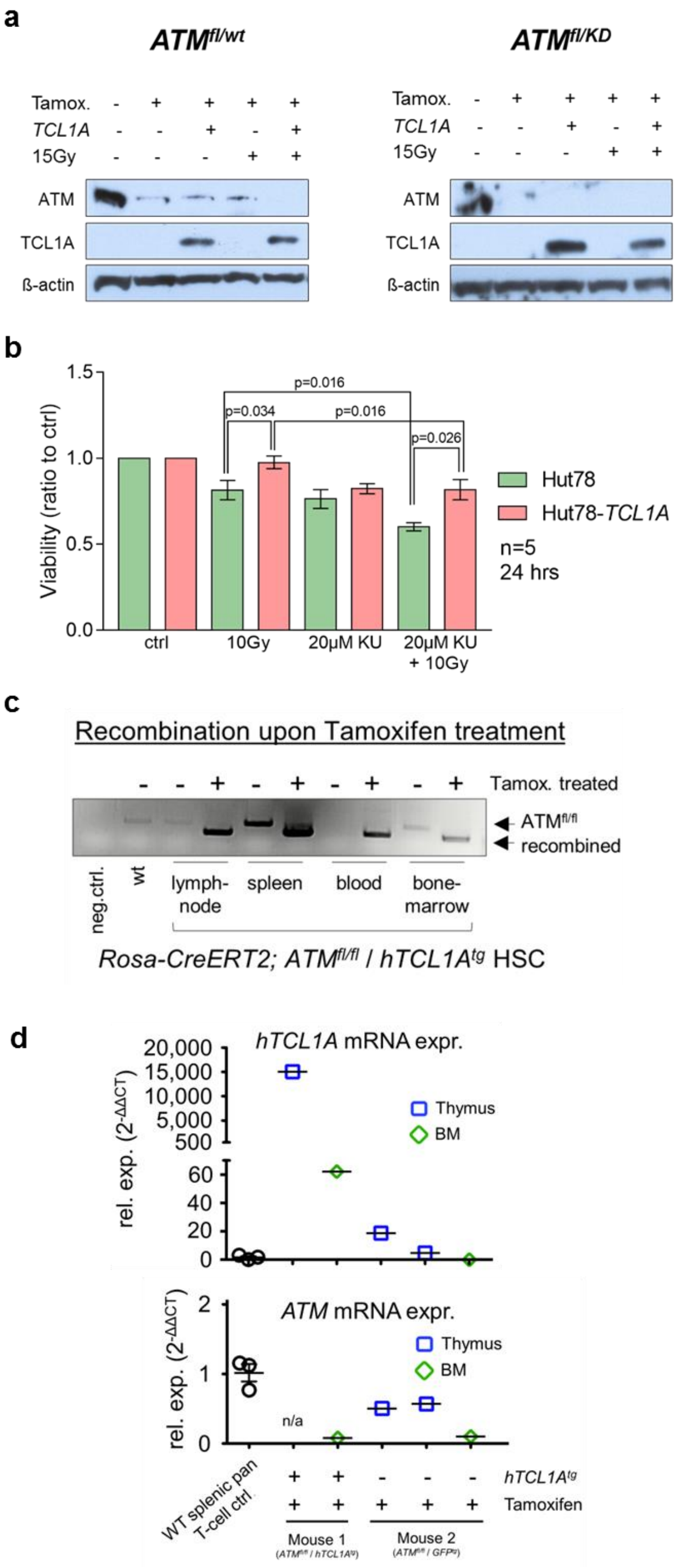

Supplementary Figure 18: Ectopic *TCL1A* overexpression cooperates with *ATM* deficiency towards accelerated T-cell lymphoma/leukemia development

legend on next page

Supplementary Figure 18: Ectopic *TCL1A* overexpression cooperates with *ATM* deficiency towards accelerated T-cell lymphoma/leukemia development

a) Immunoblots showing Tamoxifen mediated loss of ATM protein and experimentally introduced TCL1A overexpression in immortalized murine embryonic fibroblasts (MEFs). These MEFs were derived from mice carrying transgenes for the conditional expression of *ATM* variants, namely *Rosa-Cre^{ERT2};ATM$^{fl/wt}$* (for monoallelic loss) and *Rosa-Cre^{ERT2};ATM$^{fl/KD}$* (for exclusive expression PI3-kinase dead (KD) mutated ATM after loss of 'floxed' allele)[27] (data supplementing **Fig.8d**).

b) Hut78 and Hut78-TCL1 T-cell leukemia cells were treated with 20µM KU55933 and subsequently subjected to y-irradiation (10Gy) or subjected to only either of these conditions. Viability was measured after 24hrs using the MTT assay. TCLA expression conveys a survival benefit in response to irradiation, particularly in the context of ATM kinase inhibition (mean with SEM, paired Student´s t-test).

c) Data supplementing **Fig.8e**: PCR results from animals that were taken out from observation right after the end of tamoxifen injections. Neg. ctrl.: non-template H_20 ctrl.; wt: B6/C57J splenocytes. The shorter PCR product indicates successful recombination at the *Rosa-CreERT2;ATM$^{fl/fl}$* locus.

d) qRT-PCRs of 2 tumor bearing mice: mouse 1 (*ATM$^{fl/fl}$/hTCL1A^{tg}* Tamoxifen treated) and mouse 2 (*ATM$^{fl/fl}$/GFP^{tg}* Tamoxifen treated). A higher *hTCL1A* mRNA (top) and a lower *ATM* mRNA (bottom) expression was seen according to the targeted alleles in comparison to WT T-cells. Bone marrow (BM) represents non-tumor bearing hematopoietic tissue and thymus represents tumor tissue of the analyzed diseased mice transplanted with *ATM$^{fl/fl}$/hTCL1A^{tg}* or *ATM$^{fl/fl}$/GFP^{tg}* hematopoetic stem cells. This also speaks to the T-lineage specificity of the leukemogenic TCL1/ATM cooperation.

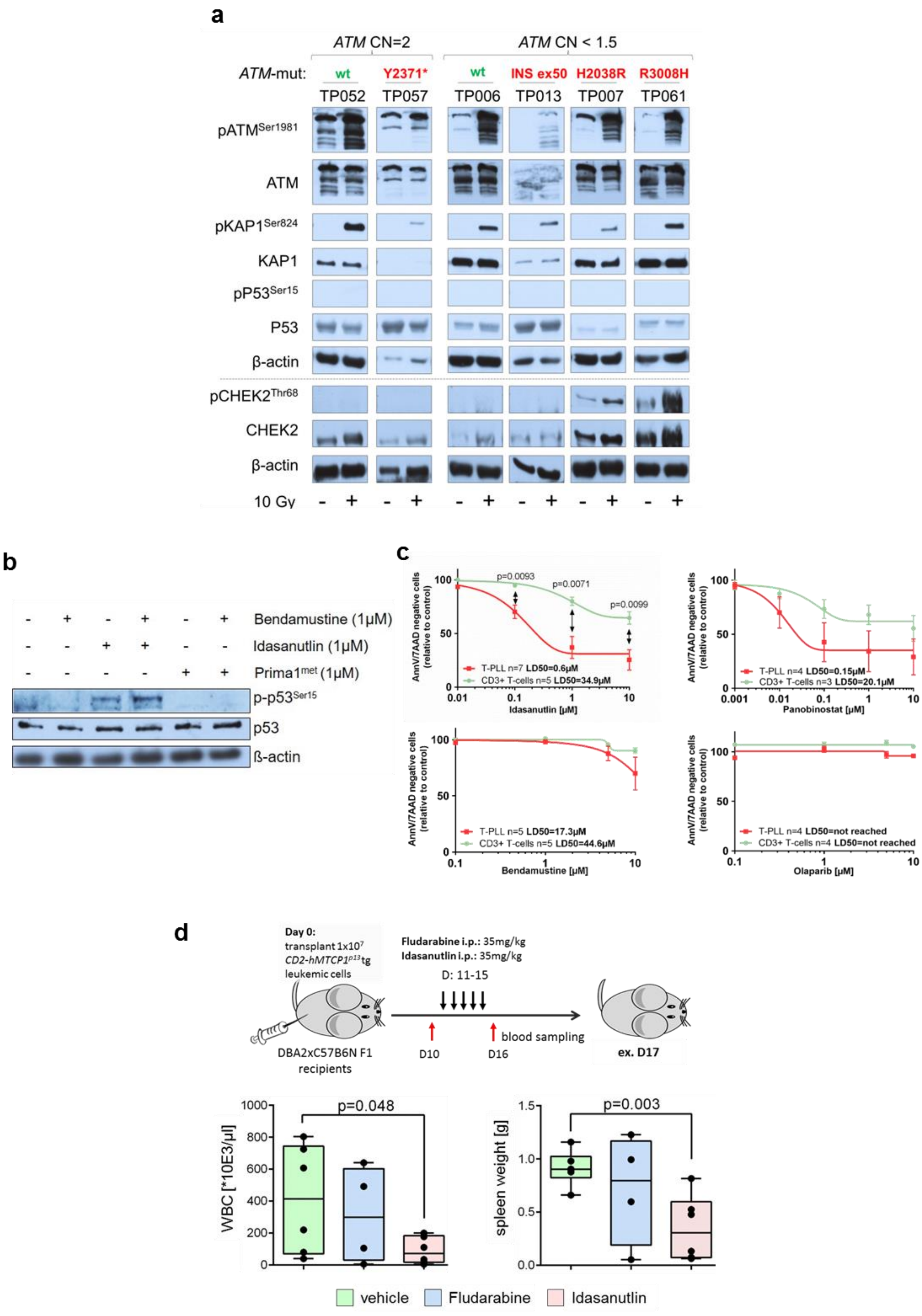

Supplementary Figure 19: Exploitation of the deficient ATM/CHEK2/p53 axis

legend on next page

Supplementary Figure 19: Exploitation of the deficient ATM/CHEK2/p53 axis

a) Phosphorylation of $ATM^{Ser1981}$, $KAP1^{Ser824}$, $CHEK2^{Thr68}$ and $p53^{Ser15}$ upon 10Gy ionizing irradiation (IR) in T-PLL cells (n=6 cases). pCHEK2 activation is only observed in 2 cases, irrespective of genomic *ATM* status or of the preserved upstream pATM induction. Median purity of T-cells 97.5%; lanes separated for genotype-based arrangement. Please note that due to sample exhaustion only these 6 cases could be analyzed for pCHEK2. Two independent immunoblots are separated by a dashed line. For completion, the corresponding data of these cases on ATM, KAP1, and p53 from **Fig.9b** and **Supplementary Fig.16b** are included here as well.

b) Primary T-PLL cells were treated with Idasanutlin, $Prima1^{met}$, Bendamustine, and combinations (all at 1µM) for 24hrs *in vitro*. Phospho-activation of p53, as detected via immunoblots, was seen in Idasanutlin conditions. In line with the *TP53* wt status of most T-PLL cases, no de-repression of p53 was observed upon $Prima1^{met}$.

c) Primary T-PLL cells (n=4-7 cases) were exposed to increasing concentrations of Idasanutlin, Panobinostat, Bendamustine, and Olaparib (ranges between 0.001-10µM) for 48hrs. Idasanutlin and Panobinostat selectively induced apoptosis in primary T-PLL cells (Idasanutlin LD50=0.6µM, Panobinostat LD50=0.15µM); healthy-donor derived pan-T-cells were less affected. Bendamustine did only induce apoptosis at higher concentrations (LD50=17.3µM) and Olaparib did not show any apoptotic effects in T-PLL cells (LD50=not reached). Cell death was quantified by AnnexinV/7AAD flow cytometry (means, SEM; Student´s t-test for single concentrations 0.01-10µM comparing T-PLL *vs.* healthy donor derived T-cells).

d) System of syngeneic transplants of leukemic cells from the *CD2-hMTCP1^{p13}* transgenic murine T-PLL model. Top: Scheme of scheduling and dosing (further details in **Methods**). Bottom: Leukemic burden and progression were suppressed by Idasanutlin, as leukocyte counts (p=0.048) and spleen weights (mean with SEM, p=0.003, Student´s t-tests) were reduced after treatment with Idasanutlin compared to Fludarabine ('standard' nucleoside analogue in T-PLL) or to vehicle. Post-mortem spleen weights (mice uniformly sacrificed at day 17) for the 3 treatment groups (means, SEM) corroborate the findings from peripheral blood.

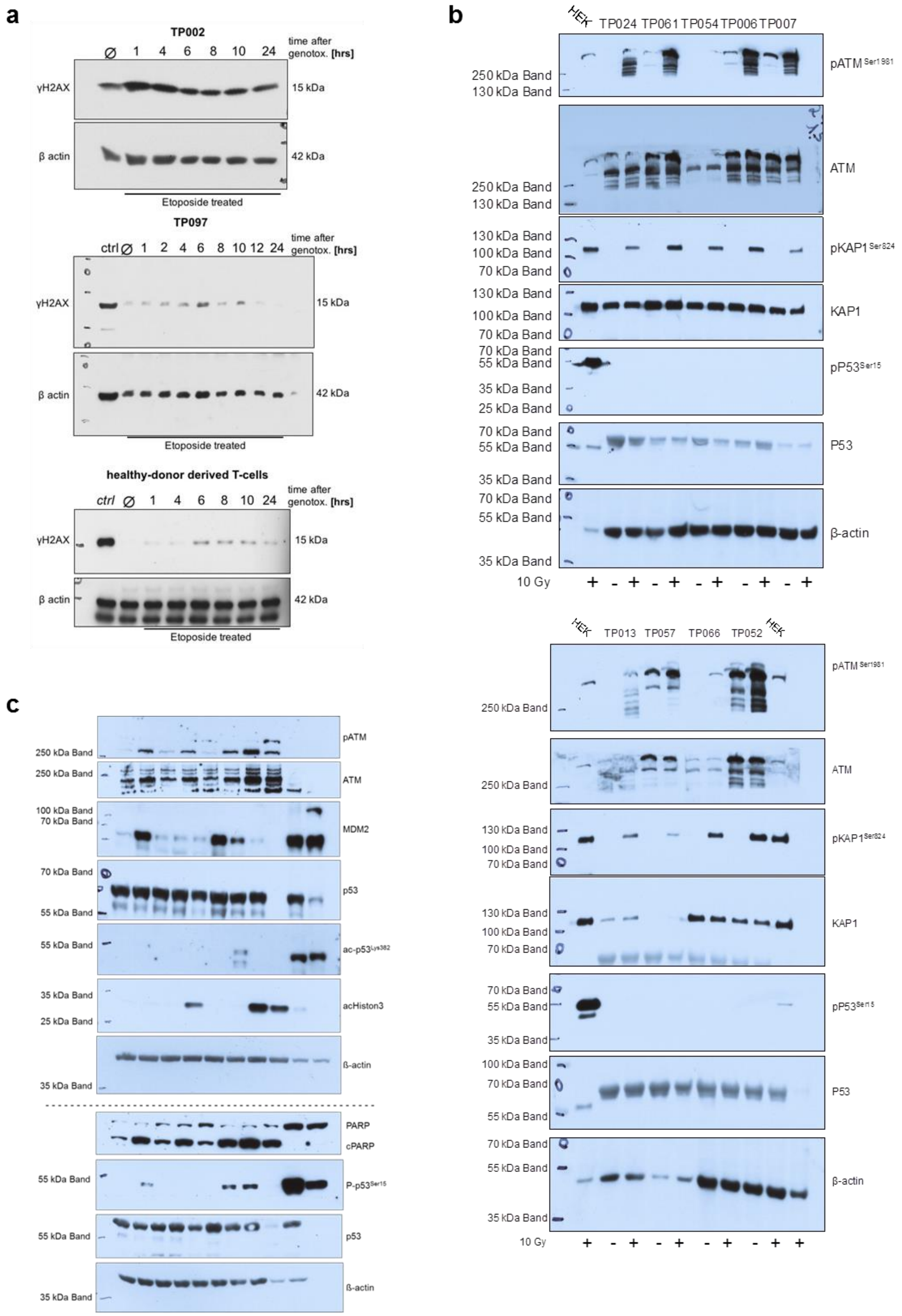

Supplementary Figure 20: Uncropped images of immunoblots

a) Immunoblots supplementing Figure 7c.

b) Immunoblots supplementing Figure 9b.

c) Immunoblots supplementing Figure 9c.

Supplementary Table 1: Profiling data in larger cohorts of T-PLL

We summarize here published studies that presented immunophenotypic, cytogenetic, genomic or transcriptomic data sets on sizable cohorts of T-PLL. Earlier studies, mostly based on clinical and flow-cytometric analyses revealed the non-descript T-cell immunophenotype of T-PLL, its dominant involvement of *TCL1A* affecting cytogenetic lesions, and the loss of *ATM* by Karyotype G-banding, FISH, and microsatellite typing[11,19,28–30]. In recent years, smaller series on gene expression profiling (GEP)[3], copy-number (CN) screens[3], targeted amplicon[20,31,32] and whole exome[16] sequencing (TAS, WES) provided isolated first fragments of genome-wide analyses.

1st Author[ref] year	# of cases	Methods	Main findings / comments
Matutes[30] 1991	78	Flow cytometry, Karyotype G-banding	IP: 65% $CD4^+$ $CD8^-$, 21% $CD4^+$ and $CD8^+$, 13% $CD4^-$ $CD8^+$; genomic abnormalities: inv(14) with breakpoints at 14q11 and 14q32 in 76% of cases, trisomy 8 in 53% of cases
Stilgenbauer[19] 1997	24	Karyotype G-banding, FISH, Sanger seq.	identification of a small commonly deleted segment at 11q22.3-23.1 (*ATM*) in 63% with mutations on the remaining allele in 25% of cases
Stoppa-Lyonnet[28] 1998	15*	LOH by microsatellite typing	inactivation of the *ATM* gene in 67% of cases through LOH
Hetet[29] 2000	21*	LOH by microsatellite typing	loss of heterozygosity of the 12p13 region, including the *ETV6* and *CDKN1B* genes in 43% of cases
Soulier[33] 2001	22	Array CGH	complex pattern of recurrent chromosomal losses and gains at e.g. 8p (86% of cases), 11q (68%), 22q11 (45%), 13q (41%), 8q (82%), 14q32 (50%)
Bradshaw[17] 2002	17	Cloning breakpoints within the *ATM* gene, Southern blot	identification of breakpoints within the *ATM* gene at the RGYW somatic hypermutation motif in 18% of cases
Dürig[3] 2007	5	GEP, SNP-arrays	differentially expressed genes enriched in genomic regions affected by recurrent chromosomal lesions (6p, 8q 6q, 8p, 10p, 11q, and 18p)
Herling[11] 2008	86	Flow cytometry and Karyotype G-banding	IP: 62% $CD4^+$ $CD8^-$, 35% $CD4^+$ and $CD8^+$, 4% $CD4^-$ $CD8^+$; genomic abnormalities: inv(14)(q11;q32.1) or t(14;14) in 40%, trisomy 8 in 35%, -11 or deletion 11q22-23 in 33%, and -17 or isochromosome 17q or deletion 17p in 13% of cases
Le Toriellec[34] 2008	47	Microsatellite typing, Sanger seq.	haploinsufficiency of *CDKN1B* in 43% of cases (partially based on data from *Soulier et al. 2001*[33])
Bug[35] 2009	12	Karyotype G-banding, GEP, SNP array, FISH	recurrent loss, but lack of mutations, of the *SMARCB1* tumor suppressor gene in 33% of cases
Delgado[36] 2012	-	Review, meta-data	update on molecular and cytogenetic abnormalities
Bellanger[31] 2014	45	Sanger seq.	recurrent *JAK1*/*JAK3* somatic mutations in 49% of cases
Bergmann[32] 2014	32	FISH, Sanger seq.	mutations of *JAK3* in 30% of cases
His[37] 2014	25	Karyotype G-banding, FISH	frequent *TCL1A* rearrangements (75% of cases), losses of *ATM* (64%), and gains of *MYC* (67%)
Kiel[16] 2014	50	WGS, WES, SNP-arrays, Sanger seq.	mutations affecting *EZH2*, *FBXW10,* and *CHEK2*; *JAK/STAT* pathway components affected in 76% of cases
Stengel[20] 2015	51	Karyotype G-banding, FISH, array CGH, amplicon NGS, Sanger seq.	deletions of *ATM* (69% of cases) and TP53 (31%); mutations in *ATM* (73%), *TP53* (14%), *JAK1* (6%), *JAK3* (21%)
López[38] 2016	43	Targeted seq. of *JAK/STAT* genes via Sanger seq.; additional 54-gene panel (recurrently mutated in hematological cancers) by amplicon NGS	activating mutations in *JAK3* (30%) and *STAT5B* (21%) in evaluated hot-spot regions, mutations in genes encoding for epigenetic regulators (*EZH2* 13%; *TET2* 17%; *BCOR* 9%)
Hu[39] 2017	97	Karyotype G-banding, FISH	correlation of cytogenetic abnormalities with clinical outcome: ≥5 aberrations associated with worse overall survival

Summary on profiling studies in T-PLL. *paired tumor germline samples; IP – Immunophenotype; LOH – loss of heterozygosity, CGH – comparative genomic hybridization, GEP – gene expression profiling, SNP – single-nucleotide polymorphism, FISH – fluorescence in situ hybridization, NGS – next-generation sequencing, WES – whole-exome sequencing, WGS – whole-genome sequencing

Supplementary Table 2: Datasets, databases, and bioinformatics tools

We summarize here the utilized bioinformatics tools, datasets, and databases including their literature reference.

**Datasets**		
**GEO ID**		**1st Author[ref] year**
GSE36363		Wilkerson[80] 2012
GSE25016		Weiss[81] 2010
GSE50253		Boi[82] 2013
GSE23452		Parkin[83] 2010
GSE21990		Ernst[84] 2010
GSE34171		Monti[85] 2012
GSE36908		Edelmann[86] 2012
**Databases**		
**Name**	**Version**	**1st Author[ref] year**
GEO	-	Barret[87] 2013
HapMap	Release 35	Intern. HapMap Project[88]
NCBI dbSNP	138	Sherry[89] 2001
COSMIC	SV 2014-02-04	Forbes[90] 2015
COSMIC	WGS 70	Forbes[91] 2011
1000G	April 2012	Abecasis[92] 2012
NCI60	2015-06-08	Abaan[93] 2013
ESP6500-SI	2015-06-08	Exome Seq. Project[94]
ExAc	0.3	Lek[95] 2016
ClinVar	2015-03-30	Landrum[96] 2014
DGV	GRCh37_hg19_variants_2013-07-23	MacDonald[97] 2014
lncRNAsv7	2012-05-02	Cabili[98] 2011
miRBase	20	Kozomara[99] 2014
FANTOM5	Phase 1 & 2	Lizio[100] 2015
TCGASpliceSeq	Accessed: 2017-06-12	Ryan[101] 2016
**Bioinformatic tools**		
**Name**	**Version**	**1st Author[ref] year**
ComBat / sva	3.22.0	Johnson[40] 2007
biomaRt	2.30.0	Durinck[41] 2005
Bioconductor	2.10	Gentleman[42] 2004
ConsensusPathDB	Release 32 (2017-01-11)	Kamburov[43] 2013
Integrative Genome Viewer	2.3.23	Thorvaldsdóttir[44] 2013
GISTIC	2.0	Mermel[8] 2011
bwa	0.6.2	Li[45] 2009
MuSiC	0.4	Dees[46] 2012
MuTect	1.14 / 2	Cibulskis[47] 2013
VarScan	2.3.6	Koboldt[48] 2012
Genome Analysis Toolkit UnifiedGenotyper	2.7-4	McKenna[49] 2010
ANNOVAR	2015-12-14	Wang[50] 2010
PROVEAN	1.1.5	Choi[51] 2012
PolyPhen-2	2	Adzhubei[15] 2013
SIFT2	2	Kumar[14] 2009
Delly	0.7.2	Rausch[52] 2012

**Bioinformatic tools** (continued)		
Circos	0.64	Krzywinski[53] 2009
MSIsensor	0.2	Niu[54] 2014
telseq	1.0	Ding[24] 2014
TopHat	2.0.10	Trapnell[55] 2009
DEXSeq	1.14.0	Anders[56] 2010
DESeq	1.16.0	Anders[57] 2012
TopHat-Fusion	2.0.10	Kim[58] 2011
Oncofuse	1.0.9	Shugay[59] 2013
STAR/STAR-Fusion	2.5.2a	Dobin[60] 2013
VirusFinder	2.0	Wang[61] 2013
PyClone	0.13.0	Roth[62] 2014
QuickNGS	1.24	Wagle[63] 2015
Ingenuity® Pathway Analysis	-	Krämer[64] 2014
Broad GSEA	2-2.2.1	Subramanian[1] 2007
STRINGdb	9.10	Franceschini[65] 2013
affy	1.52.0	Gautier[66] 2004
GenomicRanges	1.16.4	Lawrence[67] 2013
mouseDivGeno	1.0.4	Yang[68] 2009
synergyfinder	1.0	Ianevski[69] 2017
SAMtools	0.1.19	Li[70] 2009
Picard Tools	1.88	DePristo[71] 2011
liftOver	Linux-x64	Kuhn[72] 2013
EXCAVATOR2	1.1	D'Aurizio[73]
"Significance analysis of microarrays" (SAM)	-	Tusher[74] 2001
Linear regression	-	Chen[75] 2001
Tukey's median polish	-	Tukey[76] 1977
Circular binary segmentation (CBS)	-	Venkatraman[77] 2007
Birdseed	-	Korn[78] 2008
FoxoG filter	-	Costello[9] 2013
Allelic imbalance filter	-	Chen[79] 2017

Supplementary Table 3: Small molecules and compounds

Key information on the small-molecules and compounds used in this study.

Name	Target	1st Author[ref] year	Source
Idasanutlin	MDM2	Ding[102] 2013	Hycultec
Prima-1^{met}	p53	Zandi[103] 2011	GENTAUR
Panobinostat	pan-HDACs	Scuto[104] 2008	Hycultec
KU55933	ATM	Li[105] 2010	Selleckchem
Olaparib	PARP	Menear[106] 2008	Selleckchem
Bendamustine	DNA damage inducer	Leoni[107] 2008	Astellas Pharma

SUPPLEMENTARY REFERENCES

1. Subramanian, A. *et al.* Gene set enrichment analysis: a knowledge-based approach for interpreting genome-wide expression profiles. *Proc. Natl. Acad. Sci. U. S. A.* **102,** 15545–50 (2005).
2. Mootha, V. K. *et al.* PGC-1alpha-responsive genes involved in oxidative phosphorylation are coordinately downregulated in human diabetes. *Nat. Genet.* **34,** 267–73 (2003).
3. Dürig, J. *et al.* Combined single nucleotide polymorphism-based genomic mapping and global gene expression profiling identifies novel chromosomal imbalances, mechanisms and candidate genes important in the pathogenesis of T-cell prolymphocytic leukemia with inv(14)(q11q32). *Leukemia* **21,** 2153–63 (2007).
4. Schlosser, I. *et al.* Dissection of transcriptional programmes in response to serum and c-Myc in a human B-cell line. *Oncogene* **24,** 520–4 (2005).
5. Rashi-Elkeles, S. *et al.* Parallel induction of ATM-dependent pro- and antiapoptotic signals in response to ionizing radiation in murine lymphoid tissue. *Oncogene* **25,** 1584–92 (2006).
6. Jackson-Grusby, L. *et al.* Loss of genomic methylation causes p53-dependent apoptosis and epigenetic deregulation. *Nat. Genet.* **27,** 31–9 (2001).
7. Saitou, M., Sugimoto, J., Hatakeyama, T., Russo, G. & Isobe, M. Identification of the TCL6 genes within the breakpoint cluster region on chromosome 14q32 in T-cell leukemia. *Oncogene* **19,** 2796–802 (2000).
8. Mermel, C. H. *et al.* GISTIC2.0 facilitates sensitive and confident localization of the targets of focal somatic copy-number alteration in human cancers. *Genome Biol.* **12,** R41 (2011).
9. Costello, M. *et al.* Discovery and characterization of artifactual mutations in deep coverage targeted capture sequencing data due to oxidative DNA damage during sample preparation. *Nucleic Acids Res.* **41,** e67 (2013).
10. Alexandrov, L. B. *et al.* Signatures of mutational processes in human cancer. *Nature* **500,** 415–21 (2013).
11. Herling, M. *et al.* High TCL1 expression and intact T-cell receptor signaling define a hyperproliferative subset of T-cell prolymphocytic leukemia. *Blood* **111,** 328–337 (2008).
12. Prinz, C. *et al.* Organometallic nucleosides induce non-classical leukemic cell death that is mitochondrial-ROS dependent and facilitated by TCL1-oncogene burden. *Mol. Cancer* **14,** 114 (2015).
13. De Bont, R. & van Larebeke, N. Endogenous DNA damage in humans: a review of quantitative data. *Mutagenesis* **19,** 169–85 (2004).
14. Kumar, P., Henikoff, S. & Ng, P. C. Predicting the effects of coding non-synonymous variants on protein function using the SIFT algorithm. *Nat. Protoc.* **4,** 1073–81 (2009).
15. Adzhubei, I., Jordan, D. M. & Sunyaev, S. R. Predicting functional effect of human missense mutations using PolyPhen-2. *Curr. Protoc. Hum. Genet.* **Chapter 7,** Unit7.20 (2013).

16. Kiel, M. J. *et al.* Integrated genomic sequencing reveals mutational landscape of T-cell prolymphocytic leukemia. *Blood* **124(9),** 1460–72 (2014).
17. Bradshaw, P., Condie, A. & Matutes, E. Breakpoints in the ataxia telangiectasia gene arise at the RGYW somatic hypermutation motif. *Oncogene* **58,** 483–487 (2002).
18. Vořechovský, I. *et al.* Clustering of missense mutations in the ataxia-telangiectasia gene in a sporadic T-cell leukaemia. *Nat. Genet.* **17,** 96–99 (1997).
19. Stilgenbauer, S. *et al.* Biallelic mutations in the ATM gene in T-prolymphocytic leukemia. *Nat. Med.* **3,** 1155–9 (1997).
20. Stengel, A. *et al.* Genetic characterization of T-PLL reveals two major biologic subgroups and JAK3 mutations as prognostic marker. *Genes Chromosom. Cancer* **55,** 82–94 (2016).
21. Sandoval, N. *et al.* Characterization of ATM gene mutations in 66 ataxia telangiectasia families. *Hum. Mol. Genet.* **8,** 69–79 (1999).
22. Weidner, C. I. *et al.* Aging of blood can be tracked by DNA methylation changes at just three CpG sites. *Genome Biol.* **15,** R24 (2014).
23. Röth, A. *et al.* Short telomeres and high telomerase activity in T-cell prolymphocytic leukemia. *Leukemia* **21,** 2456–62 (2007).
24. Ding, Z., Mangino, M., Aviv, A., Spector, T. & Durbin, R. Estimating telomere length from whole genome sequence data. *Nucleic Acids Res.* **42,** e75 (2014).
25. Delia, D. *et al.* ATM protein and p53-serine 15 phosphorylation in ataxia-telangiectasia (AT) patients and at heterozygotes. *Br. J. Cancer* **82,** 1938–45 (2000).
26. Cawthon, R. M. Telomere length measurement by a novel monochrome multiplex quantitative PCR method. *Nucleic Acids Res.* **37,** e21 (2009).
27. Yamamoto, K. *et al.* Kinase-dead ATM protein is highly oncogenic and can be preferentially targeted by Topo-isomerase I inhibitors. *Elife* **5,** (2016).
28. Stoppa-Lyonnet, D. *et al.* Inactivation of the ATM gene in T-cell prolymphocytic leukemias. *Blood* **91,** 3920–6 (1998).
29. Hetet, G. *et al.* Recurrent molecular deletion of the 12p13 region, centromeric to ETV6/TEL, in T-cell prolymphocytic leukemia. *Hematol. J.* **1,** 42–7 (2000).
30. Matutes, E. *et al.* Clinical and laboratory features of 78 cases of T-prolymphocytic leukemia. *Blood* **78,** 3269–74 (1991).
31. Bellanger, D. *et al.* Recurrent JAK1 and JAK3 somatic mutations in T-cell prolymphocytic leukemia. *Leukemia* **28,** 417–9 (2014).
32. Bergmann, A. K. *et al.* Recurrent mutation of JAK3 in T-cell prolymphocytic leukemia. *Genes. Chromosomes Cancer* **53,** 309–16 (2014).
33. Soulier, J. *et al.* A complex pattern of recurrent chromosomal losses and gains in T-cell prolymphocytic leukemia. *Genes. Chromosomes Cancer* **31,** 248–254 (2001).
34. Le Toriellec, E. *et al.* Haploinsufficiency of CDKN1B contributes to leukemogenesis in T-cell prolymphocytic leukemia. *Blood* **111,** 2321–2328 (2008).

35. Bug, S. *et al.* Recurrent loss, but lack of mutations, of the SMARCB1 tumor suppressor gene in T-cell prolymphocytic leukemia with TCL1A-TCRAD juxtaposition. *Cancer Genet. Cytogenet.* **192,** 44–7 (2009).
36. Delgado, P., Starshak, P., Rao, N. & Tirado, C. A. A Comprehensive Update on Molecular and Cytogenetic Abnormalities in T-cell Prolymphocytic Leukemia (T-pll). *J. Assoc. Genet. Technol.* **38,** 193–8 (2012).
37. Hsi, A. C. *et al.* T-cell prolymphocytic leukemia frequently shows cutaneous involvement and is associated with gains of MYC, loss of ATM, and TCL1A rearrangement. *Am. J. Surg. Pathol.* **38,** 1468–83 (2014).
38. Lopez, C. *et al.* Genes encoding members of the JAK-STAT pathway or epigenetic regulators are recurrently mutated in T-cell prolymphocytic leukaemia. *Br. J. Haematol.* **173,** 265–273 (2016).
39. Hu, Z. *et al.* Prognostic significance of cytogenetic abnormalities in T-cell prolymphocytic leukemia. *Am. J. Hematol.* **92,** 441–447 (2017).
40. Johnson, W. E., Li, C. & Rabinovic, A. Adjusting batch effects in microarray expression data using empirical Bayes methods. *Biostatistics* **8,** 118–27 (2007).
41. Durinck, S. *et al.* BioMart and Bioconductor: a powerful link between biological databases and microarray data analysis. *Bioinformatics* **21,** 3439–40 (2005).
42. Gentleman, R. C. *et al.* Bioconductor: open software development for computational biology and bioinformatics. *Genome Biol.* **5,** R80 (2004).
43. Kamburov, A., Stelzl, U., Lehrach, H. & Herwig, R. The ConsensusPathDB interaction database: 2013 update. *Nucleic Acids Res.* **41,** D793-800 (2013).
44. Thorvaldsdóttir, H., Robinson, J. T. & Mesirov, J. P. Integrative Genomics Viewer (IGV): high-performance genomics data visualization and exploration. *Brief. Bioinform.* **14,** 178–92 (2013).
45. Li, H. & Durbin, R. Fast and accurate short read alignment with Burrows-Wheeler transform. *Bioinformatics* **25,** 1754–60 (2009).
46. Dees, N. D. *et al.* MuSiC: identifying mutational significance in cancer genomes. *Genome Res.* **22,** 1589–98 (2012).
47. Cibulskis, K. *et al.* Sensitive detection of somatic point mutations in impure and heterogeneous cancer samples. *Nat. Biotechnol.* **31,** 213–9 (2013).
48. Koboldt, D. C. *et al.* VarScan 2: somatic mutation and copy number alteration discovery in cancer by exome sequencing. *Genome Res.* **22,** 568–76 (2012).
49. McKenna, A. *et al.* The Genome Analysis Toolkit: a MapReduce framework for analyzing next-generation DNA sequencing data. *Genome Res.* **20,** 1297–303 (2010).
50. Wang, K., Li, M. & Hakonarson, H. ANNOVAR: functional annotation of genetic variants from high-throughput sequencing data. *Nucleic Acids Res.* **38,** e164 (2010).
51. Choi, Y., Sims, G. E., Murphy, S., Miller, J. R. & Chan, A. P. Predicting the functional effect of amino acid substitutions and indels. *PLoS One* **7,** e46688 (2012).
52. Rausch, T. *et al.* DELLY: structural variant discovery by integrated paired-end and split-read analysis. *Bioinformatics* **28,** i333–i339 (2012).

53. Krzywinski, M. *et al.* Circos: an information aesthetic for comparative genomics. *Genome Res.* **19,** 1639–45 (2009).
54. Niu, B. *et al.* MSIsensor: microsatellite instability detection using paired tumor-normal sequence data. *Bioinformatics* **30,** 1015–6 (2014).
55. Trapnell, C., Pachter, L. & Salzberg, S. L. TopHat: discovering splice junctions with RNA-Seq. *Bioinformatics* **25,** 1105–11 (2009).
56. Anders, S. & Huber, W. Differential expression analysis for sequence count data. *Genome Biol.* **11,** R106 (2010).
57. Anders, S., Reyes, A. & Huber, W. Detecting differential usage of exons from RNA-seq data. *Genome Res.* **22,** 2008–17 (2012).
58. Kim, D. & Salzberg, S. L. TopHat-Fusion: an algorithm for discovery of novel fusion transcripts. *Genome Biol.* **12,** R72 (2011).
59. Shugay, M., Ortiz de Mendíbil, I., Vizmanos, J. L. & Novo, F. J. Oncofuse: a computational framework for the prediction of the oncogenic potential of gene fusions. *Bioinformatics* **29,** 2539–46 (2013).
60. Dobin, A. *et al.* STAR: ultrafast universal RNA-seq aligner. *Bioinformatics* **29,** 15–21 (2013).
61. Wang, Q., Jia, P. & Zhao, Z. VirusFinder: software for efficient and accurate detection of viruses and their integration sites in host genomes through next generation sequencing data. *PLoS One* **8,** e64465 (2013).
62. Roth, A. *et al.* PyClone: statistical inference of clonal population structure in cancer. *Nat. Methods* **11,** 396–398 (2014).
63. Wagle, P., Nikolić, M. & Frommolt, P. QuickNGS elevates Next-Generation Sequencing data analysis to a new level of automation. *BMC Genomics* **16,** 487 (2015).
64. Krämer, A., Green, J., Pollard, J. & Tugendreich, S. Causal analysis approaches in Ingenuity Pathway Analysis. *Bioinformatics* **30,** 523–530 (2014).
65. Franceschini, A. *et al.* STRING v9.1: protein-protein interaction networks, with increased coverage and integration. *Nucleic Acids Res.* **41,** D808-15 (2013).
66. Gautier, L., Cope, L., Bolstad, B. M. & Irizarry, R. A. affy--analysis of Affymetrix GeneChip data at the probe level. *Bioinformatics* **20,** 307–15 (2004).
67. Lawrence, M. S. *et al.* Mutational heterogeneity in cancer and the search for new cancer-associated genes. *Nature* **499,** 214–8 (2013).
68. Yang, H. *et al.* A customized and versatile high-density genotyping array for the mouse. *Nat. Methods* **6,** 663–6 (2009).
69. Ianevski, A., He, L., Aittokallio, T. & Tang, J. SynergyFinder: a web application for analyzing drug combination dose–response matrix data. *Bioinformatics* (2017). doi:10.1093/bioinformatics/btx162
70. Li, H. *et al.* The Sequence Alignment/Map format and SAMtools. *Bioinformatics* **25,** 2078–2079 (2009).
71. DePristo, M. A. *et al.* A framework for variation discovery and genotyping using next-generation DNA sequencing data. *Nat. Genet.* **43,** 491–8 (2011).
72. Kuhn, R. M., Haussler, D. & Kent, W. J. The UCSC genome browser and associated tools. *Brief. Bioinform.* **14,** 144–161 (2013).

73. D'Aurizio, R. *et al.* Enhanced copy number variants detection from whole-exome sequencing data using EXCAVATOR2. *Nucleic Acids Res.* **44,** e154 (2016).
74. Tusher, V. G., Tibshirani, R. & Chu, G. Significance analysis of microarrays applied to the ionizing radiation response. *Proc. Natl. Acad. Sci. U. S. A.* **98,** 5116–21 (2001).
75. Chen, K. Generalized case-cohort sampling. *J. R. Stat. Soc. Ser. B (Statistical Methodol.* **63,** 791–809 (2001).
76. Tukey, J. W. *Exploratory Data Analysis*. (Addison-Wesley, 1977).
77. Venkatraman, E. S. & Olshen, A. B. A faster circular binary segmentation algorithm for the analysis of array CGH data. *Bioinformatics* **23,** 657–63 (2007).
78. Korn, J. M. *et al.* Integrated genotype calling and association analysis of SNPs, common copy number polymorphisms and rare CNVs. *Nat. Genet.* **40,** 1253–60 (2008).
79. Chen, L., Liu, P., Evans, T. C. & Ettwiller, L. M. DNA damage is a pervasive cause of sequencing errors, directly confounding variant identification. *Science (80-.).* **355,** 752–756 (2017).
80. Wilkerson, M. D. *et al.* Differential pathogenesis of lung adenocarcinoma subtypes involving sequence mutations, copy number, chromosomal instability, and methylation. *PLoS One* **7,** e36530 (2012).
81. Weiss, J. *et al.* Frequent and focal FGFR1 amplification associates with therapeutically tractable FGFR1 dependency in squamous cell lung cancer. *Sci. Transl. Med.* **2,** 62ra93 (2010).
82. Boi, M. *et al.* PRDM1/BLIMP1 is commonly inactivated in anaplastic large T-cell lymphoma. *Blood* **122,** 2683–93 (2013).
83. Parkin, B. *et al.* Acquired genomic copy number aberrations and survival in adult acute myelogenous leukemia. *Blood* **116,** 4958–67 (2010).
84. Ernst, T. *et al.* Inactivating mutations of the histone methyltransferase gene EZH2 in myeloid disorders. *Nat. Genet.* **42,** 722–6 (2010).
85. Monti, S. *et al.* Integrative analysis reveals an outcome-associated and targetable pattern of p53 and cell cycle deregulation in diffuse large B cell lymphoma. *Cancer Cell* **22,** 359–72 (2012).
86. Edelmann, J. *et al.* High-resolution genomic profiling of chronic lymphocytic leukemia reveals new recurrent genomic alterations. *Blood* **120,** 4783–94 (2012).
87. Barrett, T. *et al.* NCBI GEO: archive for functional genomics data sets--update. *Nucleic Acids Res.* **41,** D991-5 (2013).
88. The International HapMap Project. *Nature* **426,** 789–96 (2003).
89. Sherry, S. T. *et al.* dbSNP: the NCBI database of genetic variation. *Nucleic Acids Res.* **29,** 308–11 (2001).
90. Forbes, S. A. *et al.* COSMIC: exploring the world's knowledge of somatic mutations in human cancer. *Nucleic Acids Res.* **43,** D805–D811 (2015).
91. Forbes, S. A. *et al.* COSMIC: mining complete cancer genomes in the Catalogue of Somatic Mutations in Cancer. *Nucleic Acids Res.* **39,** D945-50 (2011).

92. Abecasis, G. R. *et al.* An integrated map of genetic variation from 1,092 human genomes. *Nature* **491,** 56–65 (2012).
93. Abaan, O. D. *et al.* The exomes of the NCI-60 panel: a genomic resource for cancer biology and systems pharmacology. *Cancer Res.* **73,** 4372–82 (2013).
94. Exome Variant Server. NHLBI Exome Sequencing Project (ESP). *Seattle, WA Retrieved June, 2015, from http//evs.gs.washington.edu/EVS/*
95. Lek, M. *et al.* Analysis of protein-coding genetic variation in 60,706 humans. *Nature* **536,** 285–291 (2016).
96. Landrum, M. J. *et al.* ClinVar: public archive of relationships among sequence variation and human phenotype. *Nucleic Acids Res.* **42,** D980-5 (2014).
97. MacDonald, J. R., Ziman, R., Yuen, R. K. C., Feuk, L. & Scherer, S. W. The Database of Genomic Variants: a curated collection of structural variation in the human genome. *Nucleic Acids Res.* **42,** D986-92 (2014).
98. Cabili, M. N. *et al.* Integrative annotation of human large intergenic noncoding RNAs reveals global properties and specific subclasses. *Genes Dev.* **25,** 1915–27 (2011).
99. Kozomara, A. & Griffiths-Jones, S. miRBase: annotating high confidence microRNAs using deep sequencing data. *Nucleic Acids Res.* **42,** D68-73 (2014).
100. Lizio, M. *et al.* Gateways to the FANTOM5 promoter level mammalian expression atlas. *Genome Biol.* **16,** 22 (2015).
101. Ryan, M. *et al.* TCGASpliceSeq a compendium of alternative mRNA splicing in cancer. *Nucleic Acids Res.* **44,** D1018-22 (2016).
102. Ding, Q. *et al.* Discovery of RG7388, a Potent and Selective p53–MDM2 Inhibitor in Clinical Development. *J. Med. Chem.* **56,** 5979–5983 (2013).
103. Zandi, R. *et al.* PRIMA-1Met/APR-246 induces apoptosis and tumor growth delay in small cell lung cancer expressing mutant p53. *Clin. Cancer Res.* **17,** 2830–41 (2011).
104. Scuto, A. *et al.* The novel histone deacetylase inhibitor, LBH589, induces expression of DNA damage response genes and apoptosis in Ph- acute lymphoblastic leukemia cells. *Blood* **111,** 5093–100 (2008).
105. Li, Y. & Yang, D.-Q. The ATM inhibitor KU-55933 suppresses cell proliferation and induces apoptosis by blocking Akt in cancer cells with overactivated Akt. *Mol. Cancer Ther.* **9,** 113–25 (2010).
106. Menear, K. A. *et al.* 4-[3-(4-cyclopropanecarbonylpiperazine-1-carbonyl)-4-fluorobenzyl]-2H-phthalazin-1-one: a novel bioavailable inhibitor of poly(ADP-ribose) polymerase-1. *J. Med. Chem.* **51,** 6581–91 (2008).
107. Leoni, L. M. *et al.* Bendamustine (Treanda) displays a distinct pattern of cytotoxicity and unique mechanistic features compared with other alkylating agents. *Clin. Cancer Res.* **14,** 309–17 (2008).
