# Supplementary material for: Actionable perturbations of damage responses by TCL1/ATM and epigenetic lesions form the basis of T-PLL
Source: Nat Commun. 2018 Feb 15;9:697. doi: 10.1038/s41467-017-02688-6 (PMC5814445; doi:10.1038/s41467-017-02688-6)
Supplement: Supplementary file 2 — Description of Additional Supplementary Files [file 41467_2017_2688_MOESM2_ESM.pdf]

Description of Additional Supplementary Files

File Name: Supplementary Data 1
Description: **Cohort of analyzed T-PLL cases** Samples from 98 patients were subjected to genomic analyses, but an overall of 111 patients were included in this study accounting also for those on which only in vitro experimentation was performed. Information on age, sex, immunophenotype, karyotype, and white blood cell counts are included.

File Name: Supplementary Data 2
Description: **Differentially expressed genes comparing T-PLL cases to healthy-donor derived T-cells analyzed by gene expression profiling** Differentially expressed genes (fc>=1.5; q<=0.05) in primary human T-PLL vs. normal peripheral blood (PB) T-cells analyzed via using Illumina HumanHT-12 v4 BeadChip arrays.

File Name: Supplementary Data 3
Description: **Differentially expressed genes comparing murine T-PLL like T-cell expansions to healthy wild-type control derived splenic T-cells and overlap of significantly expressed genes to human T-PLL as analyzed by gene expression profiling** Murine splenocytes were enriched for CD8+ lymphocytes using MACS beads. 3 control RNA samples (pooled from CD3+ T-cells enriched from 9 spleens of age- and backgroundmatched wt animals) as well as RNA isolated from CD8+ -enriched splenic T-cells of 3 “chronic phase” and of 5 “exponential phase” Lckpr-hTCL1A+/- mouse lymphoma samples were analyzed via Affymetrix Mouse Gene 1.0 ST Arrays. a) Murine T-cell leukemia at ‘exponential phase’ and at ‘chronic phase’ (n=8) vs. wt age matched controls (n=9). b) Murine T-cell leukemia at ‘chronic phase’ (n=3) vs. wt age matched controls (n=9). c) Murine T-cell leukemia at ‘exponential phase’ (n=5) vs. wt age matched controls (n=9). d) Overlap analysis of differentially expressed genes in murine vs. human T-PLL.

File Name: Supplementary Data 4
Description: **Differentially expressed genes according to WTS data set and overlaps with GEP-array based analyses** PBMCs of T-PLL patients (>95% purity of T-cells) and CD3+ T-cells isolated from PB of healthy donors were subjected to RNA isolation and subsequent WTS analyses using the Illumina HiSeq2000 platform. DESeq v1.14.0 and DEXSeq v1.16.0 algorithms were used to analyze differentially expressed and differentially spliced genes. a) Differentially expressed genes according to WTS data set. b) Overlaps with GEP-array based analyses.

File Name: Supplementary Data 5
Description: **Differentially used exons according to WTS** PBMCs of T-PLL patients (>95% purity of T-cells) and CD3+ T-cells isolated from PB of healthy donors were subjected to RNA isolation and subsequent WTS analyses using the Illumina HiSeq2000 platform. Differential exon usage was analyzed using the DEXSeq package, version 1.16.0. a) Differentially used exons according to WTS. b) Ingenuity pathway analysis of genes affected by differential exon usage.

File Name: Supplementary Data 6
Description: **GISTIC2.0 detected genomic regions significantly affected by sCNAs** DNAs were isolated from PBMCs of T-PLL patients (n=83, >95% purity of T-cells) that included 13 CD4/CD8-enriched/depleted tumor/germline (t/g) pairs. SNP-array analyses were conducted using Affymetrix SNP 6.0 chips. a) Comparison to HapMap controls. b) Comparison to patient derived controls.

File Name: Supplementary Data 7
Description: **sCNA affected genes per patient and sCNA frequencies based on T-PLL patient derived controls and HapMap controls** DNAs were isolated from PBMCs of T-PLL patients (n=83, >95% purity of T-cells) that included 13 CD4/CD8-enriched/depleted tumor/germline (t/g) pairs. SNP-array analyses were conducted using Affymetrix SNP 6.0 chips. a) sCNA frequencies across all cases. b) sCNA affected genes per patient - comparison to patient derived controls. c) sCNA affected genes per patient - comparison to HapMap controls. d) UPD affected genes per patient - comparison to patient derived controls. e) UPD affected genes per patient - comparison to HapMap controls.

File Name: Supplementary Data 8
Description: **Differentially expressed genes associated with chr.11 loss and chr.8 gain lesions** GEPs of cases carrying losses at chr.11 were compared to cases 'biallelic' for chr.11 (ATM CN2.2 vs. CN=2 according to comparison to HapMap controls; chr.11 affected cases excluded). a) Differentially expressed genes associated with chr.11 loss. b) Differentially expressed genes associated with chr.8 gain. c) Overlap of differentially expressed genes associated with chr.11 loss and chr.8 gain lesions.

File Name: Supplementary Data 9
Description: **Differentially expressed genes associated with low ATM and high AGO2 expression including overlaps of CN lesion associated genes** Differential expression of genes specifically associated with stratified ATM and AGO2 mRNA abundance; comparison: 10 T-PLL with highest vs. 10 cases with lowest expression (fc of ATM and AGO2 expression indicated). a) Differentially expressed genes associated with low ATM expression. b) Differentially expressed genes associated with low ATM: overlaps of CN lesion associated genes. c) Differentially expressed genes associated with high AGO2 expression. d) Differentially expressed genes associated with high AGO2: overlaps of CN lesion associated genes.

File Name: Supplementary Data 10
Description: **Structural variations detected by WGS and WES** We inferred structural variations by mapping distance and order of paired-end reads using DELLY (version 0.7.2) and filtered for a minimum genotype quality of 200, for no LowQual entries, minimum depth of 5 reads, and for split-read support (more precise breakpoint localization). For structural variations affecting TCL1 oncogenes, we applied more lenient filters (genotype likely somatic). CN neutral entries in the database of genomic variants (GRCh37_hg19_variants_2013-07-23) were further used to filter within a 1kb breakpoint window. The resulting list was then annotated with the COSMIC SV data sheet. a) Structural variations detected by WGS. b) Structural variations detected by WES - 17 tumor/germline pairs. c) Structural variations detected by WES - 37 tumor singles. d) Structural variations of chr.14 detected by WES - 17 tumor/germline pairs. e) Structural variations of chr.14 detected by WES - 37 tumor singles.

File Name: Supplementary Data 11
Description: **Fusion transcripts detected by WTS analyses** Fusion transcripts (n=96, TopHat-Fusion and oncofuse algorithms) identified by wholetranscriptome sequencing (WTS) of 15 T-PLL compared to healthy donor T-cells (n=4).

File Name: Supplementary Data 12
Description: **WES and WGS data sets: Mutated genes** We detected somatic single-nucleotide variants (sSNVs) using MuTect 1.1.4 and MuTect v2 with default parameters, while for somatic indels (insertions and deletions) VarScan 2.3.6 was used (a,d). Pseudosomatic SNVs and small indels were filtered (i) by exclusion of potential SNPs by eliminating mutations with a 1000G and/or ESP6500-SI frequency and/or ExAc0.3 minor allele fraction (MAF) ≥0.01 (PopFreq

File Name: Supplementary Data 13
Description: **MuSic identified genes** MuSiC was used to infer on statistical significance of identified mutations. a) MuSic identified genes in n=17 somatic paired T-PLL/normal. b) MuSic identified genes in n=37 single/potentially somatic T-PLL.

File Name: Supplementary Data 14
Description: **Highly clonal mutations: Genes mutated with a VAF >80%** Highly clonal mutations were identified by selecting for SNVs and indels with a VAF >80%.

File Name: Supplementary Data 15
Description: **Combined analysis of sCNA and SNV data: Genes affected by gain of function / loss of function alterations** Integration of sCNA and WES data to speculate on selection for dysfunctional targets affected by gain-of-function (GOF, CNV>2.2, VAF>0.5) or loss-of-function (LOF, CNV0.5) aberrations. a) Genes affected by gain of function / loss of function alterations - sCNA. b) Genes affected by gain of function / loss of function alterations - UPD.

File Name: Supplementary Data 16
Description: **Differentially expressed genes in sequential T-PLL samples** GEP of 4 cases with available F/U sample pairs. Differential expression calculated separately for each time point (vs. healthy-donor T-cells). a) Differentially expressed genes in sequential T-PLL samples t1 vs. ctrl. b) Differentially expressed genes in sequential T-PLL samples t2 vs. ctrl. c) Overlaps of differentially expressed genes in cases with available follow-up (F/U) samples (t1 vs. t2).

File Name: Supplementary Data 17
Description: **sCNAs identified in sequential T-PLL samples** sCNA of 5 cases with available F/U sample pairs. a) TP092 b) TP093 c) TP094 d) TP095 e) TP096

File Name: Supplementary Data 18
Description: **SNVs identified in sequential T-PLL samples** SNVs of 5 cases with available F/U sample pairs. a) TP092 b) TP093 c) TP094 d) TP095 e) TP096

File Name: Supplementary Data 19
Description: **Geneset Enrichment Analysis - genes mutated in F/U cases** Functional affiliations of mutated genes of 5 cases with available F/U sample pairs. a) Drop to VAF=0. b) Rise from VAF=0. c) t2 specific calls

File Name: Supplementary Data 20
Description: **Analyzed genes encoding epigenetic modifiers** Selection of analyzed genes associated with epigenetic regulations.

File Name: Supplementary Data 21
Description: **Analyzed genes encoding DDR factors** Selection of analyzed genes associated with DNA damage repair.

File Name: Supplementary Data 22
Description: **Oligonucleotides** Oligonucleotides utilized as part of this study. a) qRT-PCR b) targeted sequencing c) STAT5B sanger sequencing d) TCL1A transcript validation e) telomere length validation f) ATM genotyping
